# Insights into serum metabolic biomarkers for early detection of incident diabetic kidney disease in Chinese patients with type 2 diabetes by random forest

**DOI:** 10.18632/aging.205542

**Published:** 2024-02-12

**Authors:** Jian-Jun Jiang, Tung-Ting Sham, Xiu-Fen Gu, Chi-On Chan, Nai-Ping Dong, Wei-Han Lim, Gao-Feng Song, Shun-Min Li, Daniel Kam-Wah Mok, Na Ge

**Affiliations:** 1 Department of Nephrology, Shenzhen Traditional Chinese Medicine Hospital, The Fourth Clinical Medical College of Guangzhou University of Chinese Medicine, Shenzhen, China; 2 The Research Centre for Chinese Medicine Innovation and Department of Applied Biology and Chemical Technology, The Hong Kong Polytechnic University, Hong Kong, China; 3 State Key Laboratory of Chinese Medicine and Molecular Pharmacology (Incubation), Shenzhen, China

**Keywords:** diabetic kidney disease, biomarkers, untargeted metabolomics, random forest

## Abstract

Diabetic kidney disease (DKD) is a leading cause of end-stage renal disease (ESRD) worldwide. Early detection is critical for the risk stratification and early intervention of progressive DKD. Serum creatinine (sCr) and urine output are used to assess kidney function, but these markers are limited by their delayed changes following kidney pathology, and lacking of both sensitivity and accuracy. Hence, it is essential to illustrate potential diagnostic indicators to enhance the precise prediction of early DKD. A total of 194 Chinese individuals include 30 healthy participants (Stage 0) and 164 incidents with type 2 diabetes (T2D) spanning from DKD’s Stage 1a to 4 were recruited and their serums were subjected for untargeted metabolomic analysis. Random forest (RF), a machine learning approach, together with univariate linear regression (ULR) and multivariate linear regression (MvLR) analysis were applied to characterize the features of untargeted metabolites of DKD patients and to identify candidate DKD biomarkers. Our results indicate that 2-(α-D-mannopyranosyl)-L-tryptophan (ADT), succinyladenosine (SAdo), pseudouridine and N,N,N-trimethyl-L-alanyl-L-proline betaine (L-L-TMAP) were associated with the development of DKD, in particular, the latter three that were significantly elevated in Stage 2-4 T2D incidents. Each of the four metabolites in combination with sCr achieves better performance than sCr alone with area under the receiver operating characteristic curve (AUC) of 0.81-0.91 in predicting DKD stages. An average of 3.9 years follow-up study of another cohort including 106 Stage 2-3 patients suggested that “urinary albumin-to-creatinine ratio (UACR) + ADT + SAdo” can be utilized for better prognosis evaluation of early DKD (average AUC = 0.9502) than UACR without sexual difference.

## INTRODUCTION

Diabetic kidney disease (DKD) is the most common cause of end stage renal disease (ESRD), affecting 20–30% of diabetic patients globally [1]. Standard biomarkers including urinary albumin-to-creatinine ratio (UACR) and estimated glomerular filtration rate (eGFR) are the clinical parameters commonly used to evaluate renal function in clinical practice. However, due to the level of urinary output and serum creatinine (sCr) can be influenced by many factors, these measures are restricted as they may lack of sensitivity and accuracy [2]. Therefore, there is an urgent need to identify novel biomarkers for the diagnosis and management of DKD.

Metabolomics is a promising tool for detailed characterization of dynamic molecular changes in the intra- and inter-cellular process. It has been applied in multiple fields such as metabolism of drugs or environmental toxicants, screening for new therapeutic targets, discovery and validation of disease biomarkers [3]. Increasing evidence has revealed the association among metabolites, diabetes mellitus (DM) and diabetic complications [4]. Serum metabolic analysis of Korean T2D patients suggested that alanine, arginine, isoleucine, proline, tyrosine, valine, hexose and five phosphatidylcholine diacyls were positively associated with T2D risk [5]. For DKD prediction, Huang et al. utilized targeted metabolomics profiles to evaluate prospective metabolite predictors in the German diabetic individuals of the Region of Augsburg (KORA) cohort, and identified sphingomyelin (SM) C18:1 and phosphatidylcholine diacyl (PC aa) C38:0 as the potential metabolite biomarkers. [6]. In addition to metabolites, elements such as neutrophil gelatinase-associated lipocalin (NGAL), fatty acid-binding protein [7] and cystatin C [8] have been proposed to be correlated with the development of DKD. However, studies exploring the associations between metabolites and the DKD disease development in Chinese are very limited.

In this study, we performed untargeted metabolomics of 194 serum samples collected from 164 Chinese incidents of type 2 diabetes (T2D) and 30 healthy participants (non-T2D and non-diabetes). The metabolites identified were validated by an extra follow up cohort of 106 subjects with a mean follow-up time of 3.9 years. By assessing the predictive power of metabolites via a stringent workflow, we finally identified pseudouridine, L-L-TMAP, ADT and SAdo as the candidate predictors for early DKD.

## RESULTS

### Baseline characteristics of study participants

**Figure 1:**
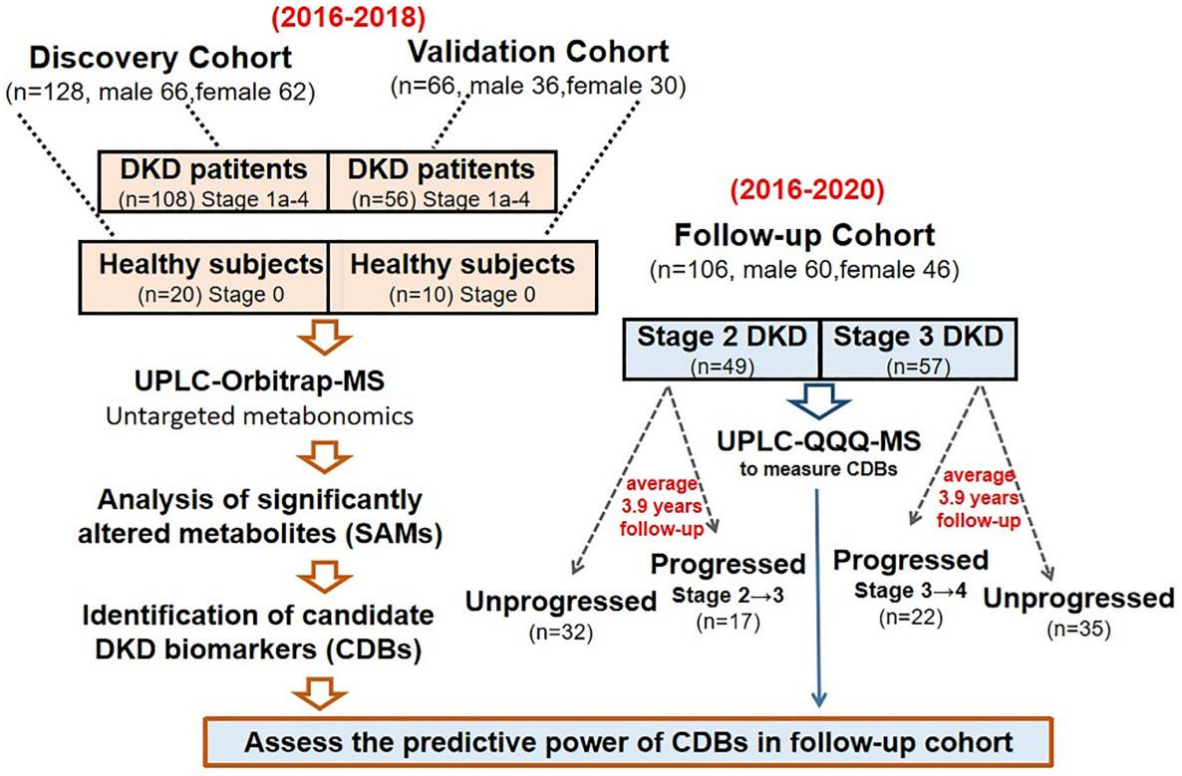
**The pipeline of this study.** Three independent cohorts were recruited to perform the metabolite biomarker in discovery, validation and follow-up groups, respectively.

An overview of this study design was shown in Figure 1. For the 164 diabetic patients in the discovery and validation cohort, their median diabetic duration was 8 years, median eGFR was 76 (43–104) mL/min/1.73 m^2^, and median UACR was 80 (ranges from 10 to 842) mg/g Cr. Approximately 67% of them had a history of ≥ one diabetic microvascular or macrovascular complication (Supplementary Table 1). The median eGFR and UACR of 30 healthy participants were 99 (95–112) mL/min/1.73 m^2^ and 2.7 (2.3–3.9) mg/g Cr., respectively. The baseline characteristics of the two cohorts were compared, the patients group showed lower eGFR and an increase of UACR, RRI, systolic blood pressure (SBP), serum creatinine, urea, uric acid, cystatin C and urinary *β*2-microglobulin (*β*2-MG) concentrations along with DKD severity (Supplementary Table 2). We noticed that Stage 1a patients showed an enlargement of kidney size in compare with healthy participants by renal ultrasound images and testing body surface-area (BSA) related renal volumes (Supplementary Figure 1); however, for patients at Stage 2–4, their BSA related renal volumes were gradually decreased with DKD progressed (Supplementary Figure 1), which is consistent with previous findings that kidneys tended to be smaller in the most advanced stages of CKD [9]. Our results suggested that patients were likely to have abnormal kidney hypertrophy and enlargement at Stage 1a, followed by gradual renal atrophy and volume depletion at later stages.

### Characterization of metabolites in study participants

A total of 7480 compounds were detected and we found that the trend of MS-detected sCr among all the participants showed remarkable consistency with clinically measured sCr (Supplementary Figure 2), suggesting that UPLC-Orbitrap-MS is accurate and efficient for high-throughout metabolites detection. Step-wise filtering was performed based on two criteria: removing metabolites with unstable signals and only retaining metabolites with significant different concentration levels between healthy control and patients, 80 candidates (72 metabolites and 8 ratios) were screened out for a next-step analysis (Supplementary Tables 3 and 4, Supplementary Figure 3). The distribution plot of preprocessed data was shown as Supplementary Figure 4. The fold changes of these metabolites among different stages were calculated and shown in Supplementary Tables 5 and 6.

**Figure 2:**
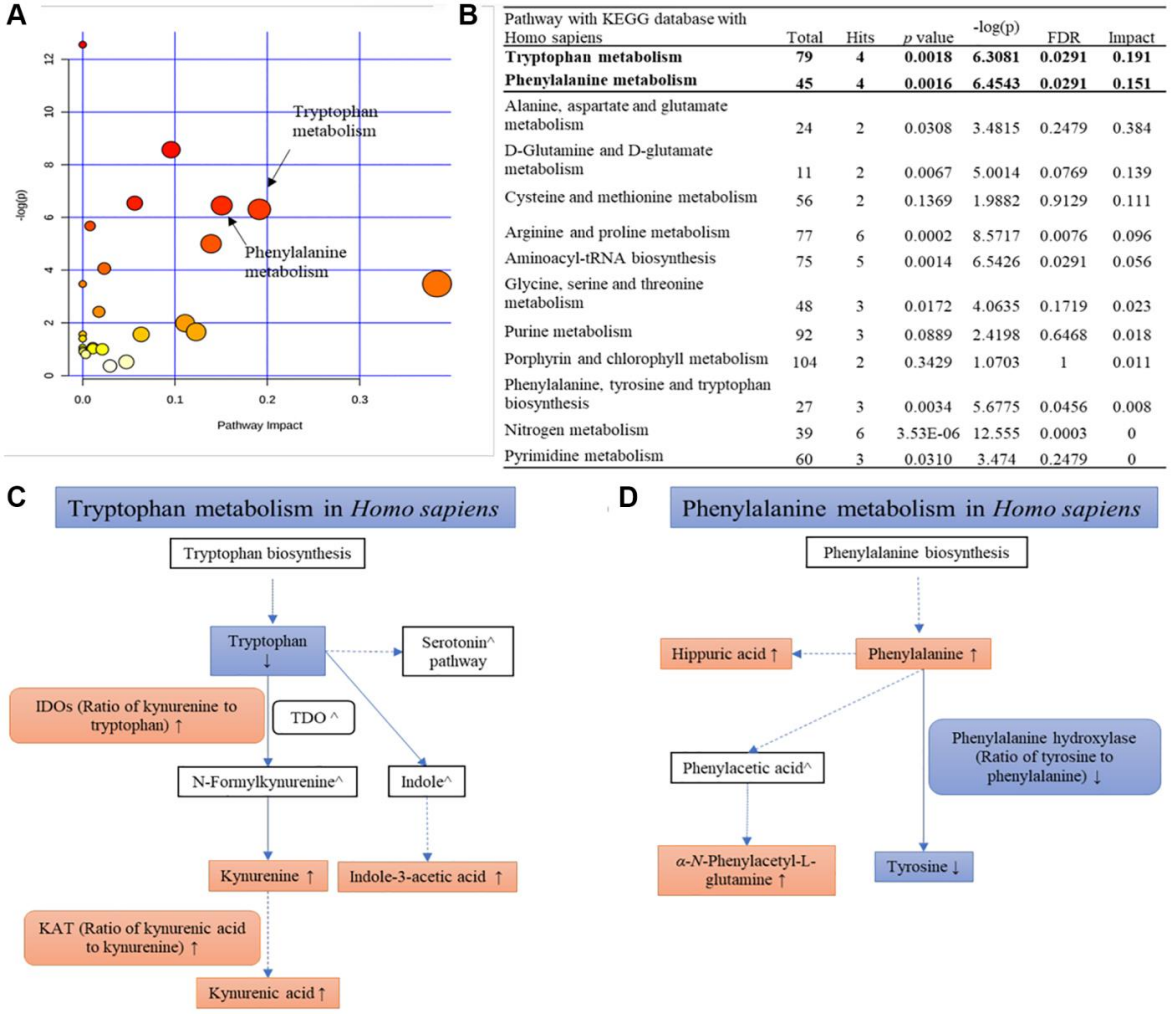
**KEGG pathway analysis of all SAMs.** (**A**) An overview view of pathway analysis; (**B**) Table of the matched pathway with *p*-values from pathway enrichment analysis and pathway impact values from the pathway topology analysis using MetaboAnalyst 4.0 and KEGG database (Hits ≥ 2); (**C**, **D**) Simplified pathways of tryptophan metabolism and phenylalanine metabolism with the change trends of metabolites and their ratios at Stage 4 compared with the normal group.

The 72 metabolites are classified to 7 categories: sulfate metabolites, amino acids, organic acids, acylcarnitine, purine derivatives, steroids and monosaccharides. Metabolomic network based on the 72 metabolites in the discovery set from Stage 0–4 was shown in Supplementary Figure 4. Comparing with healthy group (Stage 0), merely 3 down- and 6 up- regulated metabolites were found in Stage 1a; nevertheless, it increased to 7 down- and 40 up- regulated metabolites in Stage 4. The SAMs were enriched in Tryptophan metabolism (hsa00380) and Phenylalanine metabolism (hsa00360) pathways, indicating that amino acid metabolism disruption is a dominate signature of DKD (Figure 2). Among these significant altered metabolites (SAMs), 1,5-anhydro-D-glucitol (1,5-AG) was remarkably reduced in stages 1–4 compared to healthy group (fold change = −26.5 to −2.60, Supplementary Table 5). As demonstrated by previous studies that 1,5-AG is a potential biomarker for monitoring the progression of diabetes [10, 11], we therefore separately tested the correlation of two clinical glycemic markers – fast blood glucose (FBG) and hemoglobin A1C (HbA1c) with 1,5-AG in our cohort. It showed that 1,5-AG has strong negative correlation with HbA1c and FBG in stage 1a-3 patients (*r* ranges were −0.95 to −0.64 and −0.87 to −0.42, respectively); however, abnormal correlation was observed between 1,5-AG and FBG in stage 4 discovery sets, with r = 0.25 (Supplementary Figure 5A, 5B). Correlation between 1,5-AG and HbA1c was stronger among stage 1a-3 patients than stage 0–4 (Supplementary Figure 5A, 5B), suggesting that 1,5-AG may serve better as a potential glycemic marker in stage 1a-3 DKD patients than late stage.

Given that DKD is one of the consequences induced by diabetes, we hypothesized that decreased levels of 1,5-AG may be relevant with DKD development. However, our results showed that 1,5-AG exhibited non-significant correlation with neither eGFR nor UACR (Supplementary Figure 5C), indicating that diabetic progression has limited contribution to DKD development (scatter plot of 1,5-AG levels among healthy controls and different stages of patients was shown in Supplementary Figure 5D).

### Identification of candidate DKD biomarkers (CDBs)

**Figure 3:**
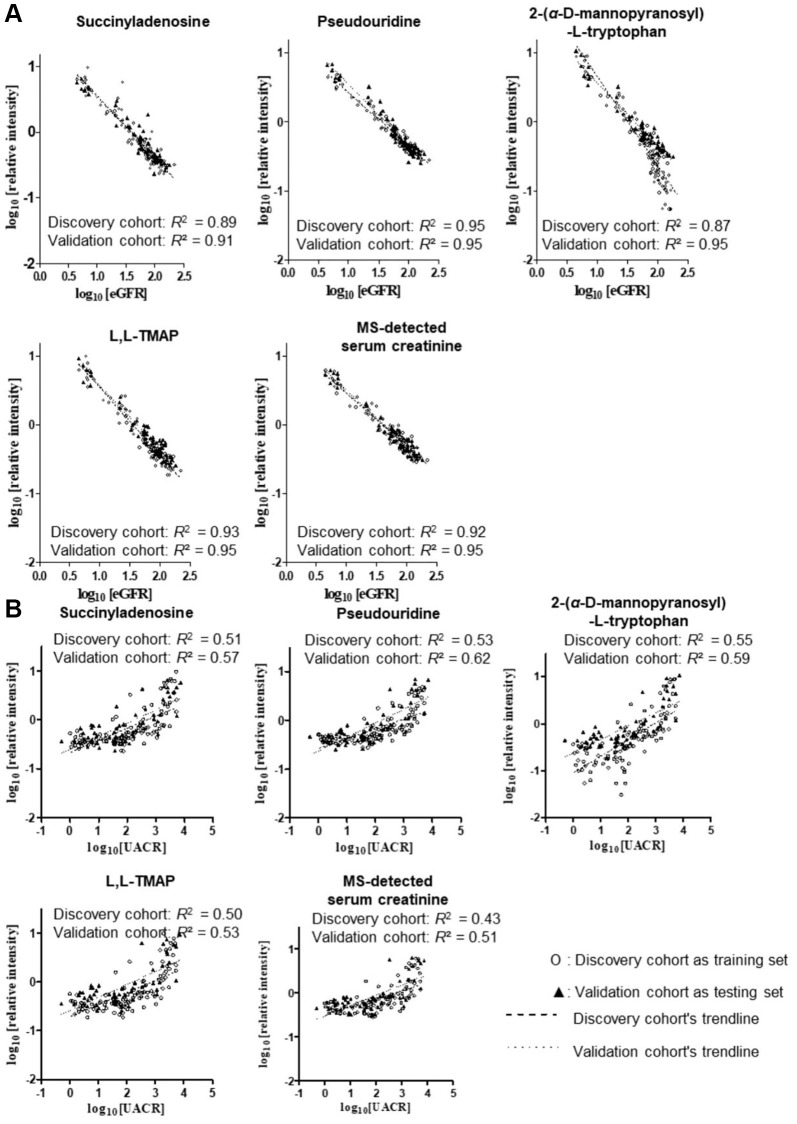
Linear regression analysis among CDBs, sCr, eGFR (**A**) and UACR (**B**) in all stages and T2D patients after log10 transformation, respectively. Four metabolites showed similar strong predictive power with MS-detected sCr as their R2 of the equations were all above 0.85. R2 of UACR prediction model were close to 0.5.

Receiver Operating Characteristic (ROC) curve analysis and Spearman’s coefficient coexpression analysis were used to evaluate the power of each metabolite as well as the combinations of every 2–9 compounds in DKD staging. The metabolites that closely associated (|r| ≥ 0.6) with eGFR in all participants were shown in Supplementary Figure 6. Strict rank coefficient cut-off values of 0.8 for Stage 0–4 (all participants) and Stage 1–4 (all patients), and 0.7 for Stage 1 and 2 (early-stage patients) were applied to identify biomarkers that closely correlated with eGFR progressive, four metabolites were screened out consist of SAdo ((M-H)^-^ = 382.1005 at 2.89 min), pseudouridne ((M-H)^-^ = 243.0622 at 0.93 min), ADT ((M-H)^-^ = 367.1497 at 2.21 min) and L,L-TMAP ((M+H)^+^ = 229.1546 at 1.06 min) (The regression plots among the four metabolites, eGFR and UACR see Figure 3). Since few studies have investigated the basic features of SAdo, the demonstration of its peak identification was plotted and calibrated (Supplementary Figures 7 and 8). To alleviate the bias induced by sex, age, SBP and UACR, partial correlation analysis (PCA) was performed among the four metabolites, serum cystatin C, MS-detected serum creatinine (MS-sCr) and log(eGFR). The PCA showed that log(MS-sCr), log(pseudouridine) and log(L,L-TMAP) were strikingly correlated with log(eGFR) (|r| > 0.9) in Stage 0–4 (Table 1). In addition, we evaluated the association between the four metabolites and kidney function related factors such as UACR, urinary *β*2- microglobulin, renal resistive index and the decrease of total BSA related renal volume, and found they were closely related as well (|r| > 0.5, see Table 1). The correlation among interested metabolites, total BSA-related renal volume and renal resistive index was calculated as well (Supplementary Table 7). In conclusion, our results suggested that the four metabolites are possibly involved in DKD progression and have potential to be utilized as candidate DKD biomarkers (CDBs).

**Table 1:** Spearman’s coefficient correlation *r* analysis among four metabolites, clinically measured sCr, cystatin C, eGFR and interested CKD risk factors in two cohorts.

**Stage ranges**	**Stage 0–4**		**Stage 1–4**		**Stage 1 and 2 (eGFR ≥60)**	
	**Discovery (*n* = 128)**	**Validation (*n* = 66)**	**Discovery (*n* = 108)**	**Validation (*n* = 56)**	**Discovery (*n* = 69)**	**Validation (*n* = 30)**
**Spearman rank correlation with eGFR**						
**Pseudouridine**	−0.896	−0.939	−0.939	−0.953	−0.789	−0.792
**ADT**	−0.875	−0.928	−0.918	−0.935	−0.733	−0.847
**MS-detected creatinine**	−0.869	−0.912	−0.900	−0.916	−0.712	−0.739
**L,L-TMAP**	−0.866	−0.901	−0.921	−0.922	−0.734	−0.739
**Succinyladenosine**	−0.855	−0.926	−0.899	−0.932	−0.706	−0.818
**Serum cystatin C**	−	−0.913	−	−0.940	−	−0.802
**Partial correlation of metabolites with log (eGFR) after controlling sex, age, SBP and log (UACR)**						
**log (MS-detected creatinine)**	−0.957	−0.975	−0.962	−0.976	−0.850	−0.847
**log (Pseudouridine)**	−0.941	−0.949	−0.947	−0.952	−0.739	−0.709
**log (L,L-TMAP)**	−0.924	−0.950	−0.930	−0.953	−0.685	−0.698
**log (Succinyladenosine)**	−0.867	−0.898	−0.874	−0.898	−0.533	−0.571
**log (2-(α-D-Mannopyranosyl)-L-tryptophan)**	−0.823	−0.956	−0.844	−0.958	−0.701	−0.755
**log (Serum cystatin C)**	−	−0.949	−	−0.953	−	−0.831
**CKD risk factors (Stage 1–4)**	**UACR**		**Urinary *β*2-microglobulin**		**Total BSV-related renal volume**	**Renal resistive index**
	**Discovery (*n* = 108)**	**Validation (*n* = 56)**	**Discovery (*n* = 108)**	**Validation (*n* = 54)**	**Validation (*n* = 48)**	**Validation (*n* = 48)**
**2-(*α*-D-Mannopyranosyl)-L-tryptophan**	0.801	0.746	0.664	0.731	−0.604	0.586
**Succinyladenosine**	0.795	0.690	0.635	0.780	−0.615	0.556
**Pseudouridine**	0.794	0.741	0.690	0.793	−0.599	0.588
**L,L-TMAP**	0.743	0.676	0.670	0.681	−0.596	0.517
**MS-detected creatinine**	0.681	0.657	0.667	0.685	−0.473	0.459
**Serum cystatin C (mg/L)**	−	0.676	−	0.675	−0.614	0.544

### Evaluation of CDB’s capacity in staging DKD by random forest (RF)

To evaluate whether CDBs can be applied for staging DKD, RF was employed to assess the classification power of the four CDBs as RF is a powerful supervised classification technique for decision making via building large numbers of decision tree models and merging all predictions from these trees to get an accurate and unprogressed prediction [12]. It exhibited that CDBs can specifically differentiate Stage 1a patients from Stage 1b-4, Stage 1a from Stages 1b-2, Stage 1b from Stage 2, and Stages 1a-1b from Stages 2-4 (average AUC > 0.700, Table 2). Among them, pseudouridine and SAdo achieve better performance than MS-sCr in all the staging process (Supplementary Table 8). Multiple combinations of the 4 metabolites and MS-sCr were generated and used for assessing their ability for DKD stratification. Any one of the four candidate DKD biomarkers combined with MS-sCr can gain higher AUC than MS-sCr alone (Table 2). Among these models, the No. 7 model (MS-sCr + pseudouridine + L,L-TMAP) ranks the best in phasing Stage 1a from the rest with average AUC > 0.9 (Table 2).

**Table 2:** List of mean AUC values for evaluating the predictive power of MS-detected sCr and multiple-metabolite models for differentiating DKD stages in T2D patients using random forest classification in two cohorts.

**Classification**	**Cohort**	**Serum creatinine**	**Model 1**	**Model 2**	**Model 3**	**Model 4**	**Model 5**	**Model 6**	**Model 7**
eGFR ≥ 119 vs. eGFR < 119	Dis	0.85 ± 0.05	0.93 ± 0.03	0.91 ± 0.03	0.92 ± 0.05	0.94 ± 0.02	0.93 ± 0.02	0.94 ± 0.02	0.95 ± 0.02
	Val	0.88 ± 0.08	0.93 ± 0.03	0.94 ± 0.04	0.94 ± 0.03	0.92 ± 0.03	0.92 ± 0.03	0.94 ± 0.03	0.93 ± 0.03
eGFR ≥ 119 vs. eGFR = 60–118	Dis	0.75 ± 0.08	0.87 ± 0.05	0.84 ± 0.06	0.88 ± 0.07	0.91 ± 0.04	0.89 ± 0.04	0.89 ± 0.05	0.91 ± 0.04
	Val	0.76 ± 0.10	0.85 ± 0.06	0.87 ± 0.07	0.87 ± 0.07	0.83 ± 0.09	0.81 ± 0.09	0.85 ± 0.07	0.89 ± 0.06
eGFR = 90–118 vs. eGFR = 60–89	Dis	0.68 ± 0.09	0.81 ± 0.06	0.79 ± 0.07	0.80 ± 0.07	0.77 ± 0.06	0.83 ± 0.05	0.82 ± 0.06	0.81 ± 0.06
	Val	0.60 ± 0.17	0.97 ± 0.06	0.86 ± 0.08	0.99 ± 0.03	0.97 ± 0.06	0.96 ± 0.05	0.98 ± 0.03	0.95 ± 0.07
eGFR ≥ 90 vs. eGFR < 90	Dis	0.92 ± 0.03	0.96 ± 0.02	0.93 ± 0.02	0.94 ± 0.02	0.94 ± 0.02	0.96 ± 0.02	0.96 ± 0.02	0.95 ± 0.02
	Val	0.93 ± 0.05	0.99 ± <0.01	0.98 ± 0.02	0.97 ± 0.02	1.00 ± <0.01	1.00 ± 0.01	1.00 ± <0.01	0.99 ± 0.01
eGFR ≥ 60 vs. eGFR < 60	Dis	0.95 ± 0.03	0.99 ± 0.01	0.97 ± 0.01	0.98 ± 0.01	0.99 ± 0.01	0.99 ± 0.01	0.98 ± 0.01	0.99 ± 0.01
	Val	0.931± 0.04	0.96 ± 0.03	0.96 ± 0.03	0.94 ± 0.03	0.96 ± 0.03	0.96 ± 0.03	0.96 ± 0.03	0.97 ± 0.02
eGFR ≥ 30 vs. eGFR < 30	Dis	0.99 ± <0.01	0.99 ± 0.01	1.00 ± <0.01	1.00 ± <0.01	1.00 ± 0.01	1.00 ± <0.01	1.00 ± <0.01	1.00 ± <0.01
	Val	0.97 ± 0.08	0.99 ± 0.04	1.00 ± 0.02	0.99 ± 0.03	0.99 ± 0.04	0.99 ± 0.04	0.99 ± 0.03	0.98 ± 0.06

Data were expressed as mean ± SD. Abbreviations: Dis: discovery cohort; Val: validation cohort. Model 1: MS-detected sCr + pseudouridine; Model 2: MS-detected sCr + Sado; Model 3: pseudouridine + SAdo; Model 4: pseudouridine + L,L-TMAP; Model 5: Model 1 + ADT; Model 6: Model 1 + Sado; Model 7: Model 1 + L,L-TMAP.

### Comparison of CDBs between male and female patients

**Figure 4:**
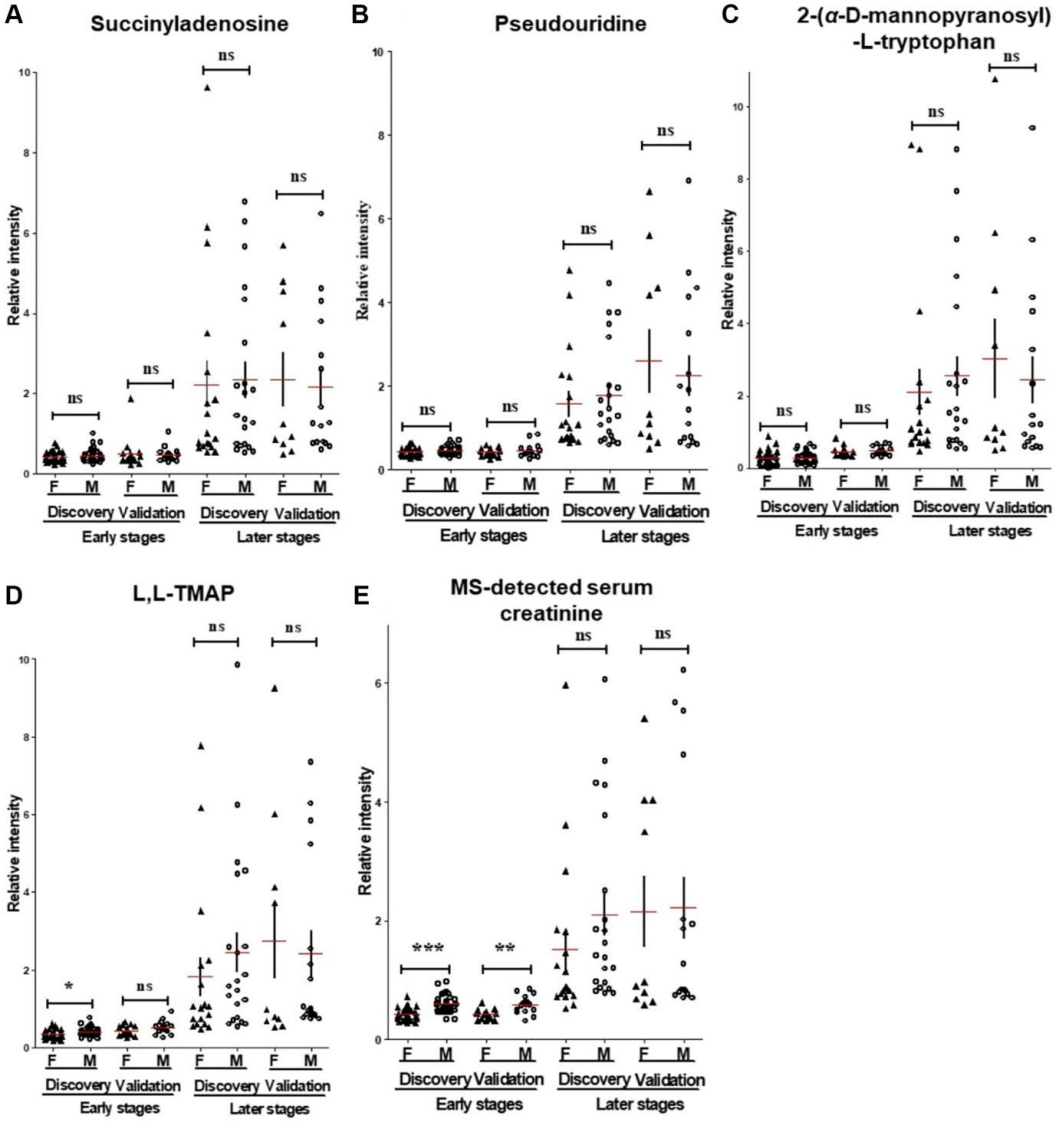
**The MS-detected CDBs.** (**A**–**D**) and sCr (**E**) were evaluated and compared between male and female participants at the early and later stages’ patients. Unlike with sCr, three of the four CDBs include SAdo, pseudouridine and ADT displayed non-significant differences between male and female patients at early or later stages. F, women; M, men. *p* value was calculated by Student’s *t*-test and Mann-Whitney U according to the data normality. ^*^p < 0.05, ^**^p < 0.01, ^***^p < 0.001, respectively. Horizontal and error bars in the scatter plots represent mean ± SEM.

Increasing evidence suggested that sexual difference is a significant factor related with DKD progression, which leads to a complex personalized approach for DKD diagnosis and treatment in clinical practice [13, 14]. To investigate the association between the four CDBs and sexual difference, we compared their levels between male and female patients. For SAdo, pseudouridine and ADT at early and later stages, no significant differences was observed (Figure 4). Multiple linear regression analysis which included sex as a covariate showed that CDBs show insignificant sex dependence with eGFR, suggesting that CDBs can be utilized in both male and female patients (Supplementary Table 9).

### Predict eGFR using CDBs signatures

**Figure 5:**
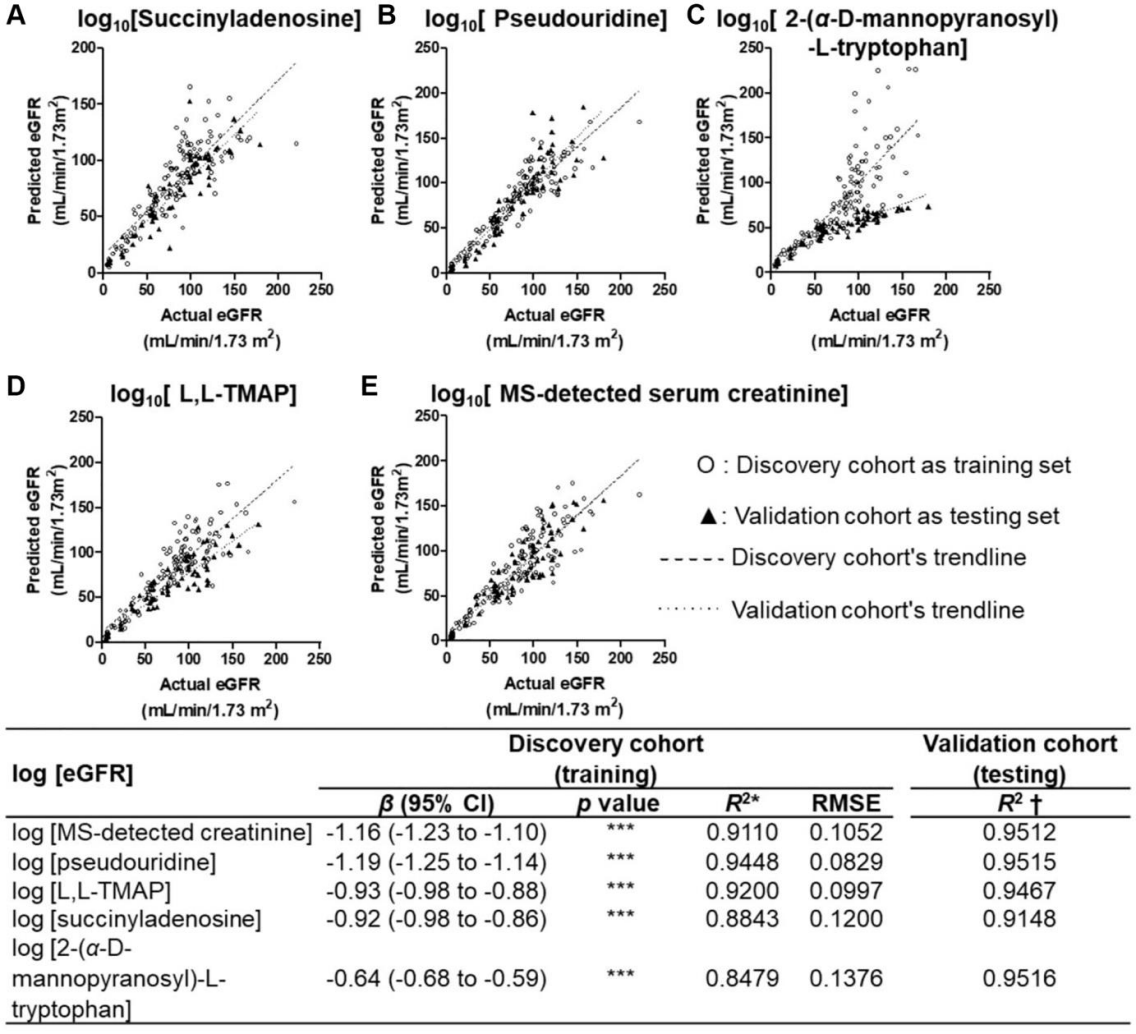
**Univariate linear regression plots of BeGFR against MDRD eGFR using the four CDBs.** (**A**–**D**) and MS-detected sCr (**E**) for all participants at Stages 0–4 after log_10_ transformation. Univariate linear regression analysis of each selected metabolites with all participants’ log(MDRD eGFR) resulted in a high linear relationship (training R^2^ = 0.85–0.94, root mean square errors (RMSEs) = 0.08–0.13; predictive R^2^ = 0.91–0.95), which was similar with that of MS-detected sCr (training R^2^ = 0.95, RMSE = 0.11; predictive R^2^ = 0.95). ^***^p < 0.001 *β*, unstandardized coefficient of linear regression. ^*^R^2^ was calculated based on the log(BeGFR) against log (eGFR) using the equation of the model and data of the discovery cohort. ^†^*R*^2^ was measured based on that using the equation of the model of the discovery cohort and data of the validation cohort.

We hypothesized that the use of a combinations of multiple biomarkers may be more sensitive and specific than sCr in evaluating the kidney function of diabetic patients. To test the potential of CDBs in predicting DKD, non-parametric methods include univariate linear regression (ULR) and multivariate linear regression (MvLR) were applied to calculate the association among eGFR, UACR, creatinine and four CDBs. The ULR analysis using Stage 0–4 data found a high linear relationship between each CDB and log(eGFR) (training *R*^2^ = 0.87–0.95, root mean square errors (RMSEs) = 0.08–0.13; predictive *R*^2^ = 0.91–0.95) which was very close to MS-detected sCr (training *R*^2^ = 0.95, RMSE = 0.11; predictive *R*^2^ = 0.95) (Figure 5), suggested that CDBs are good covariates to be applied for eGFR prediction. A stepwise MvLR analysis was performed to test the effects of four CDBs and covariates (sex, age, SBP and UACR) in calculating CDB-predicted eGFR (BeGFR) using data from Stage 0–4 and Stage 0–2, respectively. Among all the individuals (Stage 0–4), MS-sCr, pseudouridine, L,L-TMAP and sex are the most significant variables, and were therefore considered as confounding covariants for BeGFR estimation. The predictive outcome *R*^2^ was optimized from 0.971 (log(MS-detected sCr) and sex as covariates) to 0.987 (Table 3).

**Table 3:** Multivariate linear regression analyses of biomarkers with log (eGFR) trained with discovery set and tested with validation set among all participants.

**Prediction of log (BeGFR)**	**Stages 0–4**			**Stages 0-2**		
	**Discovery (training)**		**Validation (testing)**	**Discovery (training)**		**Validation (testing)**
	*R* ^2*^	**RMSE**	***R* ^2†^**	*R* ^2*^	**RMSE**	***R* ^2†^**
	0.9562	0.0740	0.9714	0.6802	0.0606	0.7050
	***β* (95% CI)**	***p* value**		***β* (95% CI)**	***p* value**	
log (MS-detected creatinine)	**–1.23 (–1.28 to –1.19)**	^***^		**–0.93 (–1.06 to –0.79)**	^***^	
sex	**0.15 (0.13 to 0.18)**	^***^		**0.12 (0.09 to 0.16)**	^***^	
**All biomarkers and common covariates**	*R* ^2*^	**RMSE**	***R* ^2†^**	*R* ^2*^	**RMSE**	***R* ^2†^**
	0.9403	0.0514	0.9855	0.7949	0.0503	0.8348
	***β* (95% CI)**	***p* value**		***β* (95% CI)**	***p* value**	
log (MS-detected creatinine)	–0.71 (–0.86 to –0.56)	^***^		–0.71 (–0.87 to –0.54)	^***^	
log (pseudouridine)	–0.35 (–0.58 to –0.13)	^**^		–0.23 (–0.50 to 0.07)	ns	
log (L,L-TMAP)	–0.14 (–0.28 to –0.01)	^*^		–0.06 (–0.22 to 0.10)	ns	
log (succinyladenosine)	–0.01 (–0.11 to 0.09)	ns		0.01 (–0.13 to 0.14)	ns	
log (2-(*α*-D-mannopyranosyl)-L-tryptophan)	–0.02 (–0.09 to 0.04)	ns		–0.07 (–0.15 to 0.003)	ns	
sex	0.11 (0.08 to 0.13)	^***^		0.10 (0.07 to 0.13)	^***^	
age	–0.0009 (–0.003 to 0.001)	Ns		–0.0005 (–0.003 to 0.002)	ns	
SBP	0.0003 (–0.0003 to 0.0009)	ns		0.0003 (–0.0003 to 0.0009)	ns	
BMI	0.002 (–0.001 to 0.006)	ns		0.0026 (–0.001 to 0.01)	ns	
log (UACR)	0.006 (–0.009 to 0.02)	ns		0.0049 (–0.01 to 0.02)	ns	
**The best model by stepwise method using variables with *p* < 0.05**	*R* ^2*^	**RMSE**	***R* ^2†^**	*R*^2^*	**RMSE**	***R* ^2†^**
	0.9754	0.0514	0.9870	0.7733	0.0513	0.8200
	***β* (95% CI)**	***p* value**		***β* (95% CI)**	***p* value**	
log (MS-detected creatinine)	–0.64 (–0.77 to –0.51)	^***^		–0.68 (–0.82 to –0.53)	^***^	
log (L,L-TMAP)	–0.13 (–0.25 to –0.01)	^*^		N/A	N/A	
log (pseudouridine)	–0.44 (–0.59 to –0.30)	^***^		–0.46 (–0.62 to **−**0.31)	^***^	
sex	0.10 (0.08 to 0.12)	^***^		0.10 (0.07 to 0.13)	^***^	

*β*, unstandardized coefficient of linear regression. Sex, female = 1 and male = 2. RMSE, root mean square error. ^*^R^2^ was based on the predicted log (eGFR) against actual log (eGFR) using the equation of the model and data of discovery set. ^†^*R*^2^ was based on that using the equation of the model of discovery set and data of validation set. ^*^p < 0.05, ^**^p < 0.01, ^***^p < 0.001, Abbreviation: ns: indicates no significance.

Renal function of early-stage DKD (Stage 1 and 2) is reversible and manageable [15, 16]; however, most DKD patients are asymptomatic and indolent [17, 18]. Considered that early detection is of great vital for lifetime benefits for DKD patients, we specifically tested the predictive potency of CDBs and eGFR in early-stage participants. Surprisingly, pseudouridine and L,L-TMAP can enhance the predictive power of MS-detected sCr at early stages’ patients and healthy participants (Stage 0–2), predictive *R*^2^ in the validation datasets was significantly improved from 0.70 (log(MS-detected creatinine) and sex as covariates) to 0.82 (Table 3), demonstrating that the two CDBs are potential biomarkers for the early detection of DKD. The best model for BeGFR estimation at the early stage (training *R*^2^ = 0.7733, RMSEs = 0.0513) is: log(BeGFR) = −0.675 log(MS-detected sCr) −0.467 log(pseudourdine) + 0.101 (if male) + 1.559. Our results indicated that the combination of multiple biomarkers achieves better performance than standard sCr.

### Follow-up study and prognostic assessment

**Figure 6:**
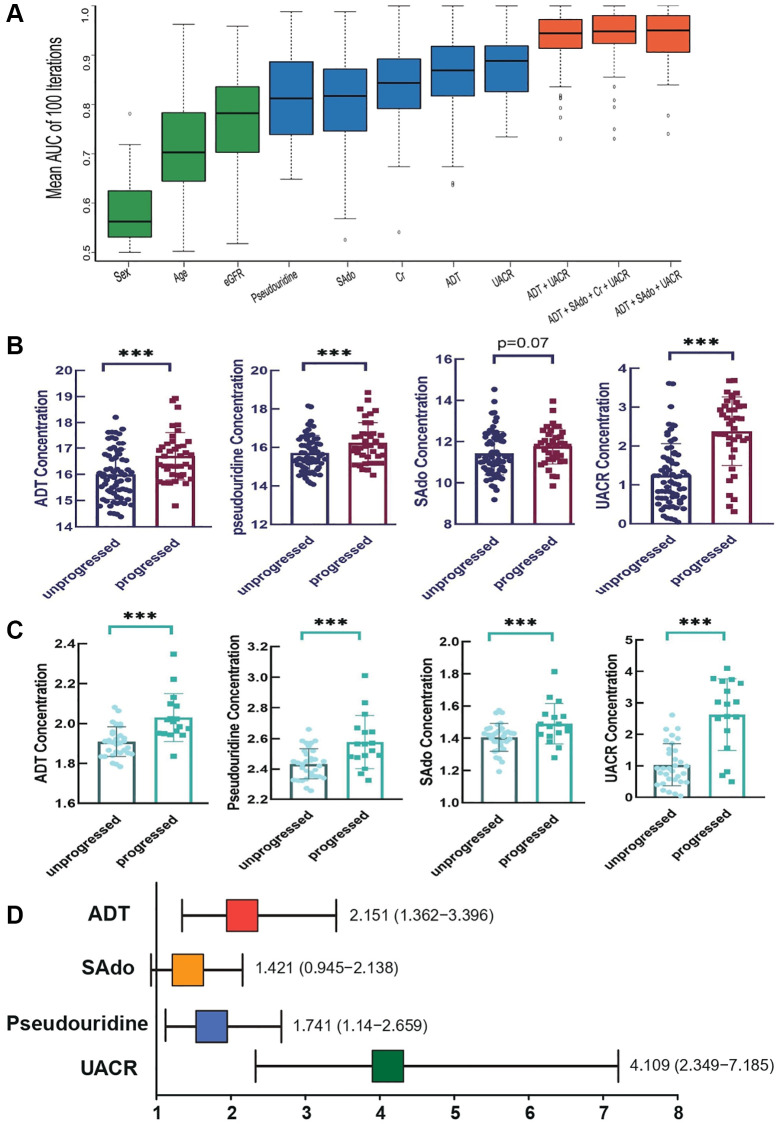
**Evaluation of the prognostic performance of CDBs in follow-up cohort.** (**A**) The distribution of AUC values using single and combinations of variate(s) in follow-up patients. With stratified random sampling and random forest, AUC of distinction between and progressed patients were calculated 100 times with single and multiple variables. Results of AUC average and standard deviation indicated that ADT_SAdo_UACR (AUC average: 0.9502; CI: 0.9062–0.9805) manifested the best prediction, followed with UACR_ADT_SAdo_sCr (AUC average: 0.9482; CI:0.9248-0.9805) and ADT_UACR (AUC average: 0.9443; CI: 0.9141–0.9727). (**B**) Comparisons of the three CDB levels and UACR between “progressed” and “unprogressed” groups in all the follow-up individuals. (**C**) In stage 2 patients, levels of three CDBs and UCAR were remarkably different in “progressed” vs. “unprogressed”, ^**^p < 0.01 and ^***^p < 0.001, respectively. (**D**) Risk sores of three CDBs and UCAR in DKD progression by logistic regression analysis.

Due to the limitations for purchasing the commercial standards of L-L-TMAP, only three CDBs include ADT, SAdo and pseudouridine and some clinical indexes were measured in the follow-up cohort. The association were assessed between 7 variates (sex, age, eGFR, sCr, ADT, SAdo and pseudouridine) as well as their combinations with DKD progression. The first-time collected serum samples from 106 subjects of Stage 2 and 3 DKD patients were examined to determine the concentration of the metabolites. In Stage 2 unprogressed and Stage 2 progressed groups, for each single variate, ADT ranks the top prognostic power (average AUC = 0.9184) than sCr alone (average AUC = 0.9133). Surprisingly, the combinations of “UACR + ADT + sCr” and “UACR + ADT + age + sex” were extremely associated with the future progression of DKD (AUC ranges from 0.9592 to 1) (Figure 6A, Supplementary Table 10). The same method was applied in Stage 3 unprogressed and Stage 3 progressed patients. Three single variates, including UACR, pseudouridine and ADT, gained the top 3 strongest association with DKD progression (average AUC values = 0.8889, 0.8302 and 0.8117, respectively). For the Stage 3 patients, either of the four metabolites (ADT, sCr, SAdo and pseudouridine) combined with UACR can optimize average AUC value ≥ 0.9012; among the combinations, “UACR + ADT + pseudouridine + SAdo + Sex” and “UACR + pseudouridine + sCr” achieve the best performance with an average AUC = 0.929 (Figure 6A, Supplementary Table 11). Ignoring the initial DKD phasing status, “UACR + ADT + SAdo” is the best combination for DKD prognostic assessment (average AUC = 0.9502).

In general clinical practice, eGFR was employed as a popular reporter for grading diseases as its levels reflect the status of renal function decline; while UACR was mostly used as the predictive biomarker for disease’s progression [7]. Hence, we only compared the changes of UACR and CDBs between progressed and unprogressed groups in all follow-up individuals, it showed that the levels of ADT, pseudouridine and AUCR were significant different between the two categories while SAdo showed slightly but non-significant differences (Figure 6B). Among stage 2 incidents, concentrations of ADT, pseudouridine, Sado and AUCR were remarkably different in progressed patients comparing with the unprogressed (Figure 6C). Further logistic regression analysis indicated that ADT and pseudouridine are risk factors for DKD development (Figure 6D). With every single increase of standard deviation (SD) of ADT, the risk of DKD progression is enhanced for 2.151 folds; for pseudouridine, the risk scores are 1.741 folds (Figure 6D). To better evaluate the associations between the levels of risk factors and the DKD progressing, we used the duration of time for progressing to later stage in follow-up individuals for survival curve analysis. It showed that patients with higher levels of either pseudouridine or ADT had significant less survival probabilities (*p* < 0.05), which is similar to UACR (Supplementary Figure 9). Taken together, our cross-sectional study indicated that abnormal metabolism is involved in DKD progression and our follow-up study validated the predictive power of CDBs in DKD development.

## DISCUSSION

A precise assessment of renal function in the clinical settings, would be instructive for management of DKD, such as for the prediction and intervention of the disease progression, CKD staging, for assessing the need for dialysis therapy, and adjustment of nephrotoxic agents dosage for patients [19]. In the past decades, eGFR has been applied as the best overall measurement of kidney function in medical practice; however, it also has some limits on accuracy and reliability [20, 21]. To overcome the limitations, over 70 equations have been developed for estimating eGFR. We applied Modification of Diet in Renal Disease (MDRD) formula to calculate eGFR for DKD classification; nevertheless, other methods include (CKD-EPI)_creatinine_ [22], CKD-EPI_cystatin C_ [23] and CKD-EPI_creatinine–cystatin C_ [23] equations were calculated as well. CKD-EPI_creatinine_ eGFR was strongly correlated with MDRD eGFR (Pearson’s r = 0.9523 and 0.9729 in discovery and validation sets, respectively). CKD-EPI_cystatin C_ and CKD-EPI_creatinine–cystatin C_ eGFR also show high correlation with MDRD eGFR with Pearson’s r = 0.9468 in discovery sets and 0.9681 in validation sets (Supplementary Table 12).

Machine learning approaches such as RF, decision tree, logistic regression and XG Boost have greatly advanced the development of biomedical science especially for the prognostic prediction of human diseases. RF was applied to assess the covariates associated with DKD development as it is one of the most efficient and widely used algorithms that leverages a collection of decision trees for making decisions; on the other hand, we used logistic regression to the estimates the risk probability of DKD progression using CDB levels considering this algorithm is useful to obtain odds ratio in the presence of more than one explanatory variable [12]. Metabolomics analysis can be classified into two categories, namely the non-targeted and the targeted approach. Considering that the first one is an unbiased metabolomic analysis that can discover new biomarkers [24], the non-targeted approach has been adopted to gain a more comprehensively and systematically knowledge of the progressive DKD. All the DKD patients suffer from dysregulated metabolic milieu including hyperglycemia and insulin resistance that lead to renal functions being impaired. In the design of this study, subjects with various degree of renal function impairments are recruited and these would be one of the major variations among the subjects. However, it is unavoidable that these subjects would also have different status of hyperglycemia and insulin resistance although most of them have a longer history of diabetes. Thus, although this cohort may not be a good one for metabolomics study of diabetes, but metabolties related to the progression of diabetes may also revealed in this study. The endogenous metabolite, 1,5-anhydro-D-glucitol (1,5-AG), is an example of these which correlate with eGFR, but show a stronger correlation with serum hemoglobin A1c (HbA1c) and fasting blood-glucose (FBG) which are important clinical markers for hyperglycemia. Apart from that, Liu et al. found the catabolism of amino acids in plasma of individuals of DKD with T2D was accelerated [25]. The targeted metabolic nuclear magnetic resonance (NMR) spectroscopy of European T2D patients revealed that the amino acids glycine, phenylalanine, the energy metabolites citrate and glycerol were negatively associated with eGFR, while alanine, valine and pyruvate depicted opposite association in diabetics (positive) and non-diabetics (negative) [26]. Accumulating evidence suggested that aromatic amino acids (phenylalanine) and branched-chain amino acids (BCAAs) such as leucine and valine were associated with an increased risk of developing T2D [27, 28]. Our results showed consistent findings that amino acids were significantly changed among different stages, especially leucine, valine and phenylalanine (Supplementary Tables 5 and 6). Although the markers correlated with the progression of diabetes is not the focus of this study, but our data clearly supported they are being affected during the development of diabetes. Metabolites related to both hyperglycemia and renal functions are being identified in the analysis also demonstrated the non-targeted metabolomics analysis performed in this study is of very good quality and the data is capable of revealing various differences in the host metabolism.

Since DKD is asymptomatic until later stages, its early detection is of great significance to provide an opportunity for preventing or delaying its progression and decreasing morbidity and mortality. Small molecules are extensively metabolized by kidney and the impaired renal function can lead to the changes of serum metabolites, hence, they may be used to estimate filtration (e.g., the established marker creatinine) or precede and potentially contribute to the development of kidney diseases [29]. In this study, pseudouridine, L-L-TMAP, ADT and SAdo were identified as the candidate biomarkers for optimizing DKD stratification and eGFR prediction. Pseudouridine has been identified as a non-traditional kidney function marker in previous study as it shows significant correlation with eGFR in general population [30] while TMAP has shown better performance than creatinine in accurately identifying patients with a single kidney [31]. Yonemura et al. revealed that the concentration of serum ADT is a more reliable diagnostic marker than that of serum creatinine as a measure of normal renal function [32]. Our study reconfirmed the potential of pseudouridine, L-TMAP, ADT in measuring renal function; in addition, to the best of our knowledge, we reported that SAdo is a new candidate biomarker and can be utilized to predict the progression of early stages’ DKD for the first time. Interestingly, the concentration of the four serum biomarkers were not only strongly correlated with eGFR but also associated with non-GFR renal injury indicators (nGRI) including UACR, urinary *β*2-microglobulin, RRI and kidney sizes. These four nGRI were usually served as indicators of albuminuria [19, 33], renal proximal tubular reabsorption dysfunction [34], renal arterial damage and resistance [35], and kidney hyperfiltration and degeneration [36], respectively. Our results suggested the four metabolites are indicators of glomerular filtration dysfunction and kidney pathophysiology damages as well. In contrast with serum creatinine that can be easily affected by sex and muscle metabolism, we found SAdo, ADT and pseudouridine are sexual independent.

Prognostic markers play important role in DKD patients stratification, treatment choice and future outcome assessment. Our follow-up results of Stage-2 and Stage 3 patients offered further evidence to the hypothesis that the four biomarkers are prognostic markers for disease progression (both renal function decline and UACR increment) in patients with early DKD. Similar results about pseudouridine and ADT in disease progression were acquired in our study, comparing with other DKD follow up cohort [37]. For the first time, our follow-up study gave a clinical evidence-based proof for succinyladenosine as a DKD prognostic marker and turned out to have good prognostic value, especially at early stage. These markers would facilitate both doctors and patients on their treatment selection and aid in clinical practice.

## CONCLUSION

For the first time, we demonstrated that SAdo is a new potential biomarker for eGFR estimation and DKD prognostic assessment. Consistent with previous studies, the predictive potential of pseudouridine, L-TMAP, ADT in measuring renal function was further confirmed in our cohorts. These four serum biomarkers were not only strongly correlated with eGFR but also closely associated with non-GFR renal injury indicators. Unlike serum creatinine with noticeable sexual difference, SAdo, ADT and pseudouridine are sexual independent. Our follow-up study validated the prognostic power of the above biomarkers for both renal function decline and UACR increment in early DKD patients. This study provided comprehensive insights into the signatures of metabolites in Chinese DKD patients and identified four candidate biomarkers for better monitoring of DKD.

## MATERIALS AND METHODS

### Study design and participants

The 194 serum samples were collected spanning five DKD stages. As these two groups of samples were subjected to untargeted metabolites analysis at different times, we analyzed them separately to remove batch effect and separately referred them as the discovery and validation sets (Figure 1). Participants were required to cease taking unnecessary medications and fasted for 8 hours before serum collection. We examined all participants’ clinical parameters, reviewed and recorded their medical history, medical complications and dietary habits (see Supplementary Table 1). Based on the ADA and KDIGO criteria [33, 38] for diagnosis of diabetes, participants were firstly classified into healthy group (stage 0) and diabetic group. The healthy and diabetic groups are age and sex matched (Supplementary Table 2). We followed the MDRD formula: “eGFR (mL/min/1.73 m^2^) = 186 × (serum creatinine)^−1.154^ × (age in years)^−0.203^ × 0.742 (if female) × 1.210 (if African American)” to calculate the eGFR values [39]. According to the eGFR levels, diabetic individuals were further stratified to five types, which are stage 1a-b and stage 2 to 4 (criteria see Supplementary Materials and Methods); the healthy group participants were regarded as stage 0. The renal resistive index (RRI) was calculated as (peak systolic velocity - end diastolic velocity)/peak systolic velocity derived from the kidney doppler ultrasonography. Additional methods for measuring eGFR values were shown in Supplementary Table 12 [22]. All the DKD-related clinical parameters were measured in The Fourth Clinical Medical College of Guangzhou University of Chinese Medicine (Shenzhen Traditional Chinese Medicine Hospital) followed by the standard procedures.

### Measurements of serum untargeted metabolites

Serum samples and an equal volume of quality control (QC) samples (Supplementary Table 3) were deproteinated with cold methanol that contains internal standards. Ultra-Performance Liquid Chromatography-Orbitrap-Mass Spectrometry (UPLC-Orbitrap-MS) analysis was conducted on a Waters ACQUITY UPLC system coupled to a Thermo Scientific Orbitrap Fusion Lumos Tribrid mass spectrometer for mass spectrometry (MS) analysis. For detailed steps of UPLC-Orbitrap-MS, please see the Supplementary Materials and Methods.

### Untargeted metabolites analysis

UPLC-Orbitrap-MS data from the discovery and validation cohorts were analyzed separately. Data were firstly processed by Progenesis QI 2.3 software (Nonlinear Dynamics, Waters, Milford, MA, USA) for peak detection and alignment, and then subjected to Matlab (MathWorks, Natick, MA, USA) for exclusion of unreliable features with missing rates >40% and missing value imputation [40, 41]. We performed baseline correction via cubic spline interpolation to align the baseline levels of data obtained at different times [42] (Supplementary Figures 10–84). Unstable signals with a coefficient of variation (CV%) >30% across the QC samples were filtered out. Compounds were identified upon matching their mass to charge ratio (m/z) and mass fragmentation patterns against available reference standards and Human Metabolome Database (hmdb.ca) (Supplementary Table 13) [43]. To gain a unique view of DKD, only metabolites that repeat the same mass fragmentation pattern, retention time and show the same trend of significant statistical differences in both discovery and validation sets were kept for further investigation.

### Evaluation of CDBs’ performance on DKD disease stage classification using random forest

Random forest (RF) algorithm is applicable for evaluating the performance of metabolites on differentiating disease stages [44]. We used the RF package scikit-learn [45] that was implemented by Python (version 3.8) to evaluate the classification power of candidate DKD biomarkers (n_estimators = 10–100). Area under curve (AUC) using 1–9 metabolite models was determined by RF, respectively. To avoid overfitting, samples in each cohort were randomly and evenly divided into training and testing sets for the establishment and performance evaluation of the models. This step was repeated 100 times to obtain the mean AUC values using the testing set of the two cohorts.

### Significant altered metabolites analysis

One-way ANOVA followed by Fisher’s LSD post-hoc test was used to identify significant altered metabolites (SAMs). Metabolites with *p*-value < 0.05 and false discovery rate (FDR) < 0.1 among any two of the stages were regarded as SAMs.

### Metabonomic networks analysis

Networks of metabolites (Pubmed ID listed in Supplementary Table 13) were generated through MetaMapp [46] with default parameters. CytoScape [47] was used to visualize the networks. Their metabolic pathway output was generated on the basis of their KEGG reaction pairs while their chemical and structural relationships were constructed by their Tanimoto similarity.

### Linear regression of CDBs for log(eGRF) estimation

Using CDBs and covariates such as sex, age, SBP and UACR, univariate linear regression (ULR) and multivariate linear regression (MvLR) were utilized to determine log(eGFR). Discovery group was used as the training set. The unstandardized regression coefficients (*β*) of the training set’s model were applied to generate equations for log(eGFR) prediction in testing set -- the validation cohort. Variables that contributed to the model with *p* < 0.05 were selected by stepwise linear regression analysis for the best model in favor of a simpler model.

### Follow-up study and targeted metabolites prognostic assessment

To identify the progression of DKD, we recruited an extra cohort of 106 patients at stage 2 and 3 for an average 3.9 years of follow-up study. Patients’ serums were collected since the starting point and the sCr, Bun and eGFR were measured every 3 months. The serum samples collected at the first time were subjected to targeted metabolomic analysis for the quantification of ADT, SAdo and pseudouridine by an ultra-high performance liquid chromatography (Shimadzu, Kyoto, Japan) coupled with the AB SCIEX Q-Trap 5500 triple quadrupole mass spectrometer (AB SCIEX, Toronto, Canada). During the follow-up period, patients who remained at their original stage were regarded as the “unprogressed” group; those who progressed to later stages accompanying with a 25% drop in eGFR [48] were “progressed” group. Random forest algorithm [20] were used to evaluate covariates that associated with DKD progression. The prognostic power of biomarkers during DKD stage progression was assessed via performing 100 iterations for each variate. Logistic regression analysis was used to evaluate the risk scores of interested metabolites by SPSS. The average AUC values were calculated and compared among all the variates. Detailed methods of targeted metabolite analysis with UPLC-QQQ-MS/MS and random forest analysis can be found in the Supplementary Materials and Methods (Supplementary Tables 14–16).

### Data availability

The raw data were submitted to National Genomics Data Center database under the bioproject PRJCA013833 (https://ngdc.cncb.ac.cn/bioproject/browse/PRJCA013833). All the data analyzed during the current study are available from the corresponding author on reasonable request.

## Supplementary Materials

Supplementary Materials and Methods

Supplementary Figures

Supplementary Tables 1, 3, 4, 7-9, 11, 12 and 14-16

Supplementary Tables 2, 5, 6, 10 and 13
