# Supplementary material for: Insights into serum metabolic biomarkers for early detection of incident diabetic kidney disease in Chinese patients with type 2 diabetes by random forest
Source: Aging (Albany NY). 2024 Feb 12;16(4):3420–530. doi: 10.18632/aging.205542 (PMC10929832; doi:10.18632/aging.205542)
Supplement: Supplementary Materials and Methods [file aging-16-205542-s001.pdf]

SUPPLEMENTARY MATERIALS AND METHODS

Sample preparation and UPLC-Orbitrap-MS conditions

Recruitment of participants

At study enrollment stage, a designed baseline clinical examination of each participant and structured interview were performed by trained recruiters. The exclusion criteria were acute renal failure, rapidly increasing proteinuria or nephrotic syndrome, refractory hypertension, serious infections, signs or symptoms of other systemic disease, known renal tubular acidosis, pregnancy, type 1 diabetes, gestational diabetes, chronic liver disease, serious cardiovascular diseases, alcoholics or malignancy. For healthy control, they had not received any treatment like antibiotics, probiotics and hormone therapy in the past two months, did not have proteinuria or history of kidney disease and their oral glucose tolerance test and other related clinical test are in normal levels. All baseline clinical information is shown in Supplementary Tables 1 and 2.

Criteria of stages' classification for participants

Based on the MDRD eGFR values, participants were classified into five stages by following criteria: Stage 1 (eGFR ≥ 90 ml/min/1.73 m^2) which consists of Stage 1a (eGFR ≥ 120 mL/min/1.73 m^2) and Stage 1b (eGFR within 90–120 mL/min/1.73 m^2); Stage 2, 60–89 mL/min/1.73 m^2; Stage 3, 30–59 mL/min/1.73 m^2; Stage 4, < 30 mL/min/1.73 m^2 [1, 2].

Serum preparation

60 µL serum was deproteinated with 240 µL cold methanol containing 0.5 ppm L-tryptophan (indole-D_5, 98%, Cambridge Isotope Laboratories, Tewksbury, MA, USA) and 0.5 ppm cholic acid-2,2,4,4-D_4 and 50 ppm C19:1n9c. They were vortexed for 1 min and stood at −20°C overnight for complete deproteination. Then, they were centrifuged at 18700 × g for 20 min. 250 µL supernatant was collected and dried under nitrogen gas and stored at −80°C. The dried supernatant was reconstituted with initial UPLC gradient (5% acetonitrile in water), vortexed for 30 s and was centrifuged at 18700 × g for 20 min. The supernatant was transferred to a glass insert in an amber HPLC vial prior to UPLC-Orbitrap-MS analysis or UPLC-QQQ-MS/MS analysis.

Standard solution and quality control sample preparation

For targeted metabolites quantitation, standards of selected metabolites were purchased from Toronto Research Chemicals (North York, Toronto, Canada) and Sigma-Aldrich (St. Louis, MO, USA), which were used for preparation of standard solutions. Standard solutions were gradient diluted into ten levels, respectively (Supplementary Table 14). The gradient diluted standard solutions were mixed and then dried by TurboVap® blowdown evaporator (Biotage Sweden AB, Ystrad Mynach, United Kingdom) for later use. 20 µL aliquots from each sample of all groups were mixed and aliquoted as QC samples. QC samples were injected between every six-sample injections to monitor the stability of the instruments throughout the UPLC-Orbitrap-MS signal acquisition. The order of injection for all samples was randomized. Recovery rate of detected metabolites was calculated through parallel serum samples spiked with a known amount of each metabolite standard at three concentration levels. Recovery rate equation: ((Detected concentration − endogenous blank sample concentration) × 100%)⁄spiked concentration. (Supplementary Table 15).

UPLC condition

UPLC-Orbitrap-MS analysis

3 µL aliquot was injected into a Waters ACQUITY UPLC system. UPLC separation was performed on a Waters ACQUITY UPLC HSS T3 column (2.1 mm × 100 mm, 1.8 µm) with HSS T3 guard column (2.1 mm × 5 mm, 1.8 µm, Waters Corporation, Milford, MA, USA). The mobile phase consisted of combinations of A (0.1% formic acid in water, v/v) and B (0.1% formic acid in acetonitrile, v/v) at a flow rate of 0.3 mL/min with elution gradient as follows: 0–1.5 min, 5% B; 2 min, 35% B; 4 min, 50% B; 8 min, 55% B; 11–14 min, 95% B. A 3-min post-run time was set to fully equilibrate the column. Column and sample chamber temperature were 40°C and 4°C respectively.

UPLC-QQQ-MS/MS analysis

2 µL aliquot was injected into a SHIMADZU A30 UPLC system. Chromatographic separation was performed on the Luna Omega 1.6 µm Polar C18 reversed-phase column (Phenomenex, Torrance, CA, USA) with Polar C18 security guard column (2.1 mm, Phenomenex, Torrance, CA, USA). The mobile phase A (0.1% formic acid in ultrapure water, v/v) and mobile phase B (100% acetonitrile) were delivered at 0.3 ml/min. Gradient elution was as follows: 2–60% B at 0–3.2 min, 60% maintained at 3.21–3.5 min, 2% B at 3.51–5 min to equilibrate the column before a new injection. Column and sample chamber temperature were 40°C and 4°C respectively.

Mass spectrometry condition

UPLC-Orbitrap-MS analysis

Mass spectrometry analysis was conducted by a Thermo Scientific Orbitrap Fusion Lumos Tribrid

mass spectrometer equipped with a heated electrospray ionization (H-ESI) interface (Thermo Fisher Scientific, Waltham, MA, USA). The mass-spectrometric parameters were set as follows: spray voltage, 2300 V and 3500 V in ESI negative and positive ionization modes respectively; ion transfer tube and vaporizer temperature, 300°C. Nitrogen gas was used as the sheath gas and the aux gas with a flow rate of 25 and 10 L/min, respectively. The analyzer was operated in a data-dependent acquisition mode, with full MS scans of mass range at 90–1000 m/z with detection in the Orbitrap (120000 resolution) and with auto gain control targeted at 20000 count and a maximum injection time at 100 ms.

UPLC-QQQ-MS/MS analysis

Selected metabolites were detected under positive ion multiple reaction monitoring (MRM) mode. Turbo ion spray source was set at a source temperature of 500°C. Ion spray voltage was 5500 V, Ion Source Gas1 (GS1) and Ion Source Gas2 (GS2) had a flow of 50 psi, the curtain gas had a flow of 25 psi, the CAD gas setting was 'medium', and the declustering potential was optimized one by one according to the metabolite. Q1/Q3 mass and MRM conditions for each metabolite were listed in Supplementary Table 16.

Baseline correction

Batch correction was then performed by smoothing through QC samples in sequential injections using cubic splines, a very flexible smoother that can catch the variations of ion abundances caused by the systematic bias in instrumental responses, with a very wide range of curve shapes (e.g., linear, nonlinear) (van der Kloet et al. 2009) to ([3–5]). The penalty for smoothing spline was set to 0.01, which was found to be fitted well to the variations (Supplementary Figures 10–84). The ion abundance for metabolite i at k^{th} injection after batch correction ($x'_{k,i}$) then becomes

$$x'_{k,i} = C_{QC,i}, r_{k,i}, \text{ where } r_{k,i} = \frac{x_{k,i}}{f_{k,i}}$$

where $C_{QC,i}$ was the true concentration of metabolite i in QC samples, which served as a scaling factor to map the corrected ion abundance $r_{k,i}$ to the corrected raw ion abundance ($x'_{k,i}$). However, it is impossible to obtain the true concentration of any metabolite, thus median ion abundance of the metabolite in QC samples could be used as an estimation of $C_{QC,i}$. $x_{k,i}$ and $f_{k,i}$ are observed and fitted raw ion abundance in sample at k^{th} injection. Since results obtained from statistical analysis (e.g., Pearson correlation, Student's *t*-test) using $x'_{k,I}$ will be the same with those using $r_{k,i}$ as the two kinds of ion abundance only differ in a constant multiplier $C_{QC,i}$, $r_{k,i}$ was used in subsequent statistical analysis instead of the raw ion abundance.

Random forest of metabolite prediction on DKD stage progression in follow up cohort

AUC of variate(s) on prediction DKD stage progression were calculated by random forest (RF). Stratified random sampling was used between progressed and unprogressed group in follow up cohort. Samples were split into a training set (70% of sample size) for modelling and a testing set (the rest 30% samples) for prediction. To avoid overfitting, this stratified random sampling procedure was repeated 100 times and the AUC of testing set was calculated 100 times. Finally, the AUC average and standard deviation were executed to exhibit the performance prediction of metabolites on DKD stage progression.

Supplementary References

1. Levey AS, Coresh J, Balk E, Kausz AT, Levin A, Steffes MW, Hogg RJ, Perrone RD, Lau J, Eknoyan G, and National Kidney Foundation. National Kidney Foundation practice guidelines for chronic kidney disease: evaluation, classification, and stratification. Ann Intern Med. 2003; 139:137–47. https://doi.org/10.7326/0003-4819-139-2-200307150-00013 PMID:12859163

2. Inker LA, Schmid CH, Tighiouart H, Eckfeldt JH, Feldman HI, Greene T, Kusek JW, Manzi J, Van Lente F, Zhang YL, Coresh J, Levey AS, and CKD-EPI Investigators. Estimating glomerular filtration rate from serum creatinine and cystatin C. N Engl J Med. 2012; 367:20–9. https://doi.org/10.1056/NEJMoa1114248 PMID:22762315

3. van der Kloet FM, Bobeldijk I, Verheij ER, Jellema RH. Analytical error reduction using single point calibration for accurate and precise metabolomic phenotyping. J Proteome Res. 2009; 8:5132–41. https://doi.org/10.1021/pr900499r PMID:19754161

4. Armitage EG, Godzien J, Alonso-Herranz V, López-Gonzálvez Á, Barbas C. Missing value imputation strategies for metabolomics data. Electrophoresis. 2015; 36:3050–60. https://doi.org/10.1002/elps.201500352 PMID:26376450

5. Wei R, Wang J, Su M, Jia E, Chen S, Chen T, Ni Y. Missing Value Imputation Approach for Mass Spectrometry-based Metabolomics Data. Sci Rep. 2018; 8:663. https://doi.org/10.1038/s41598-017-19120-0 PMID:29330539
