# Supplementary material for: Insights into serum metabolic biomarkers for early detection of incident diabetic kidney disease in Chinese patients with type 2 diabetes by random forest
Source: Aging (Albany NY). 2024 Feb 12;16(4):3420–530. doi: 10.18632/aging.205542 (PMC10929832; doi:10.18632/aging.205542)
Supplement: Supplementary Figures [file aging-16-205542-s002.pdf]

SUPPLEMENTARY FIGURES

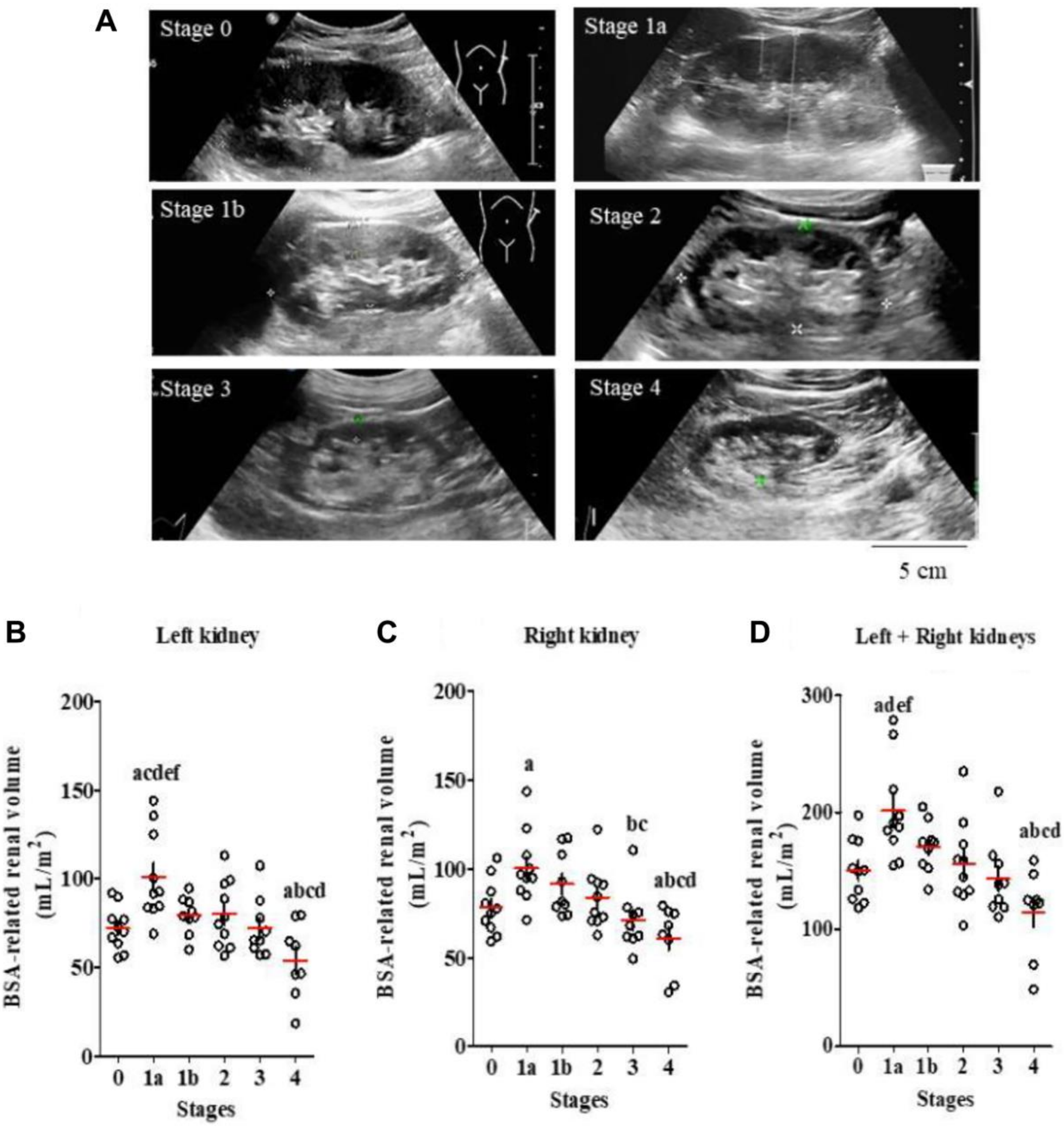

Supplementary Figure 1. (**A**) Images of ultrasonic scanning of the left kidneys in healthy subjects and diabetic patients in different stages and (**B**–**D**) the comparison of BSA-related renal volumes of both kidneys between stages in the validation cohort (significance level for superscript case letters: ^{a,b,c,d,e,f} represent comparison with Stages 0, 1a, 1b, 2, 3 and 4, respectively): Student's *t*-test, $p \leq 0.05$. Horizontal bars in the scatter plots represent mean ± SEM. $n = 10$ per stage.

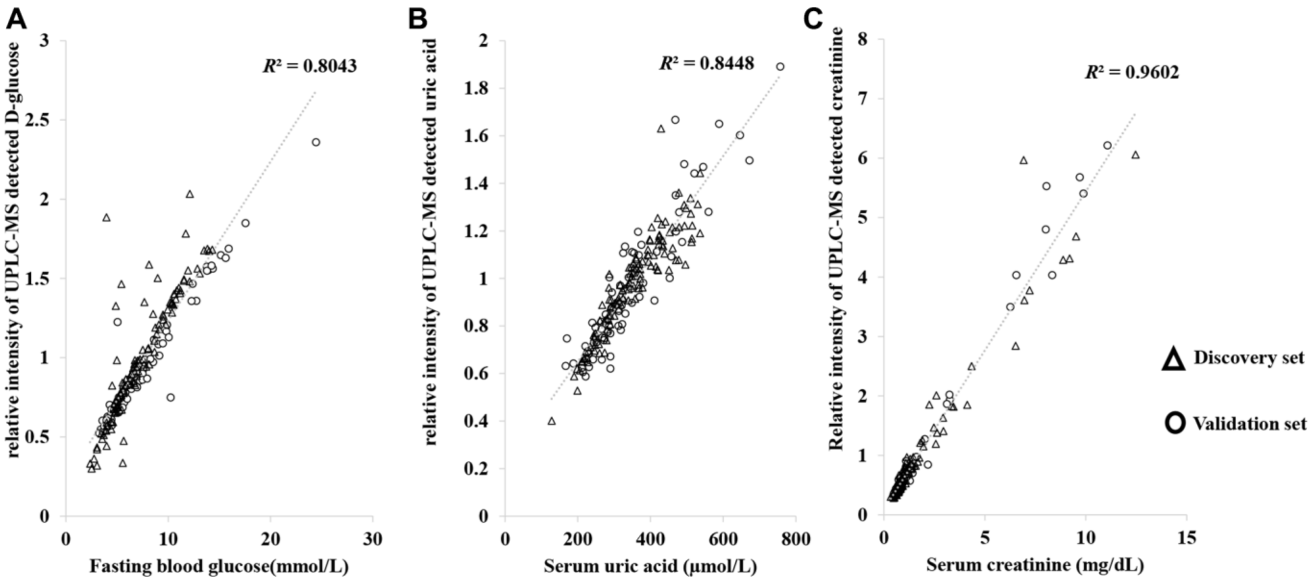

Supplementary Figure 2. Linear relationship of UPLC-Orbitrap-MS detected (**A**) serum D-glucose, (**B**) serum uric acid and (**C**) MS-detected serum creatinine against clinically measured FBG, serum uric acid, serum creatinine, respectively. Trendlines were formed with both cohorts.

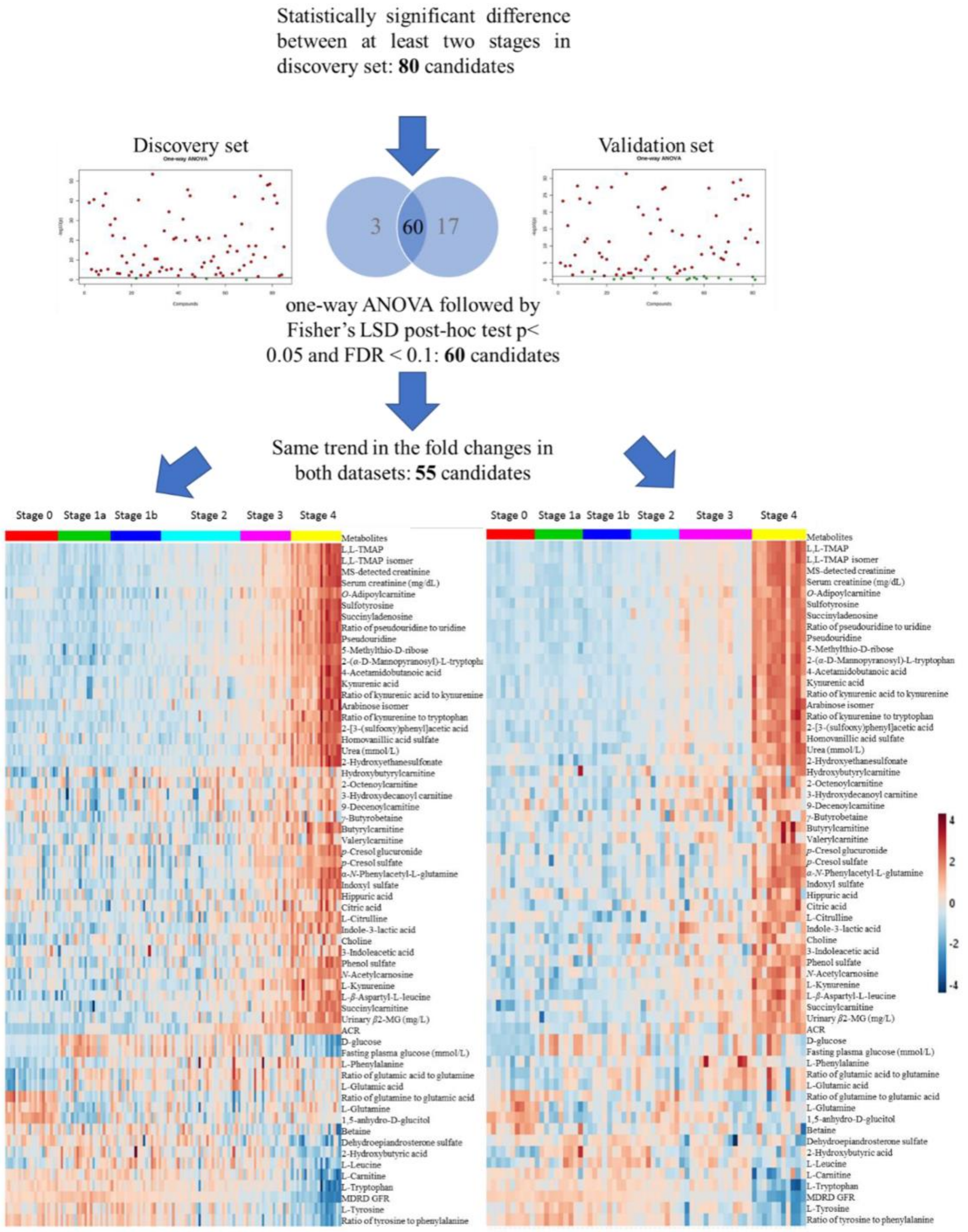

Supplementary Figure 3. Pipeline of screening of potential candidates.

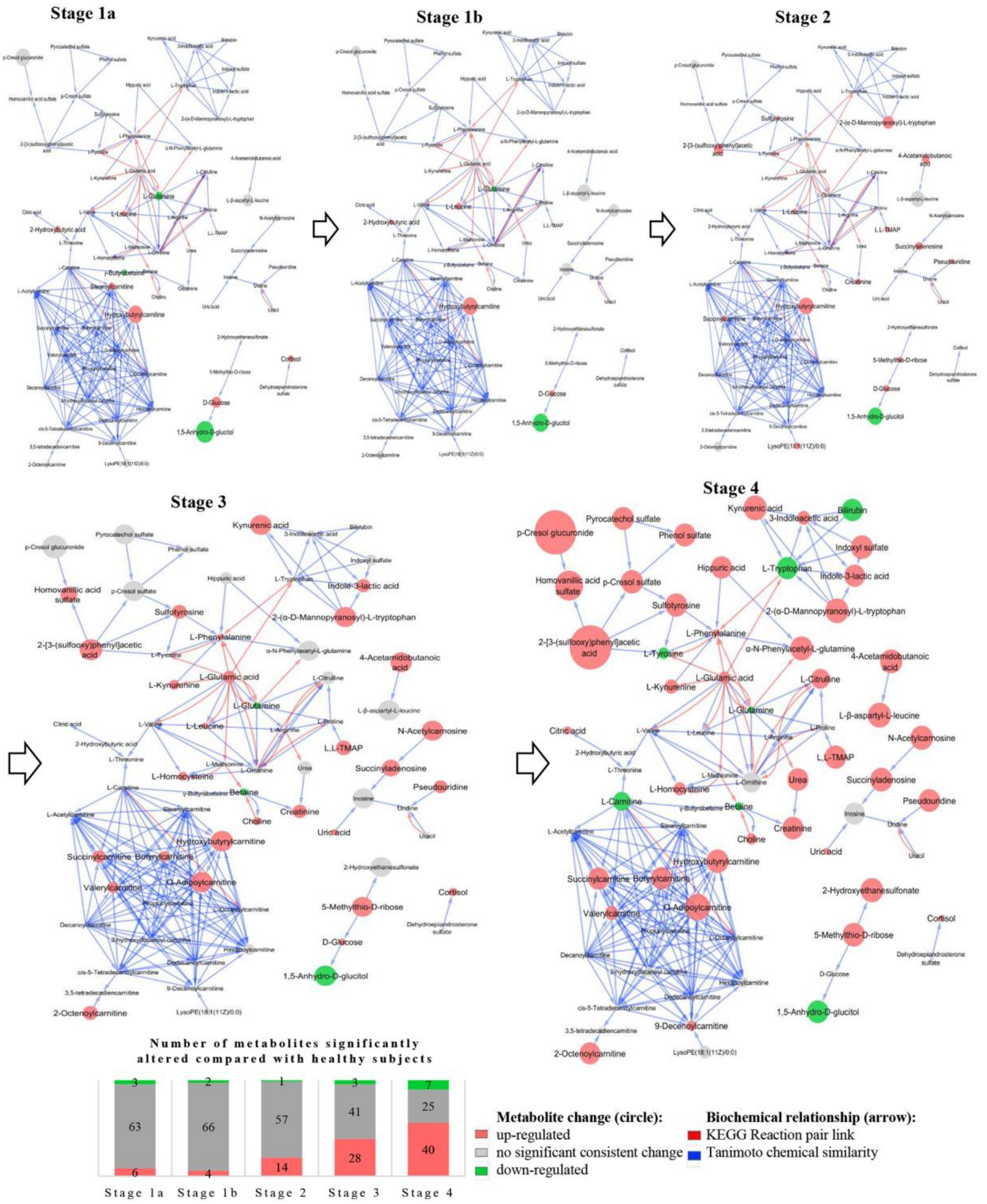

Supplementary Figure 4. Metabolomic network diagrams of SAMs. Metabolites of nucleoside, amino acid, glucose and short-medium-long chain carnitine were accumulated gradually with the progressed DKD. These metabolites were categorized into six classes: sulfate metabolites, amino acids and organic acids, acylcarnitine, purine derivatives, steroids, monosaccharides and their derivatives. Succinyladenosine and pseudouridine, originated from posttranslational modifications of nucleosides, increased significantly in DKD stage 4 patients. Uremic retention solutes like indoxyl sulfate and p-cresyl sulfate, which are products of dietary tryptophan and tyrosine respectively, are significantly elevated at DKD Stage 4. Tryptophan, tyrosine, phenylalanine, glutamine and citrulline are the significantly altered amino acid. Several metabolites decreased along with the declined eGFR, which were typtophan, tyrosine, glutamine, 1,5-anhydro-D-glucitol, carnitine, bilirubin and betaine. Red circle: upregulated metabolites; green circle: downregulated metabolites; grey circle denotes metabolites without significant or consistent changes in both cohorts. Circle sizes were proportional to the absolute value of fold change with respect to the normal group ($p \leq 0.05$).

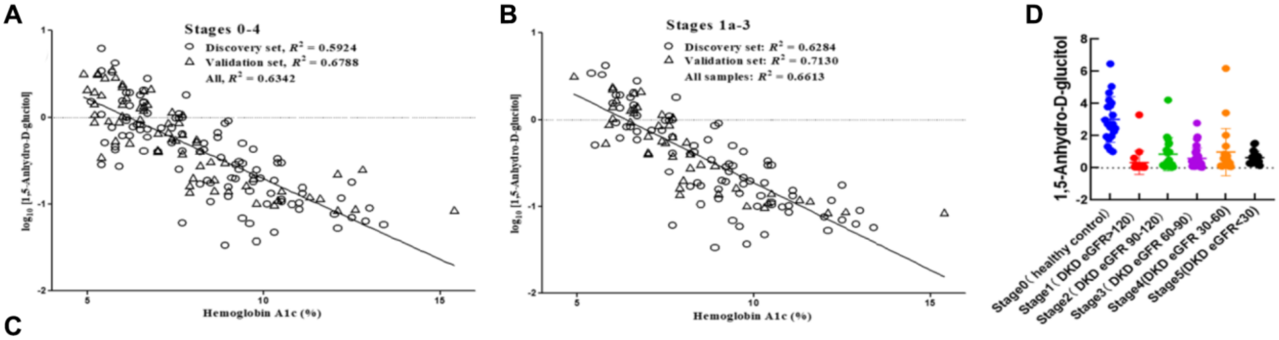

C

	Normal		Stage 1a		Stage 1b		Stage 2		Stage 3		Stage 4	
	Dis n = 20	Val n =10	Dis HbA1c: n = 18 FBG: n = 20	Val n = 10	Dis HbA1c: n = 17 FBG: n = 19	Val n = 10	Dis HbA1c: n = 29 FBG: n = 30	Val n = 10	Dis HbA1c: n = 17 FBG: n = 19	Val n = 15	Dis HbA1c: n = 19 FBG: n = 20	Val n = 11
	$\log_{10}$ (1,5-Anhydro-D-glucitol)											
**FBG**	0.184 ns	0.311 ns	-0.723	-0.865	-0.652	-0.766	-0.422	-0.650	-0.721	-0.585	0.225 ns	-0.603
**HbA1c**	-	0.534 ns	-0.821	-0.831	-0.874	-0.835	-0.639	-0.954	-0.839	-0.827	-0.445 ns	-0.669
**MDRD GFR**	0.141 ns	0.312 ns	-0.235 ns	-0.443 ns	0.420 ns	-0.448 ns	-0.043 ns	0.075 ns	-0.242 ns	0.052 ns	0.217 ns	0.587 ns
**UACR**	-0.342 ns		0.123ns		-0.091ns		-0.302ns		-0.108ns		0.023ns	

Supplementary Figure 5. Linear regression of UPLC-Orbitrap-MS detected log(1,5-anhydro-D-glucitol) against hemoglobin A1c in (**A**) Stages 0–4 and (**B**) Stages 1a-3 of DKD. (**C**) Correlation between 1,5-anhydro-D-glucitol, hemoglobin A1c, FBG, MS-detected D-glucose, MDRD GFR and UACR in each stage. (**D**) The scatter plots of 1,5-anhydro-D-glucitol among different stages. Abbreviations: Dis: discovery set; Val: validation set; ns: no statistical significance ($p > 0.05$). HbA1c: hemoglobin A1c. -, not available.

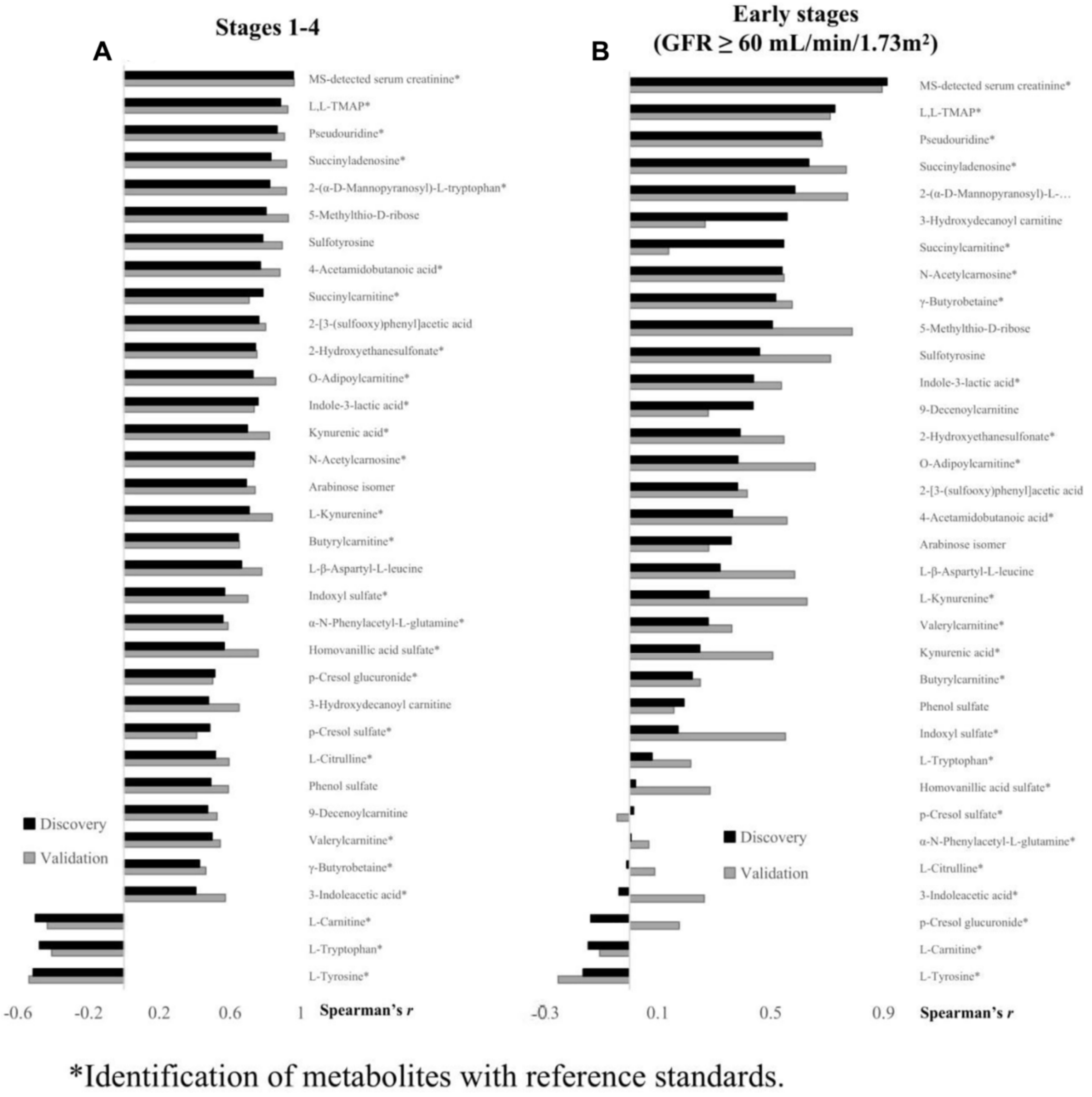

Supplementary Figure 6. List of metabolites that correlated (|r| ≥ 0.6) with eGFR at (**A**) Stages 1–4 and **B**) early stages (GFR ≥ 60 mL/min/1.73 m^2) patients. Metabolites showed strong correlation with eGFR, especially in early diabetic patients.

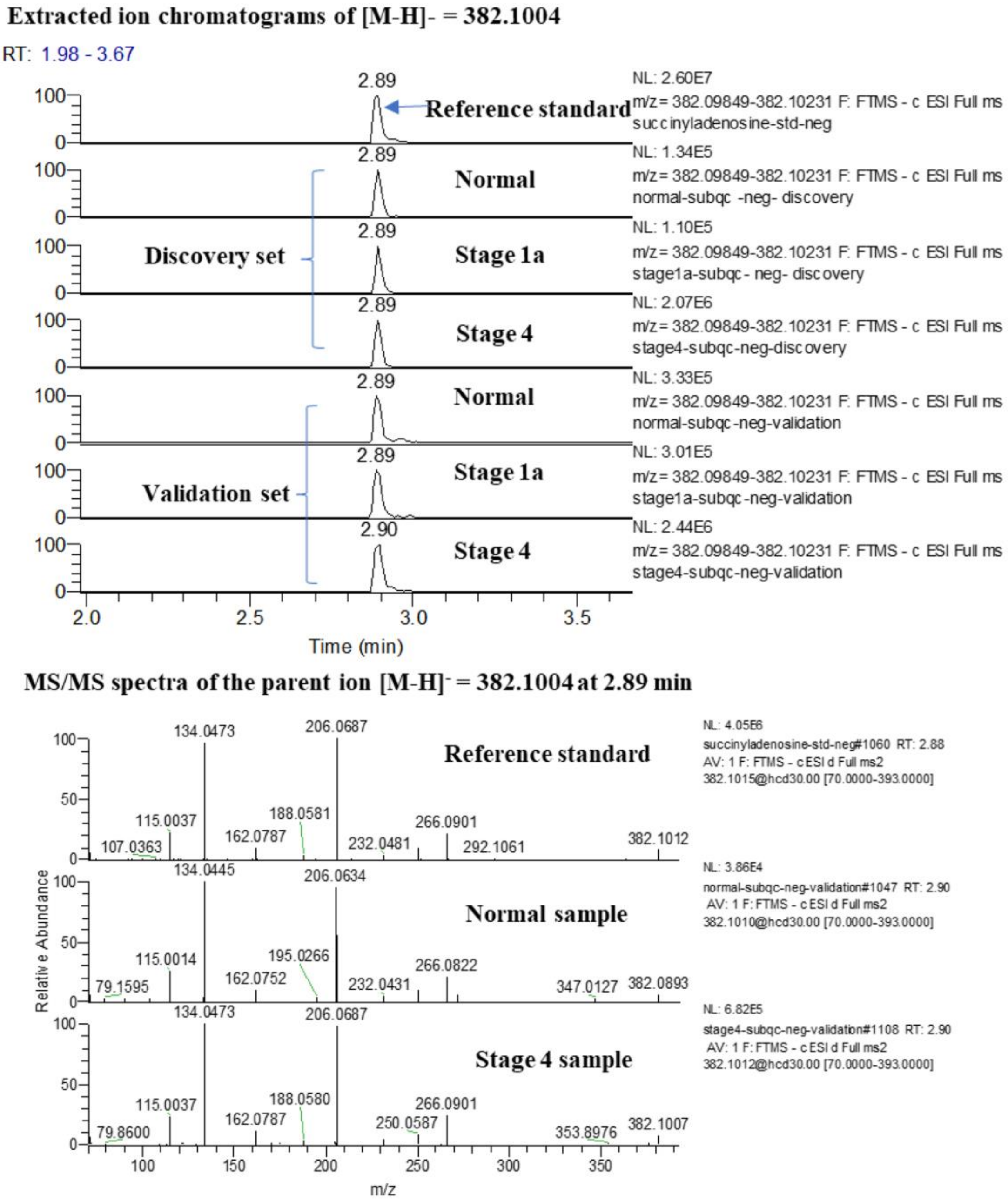

Supplementary Figure 7. Demonstration of peak identification of the adduct (M-H)- of succinyladenosine compared with its reference standard.

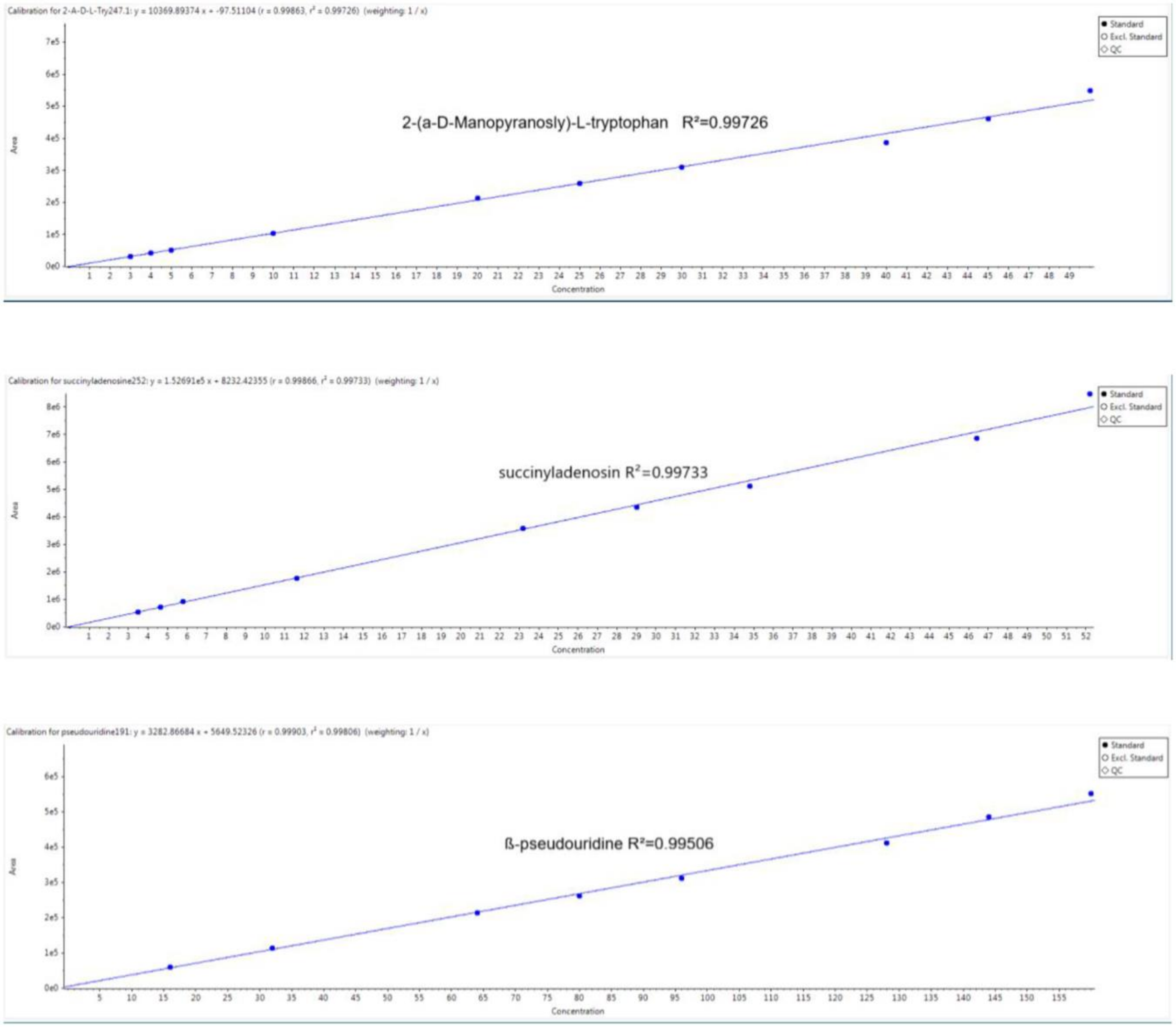

Supplementary Figure 8. Calibration curves of selected metabolites.

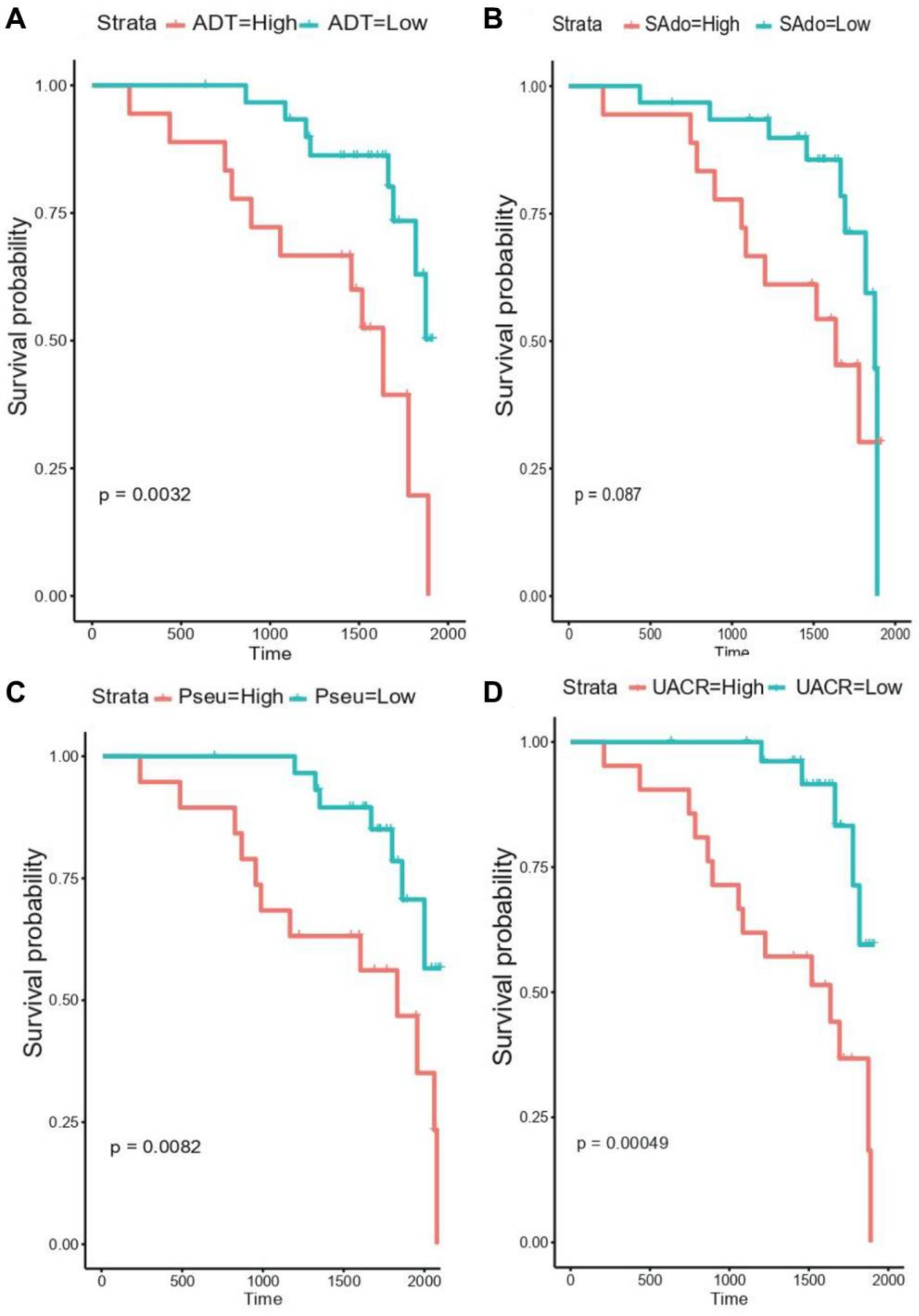

Supplementary Figure 9. Survival curve of follow-up cohort using the baseline levels of three CDBs and UACR. (**A**) ADT; (**B**) SAdo; (**C**) pseudouridine; (**D**) UACR.

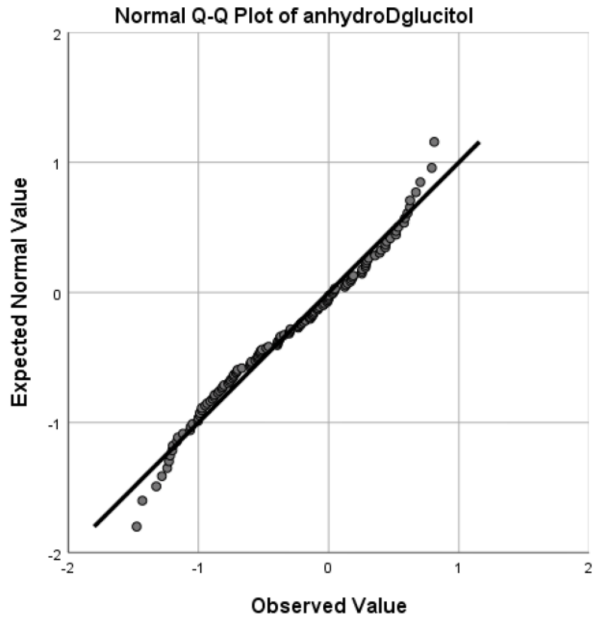

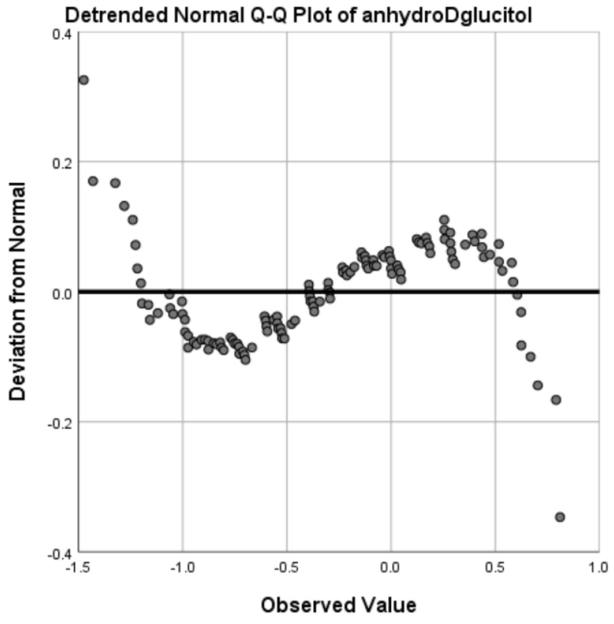

Supplementary Figure 10. The quantile-quantile (Q-Q) plot of normalized anhydroDglucitol which demonstrated the consistency of normalized value with expected normal value (upper panel) and the deviation of normalized values from expected normal value (lower panel).

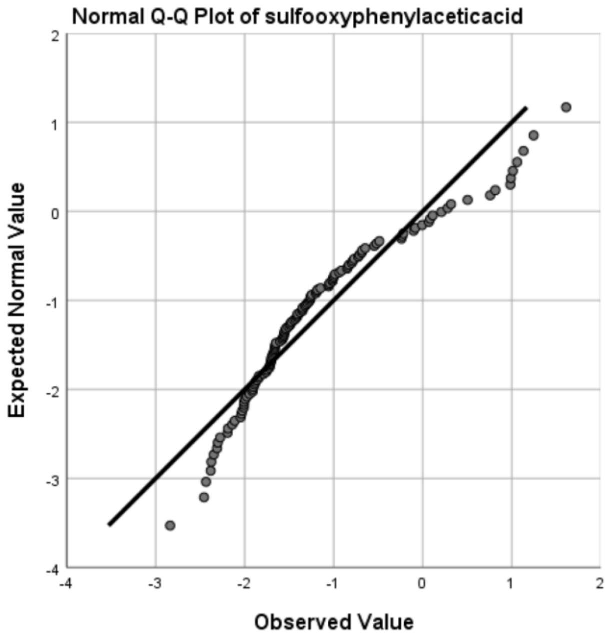

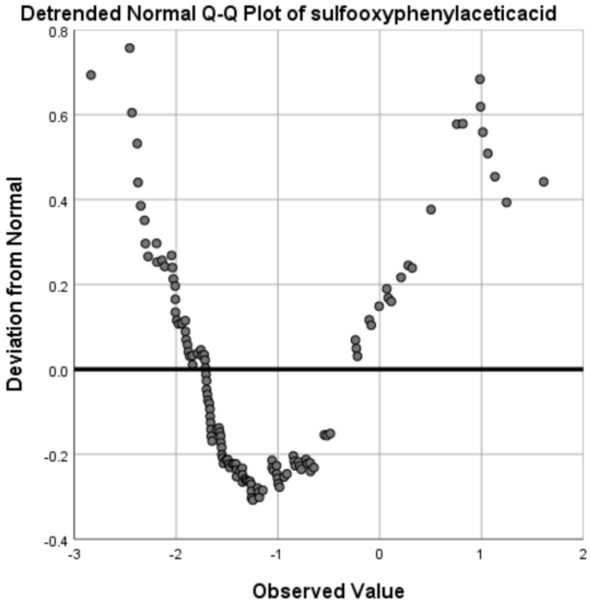

Supplementary Figure 11. The quantile-quantile (Q-Q) plot of normalized sulfooxyphenylaceticacid which demonstrated the consistency of normalized value with expected normal value (upper panel) and the deviation of normalized values from expected normal value (lower panel).

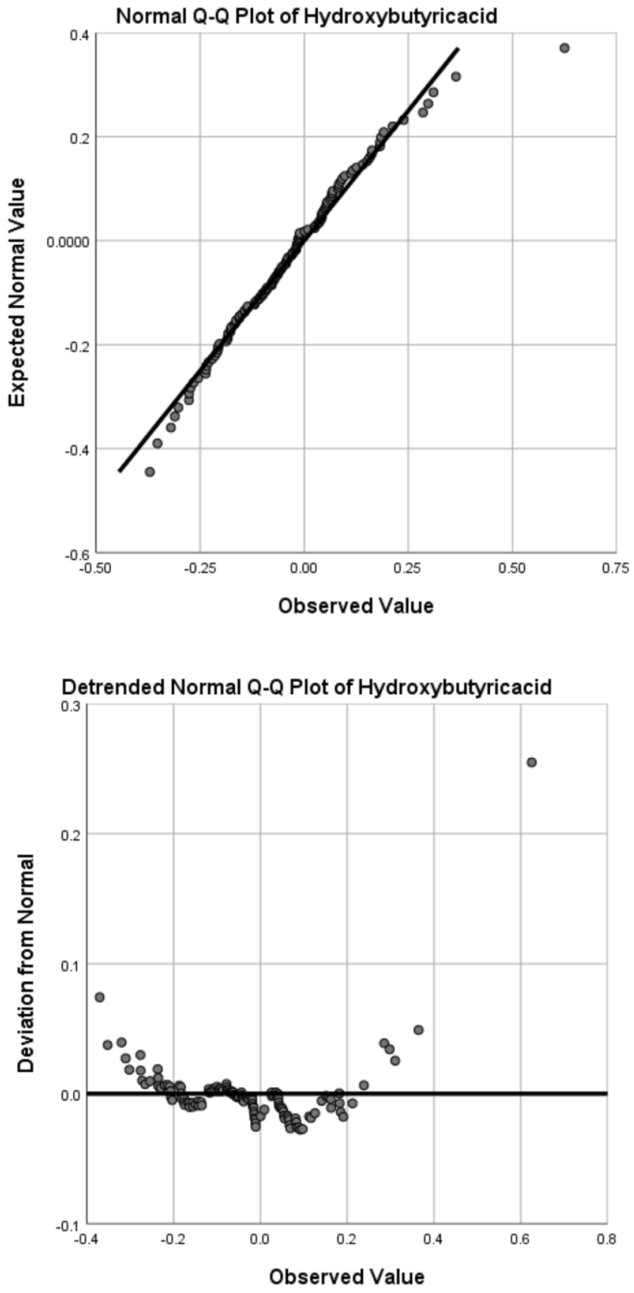

Supplementary Figure 12. The quantile-quantile (Q-Q) plot of normalized Hydroxybutyricacid which demonstrated the consistency of normalized value with expected normal value (upper panel) and the deviation of normalized values from expected normal value (lower panel).

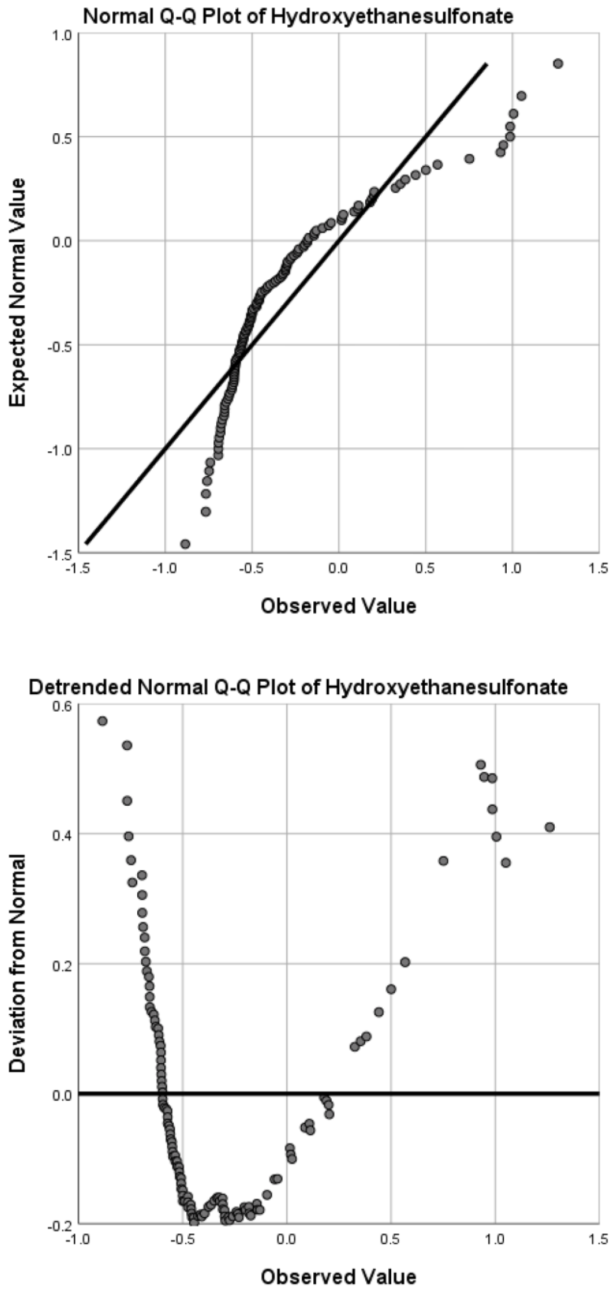

Supplementary Figure 13. The quantile-quantile (Q-Q) plot of normalized Hydroxyethanesulfonate which demonstrated the consistency of normalized value with expected normal value (upper panel) and the deviation of normalized values from expected normal value (lower panel).

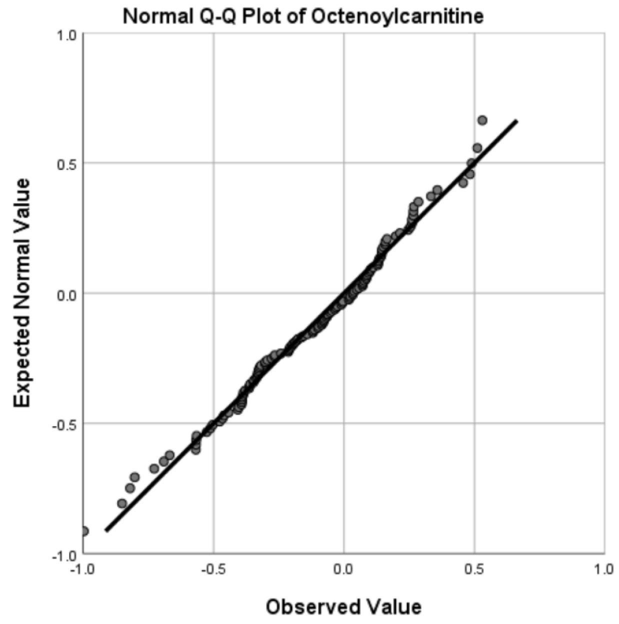

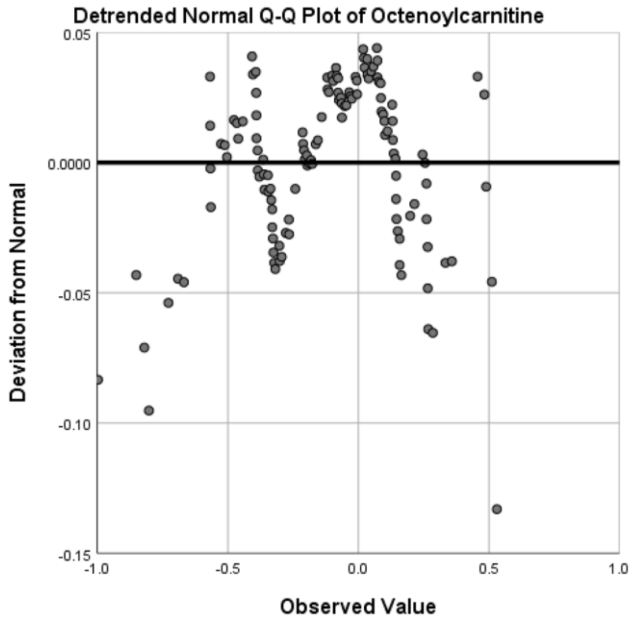

Supplementary Figure 14. The quantile-quantile (Q-Q) plot of normalized Octenoylcarnitine which demonstrated the consistency of normalized value with expected normal value (upper panel) and the deviation of normalized values from expected normal value (lower panel).

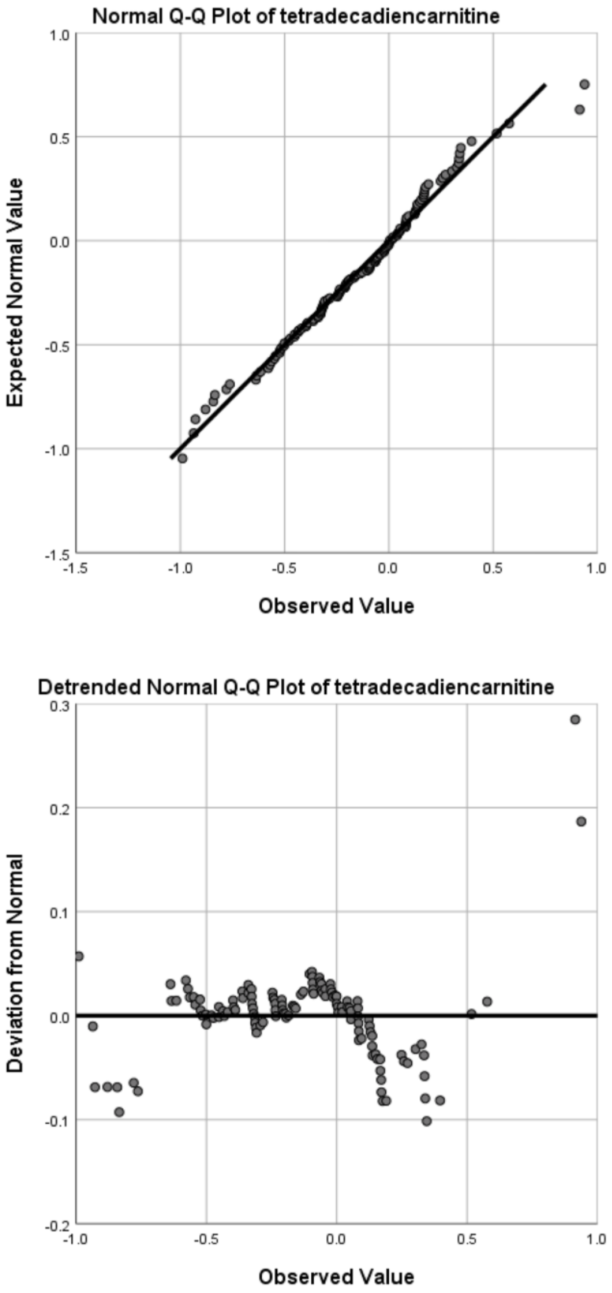

Supplementary Figure 15. The quantile-quantile (Q-Q) plot of normalized tetradecadiencarnitine which demonstrated the consistency of normalized value with expected normal value (upper panel) and the deviation of normalized values from expected normal value (lower panel).

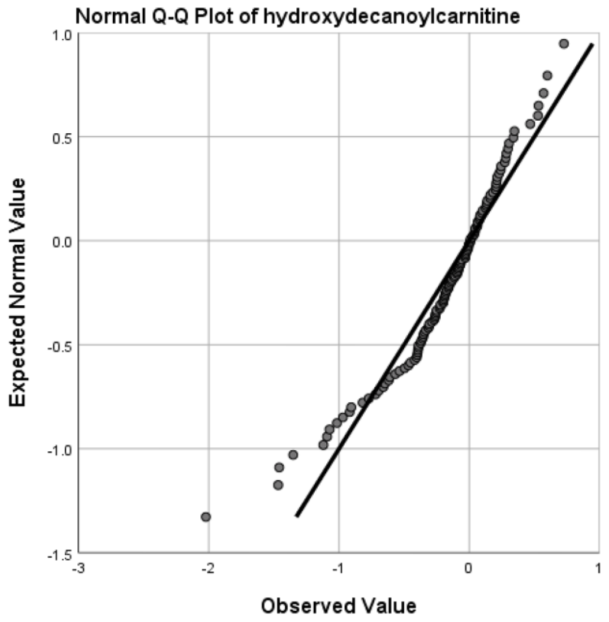

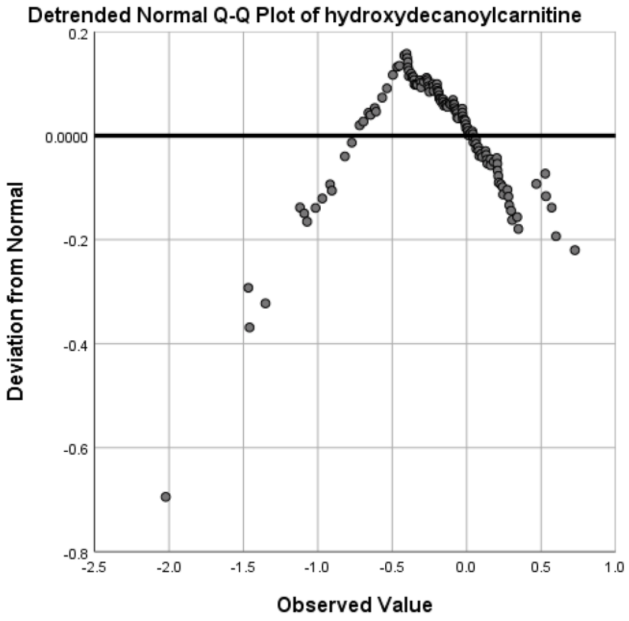

Supplementary Figure 16. The quantile-quantile (Q-Q) plot of normalized hydroxydecanoylcarnitine which demonstrated the consistency of normalized value with expected normal value (upper panel) and the deviation of normalized values from expected normal value (lower panel).

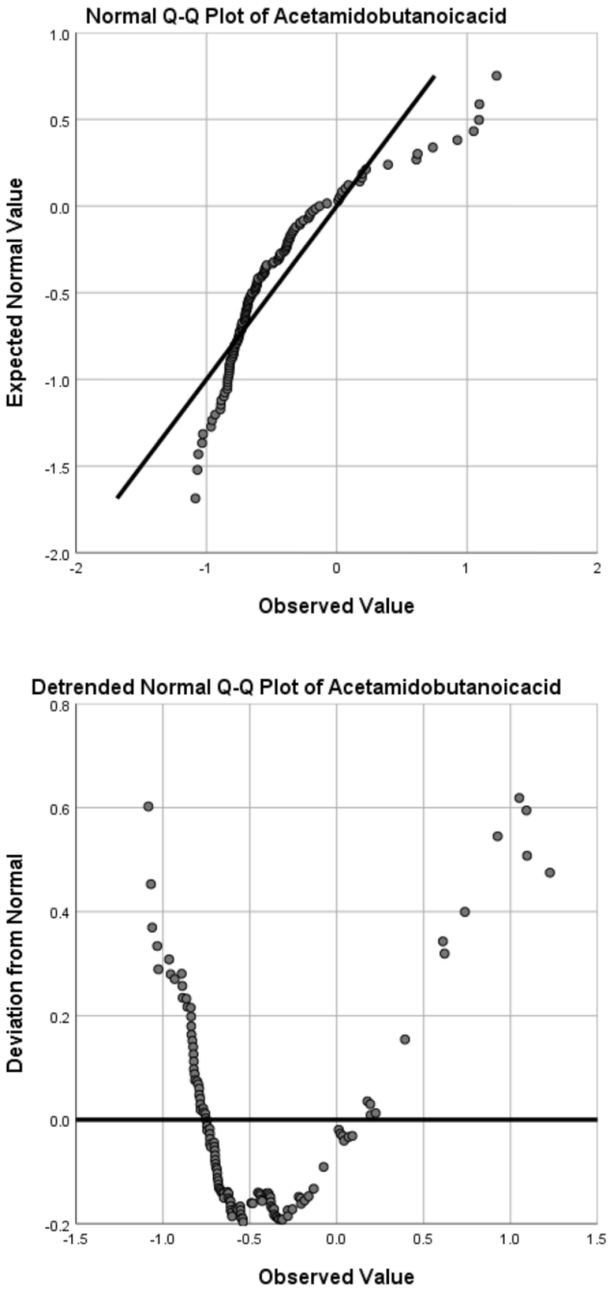

Supplementary Figure 17. The quantile-quantile (Q-Q) plot of normalized Acetamidobutanoicacid which demonstrated the consistency of normalized value with expected normal value (upper panel) and the deviation of normalized values from expected normal value (lower panel).

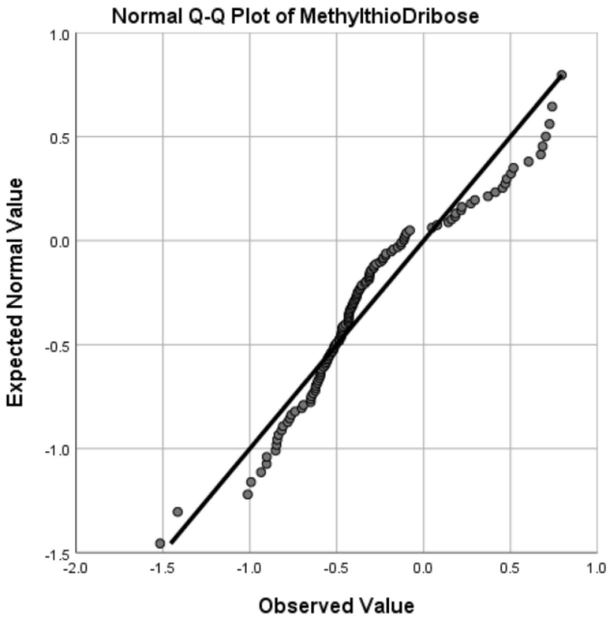

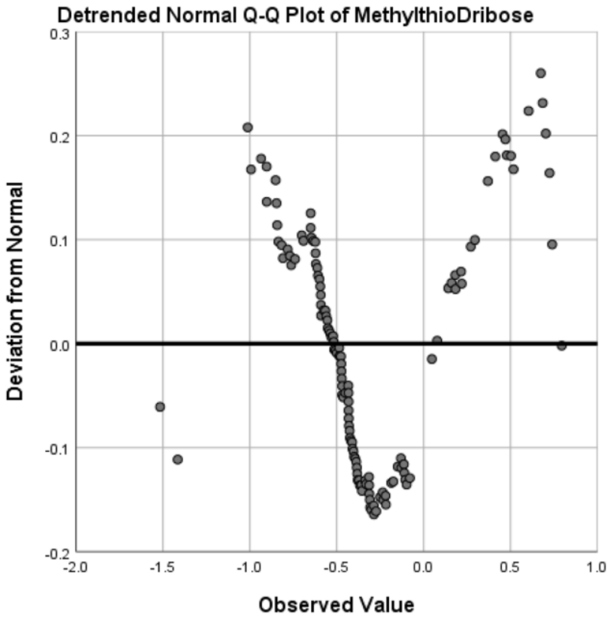

Supplementary Figure 18. The quantile-quantile (Q-Q) plot of normalized MethylthioDribose which demonstrated the consistency of normalized value with expected normal value (upper panel) and the deviation of normalized values from expected normal value (lower panel).

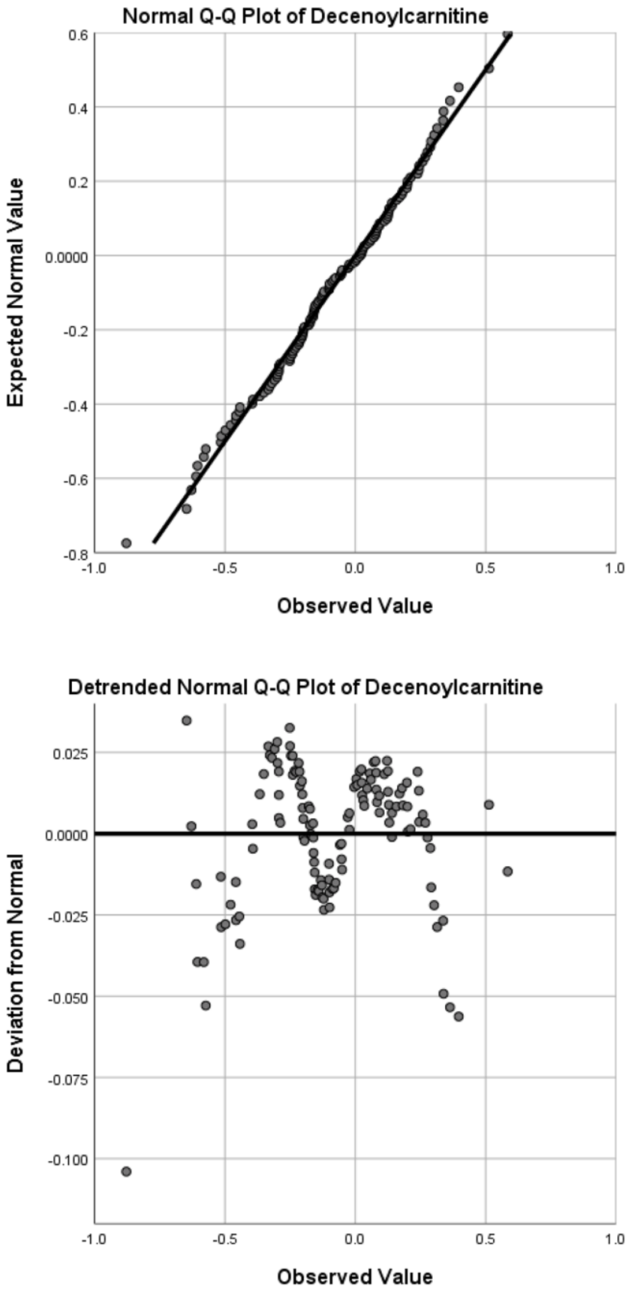

Supplementary Figure 19. The quantile-quantile (Q-Q) plot of normalized Decenoylcarnitine which demonstrated the consistency of normalized value with expected normal value (upper panel) and the deviation of normalized values from expected normal value (lower panel).

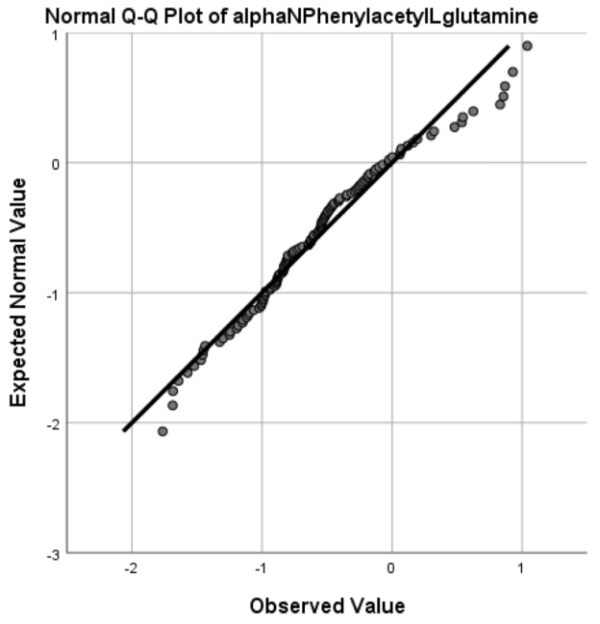

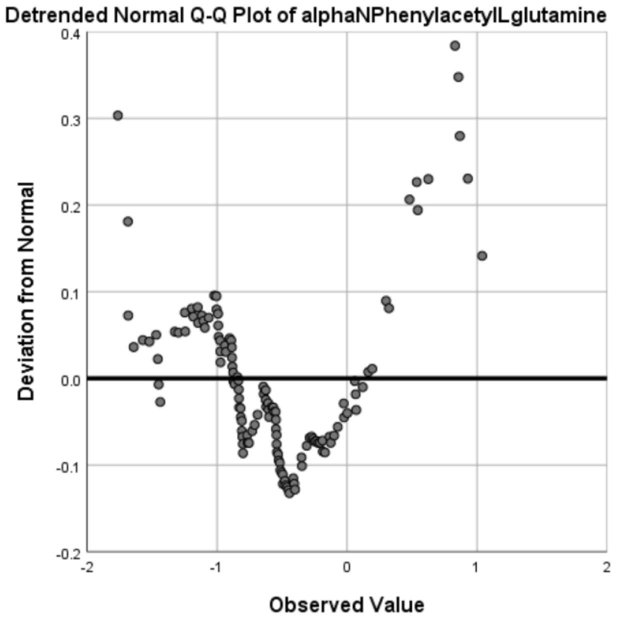

Supplementary Figure 20. The quantile-quantile (Q-Q) plot of normalized alphaNPhenylacetylLglutamine which demonstrated the consistency of normalized value with expected normal value (upper panel) and the deviation of normalized values from expected normal value (lower panel).

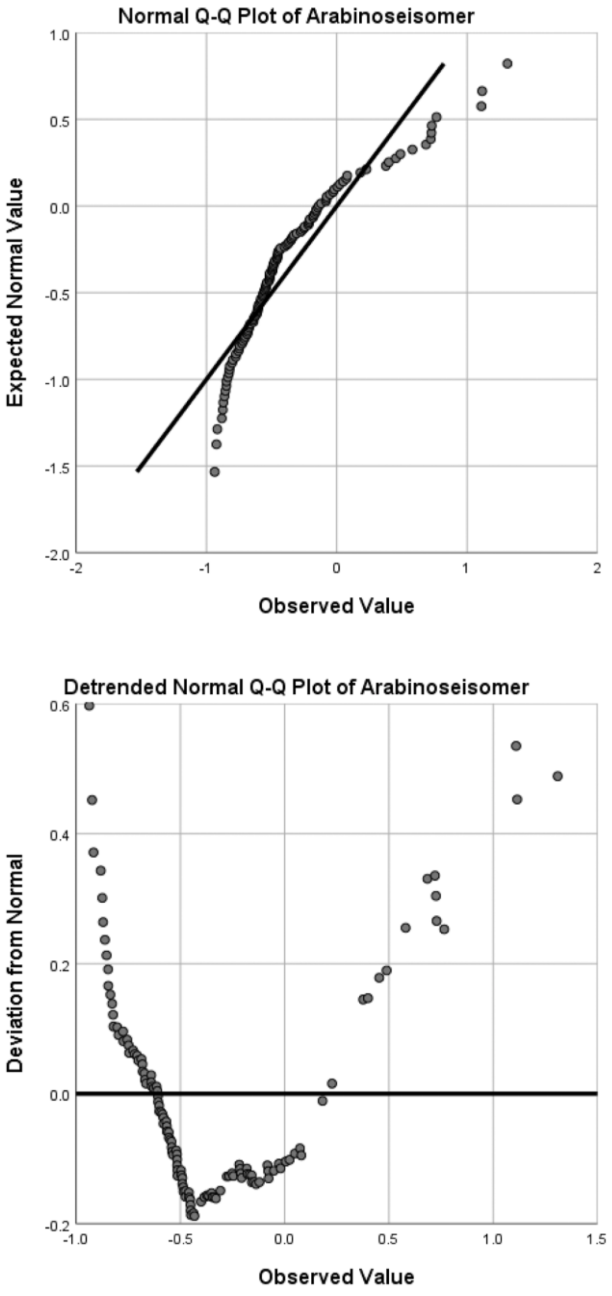

Supplementary Figure 21. The quantile-quantile (Q-Q) plot of normalized Arabinoseisomer which demonstrated the consistency of normalized value with expected normal value (upper panel) and the deviation of normalized values from expected normal value (lower panel).

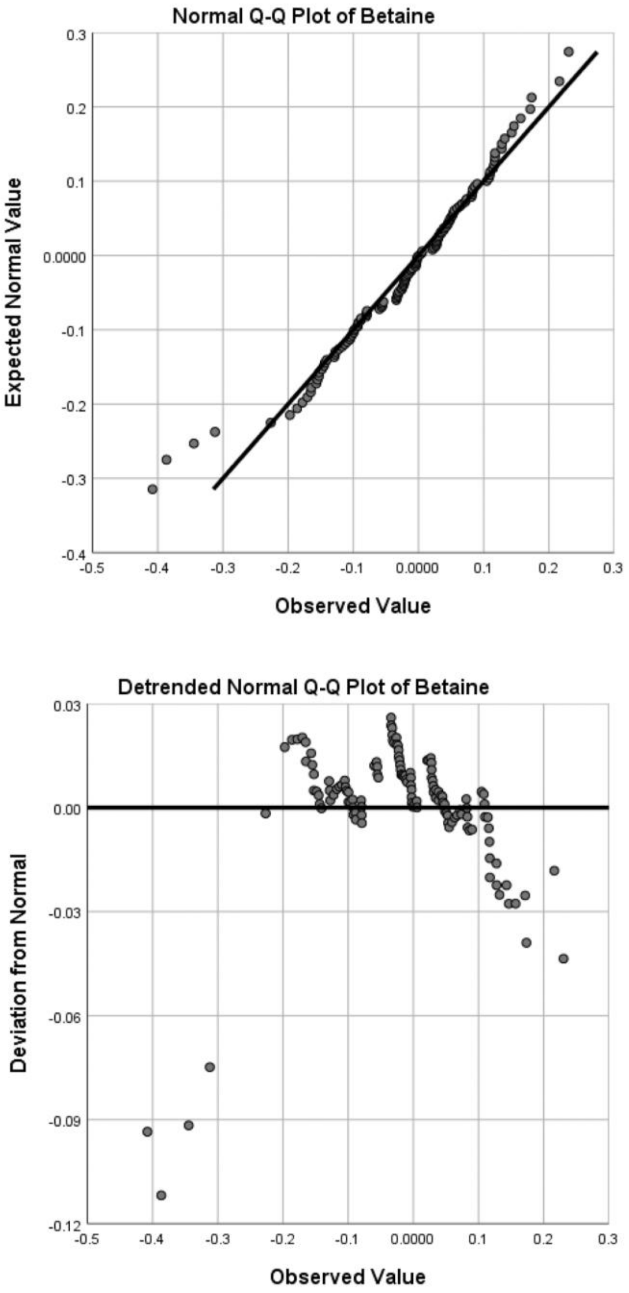

Supplementary Figure 22. The quantile-quantile (Q-Q) plot of normalized Betaine which demonstrated the consistency of normalized value with expected normal value (upper panel) and the deviation of normalized values from expected normal value (lower panel).

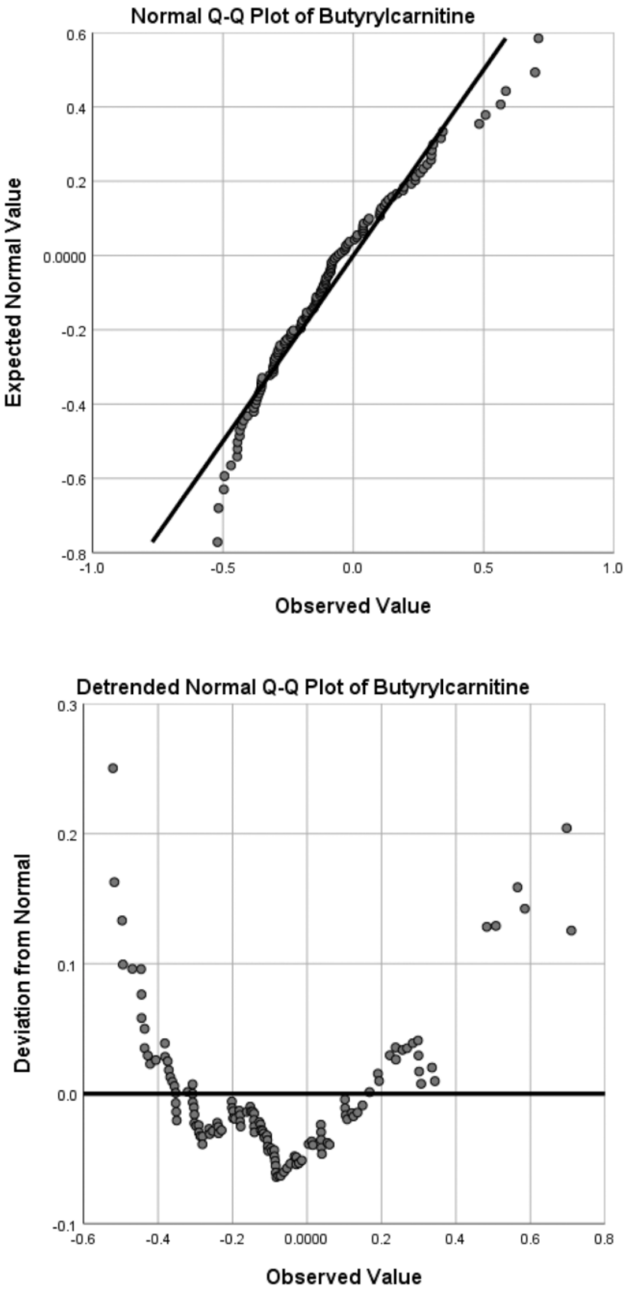

Supplementary Figure 23. The quantile-quantile (Q-Q) plot of normalized Butyrylcarnitine which demonstrated the consistency of normalized value with expected normal value (upper panel) and the deviation of normalized values from expected normal value (lower panel).

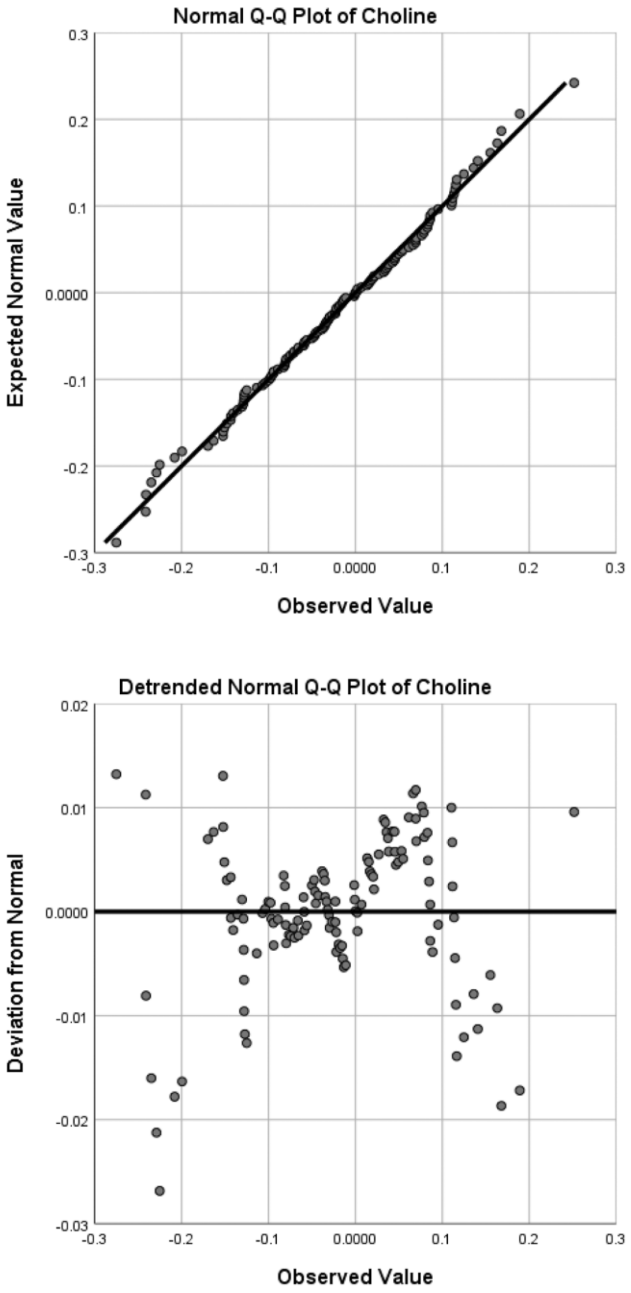

Supplementary Figure 24. The quantile-quantile (Q-Q) plot of normalized Choline which demonstrated the consistency of normalized value with expected normal value (upper panel) and the deviation of normalized values from expected normal value (lower panel).

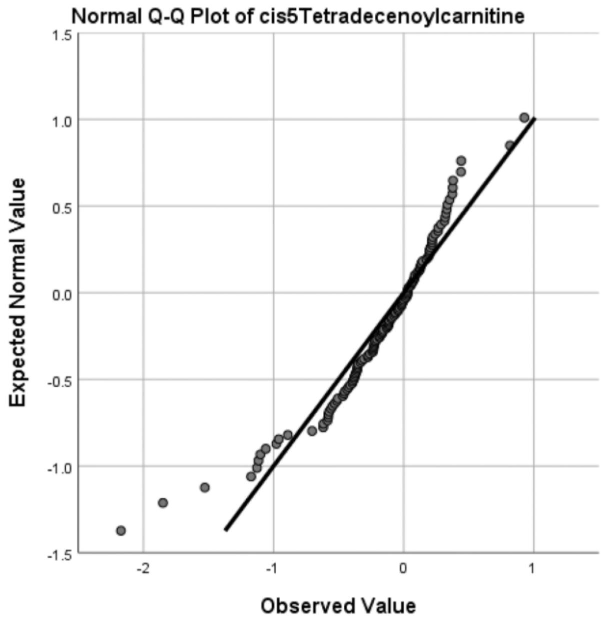

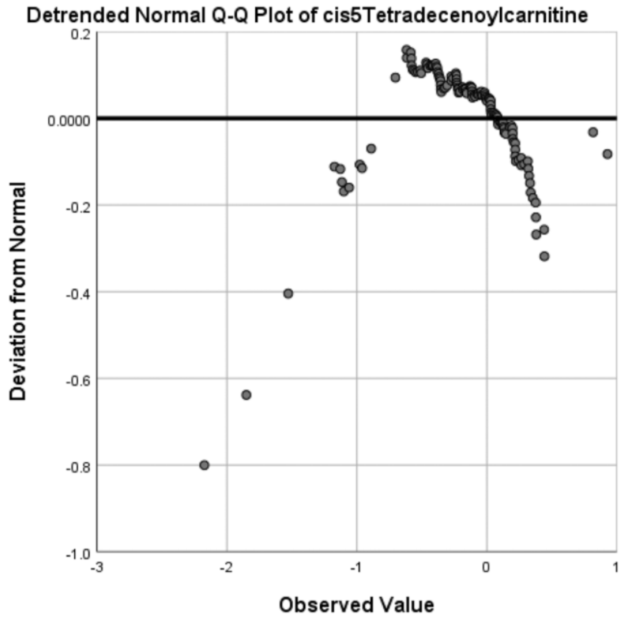

Supplementary Figure 25. The quantile-quantile (Q-Q) plot of normalized cis5Tetradecenoylcarnitine which demonstrated the consistency of normalized value with expected normal value (upper panel) and the deviation of normalized values from expected normal value (lower panel).

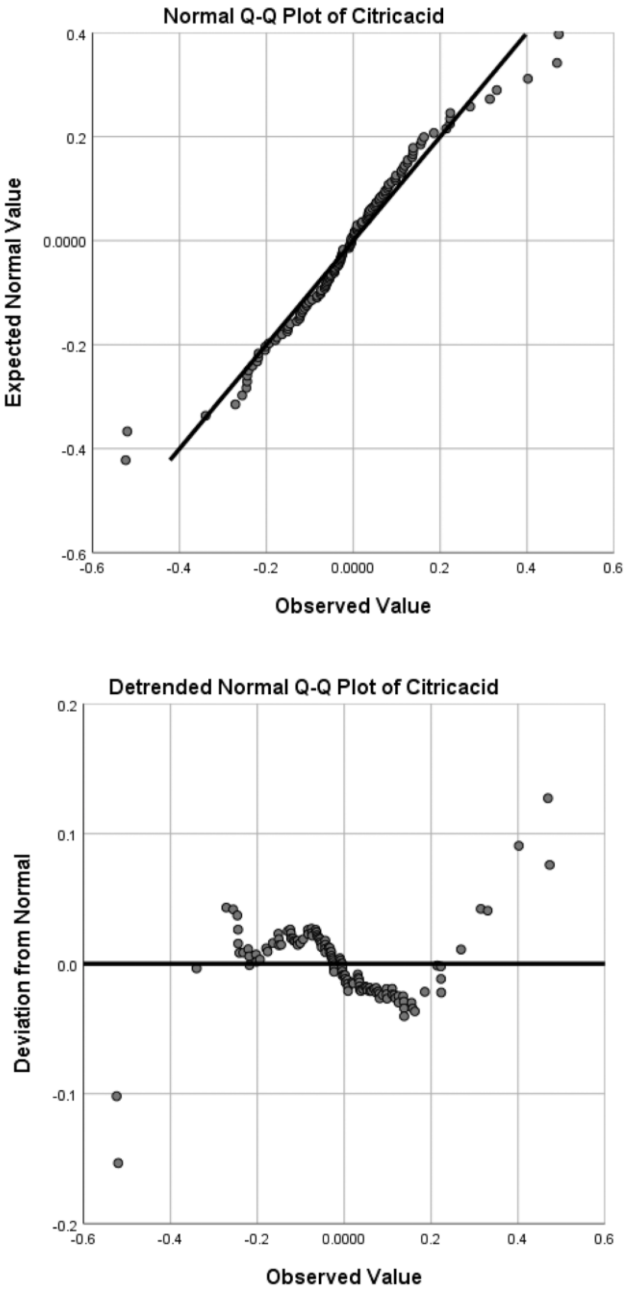

Supplementary Figure 26. The quantile-quantile (Q-Q) plot of normalized Citricacid which demonstrated the consistency of normalized value with expected normal value (upper panel) and the deviation of normalized values from expected normal value (lower panel).

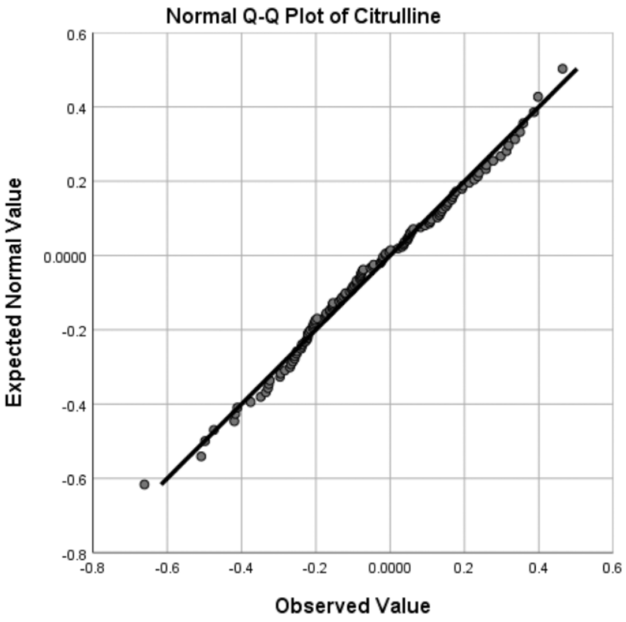

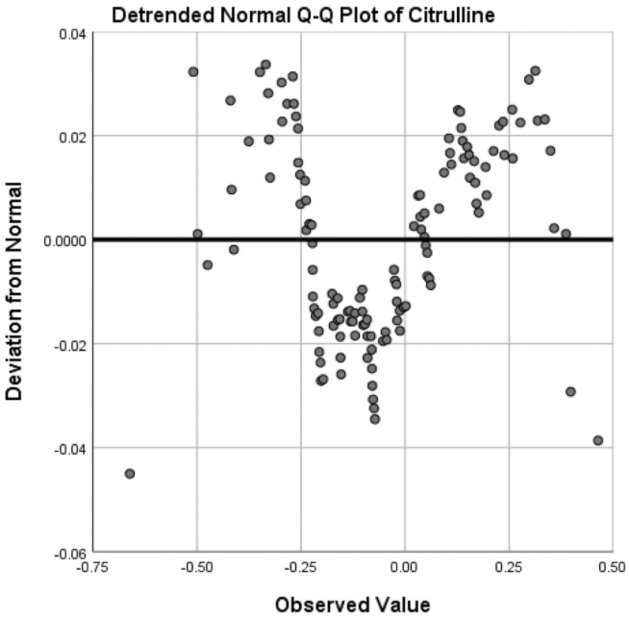

Supplementary Figure 27. The quantile-quantile (Q-Q) plot of normalized Citrulline which demonstrated the consistency of normalized value with expected normal value (upper panel) and the deviation of normalized values from expected normal value (lower panel).

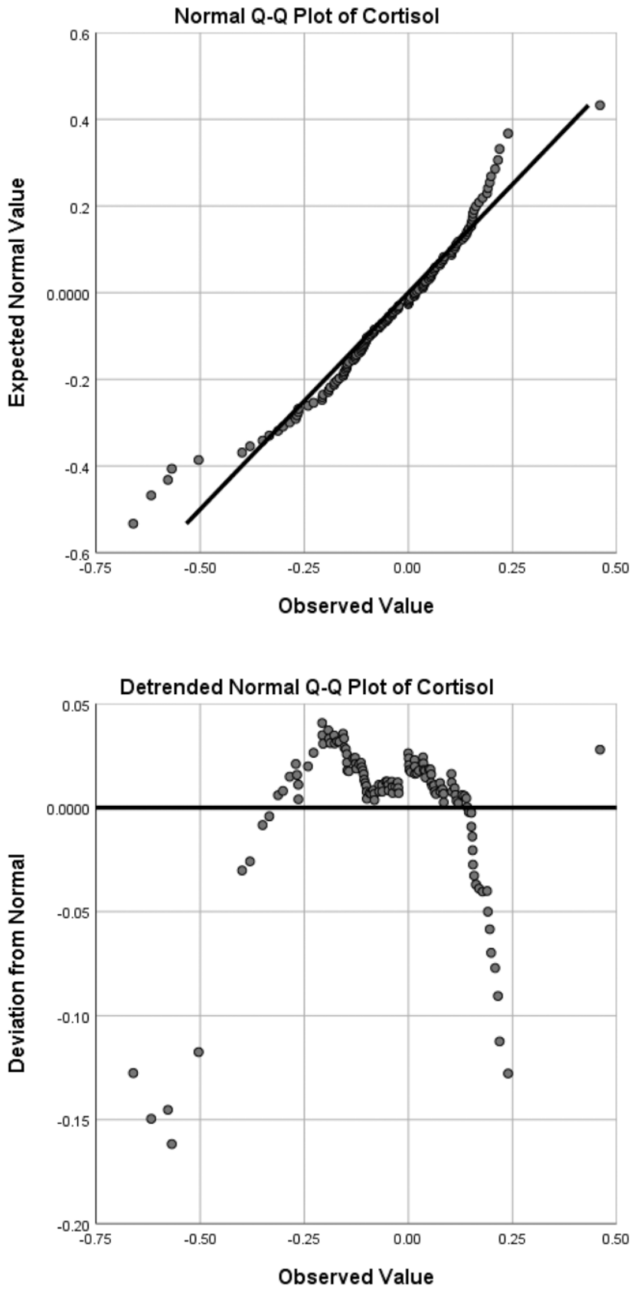

Supplementary Figure 28. The quantile-quantile (Q-Q) plot of normalized Cortisol which demonstrated the consistency of normalized value with expected normal value (upper panel) and the deviation of normalized values from expected normal value (lower panel).

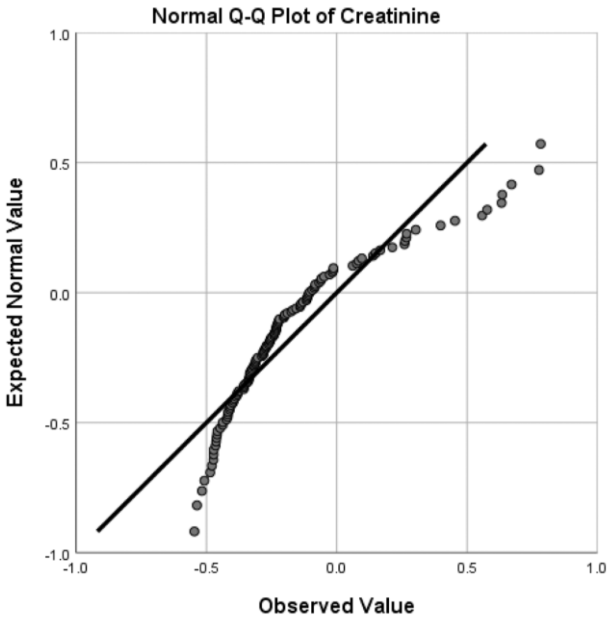

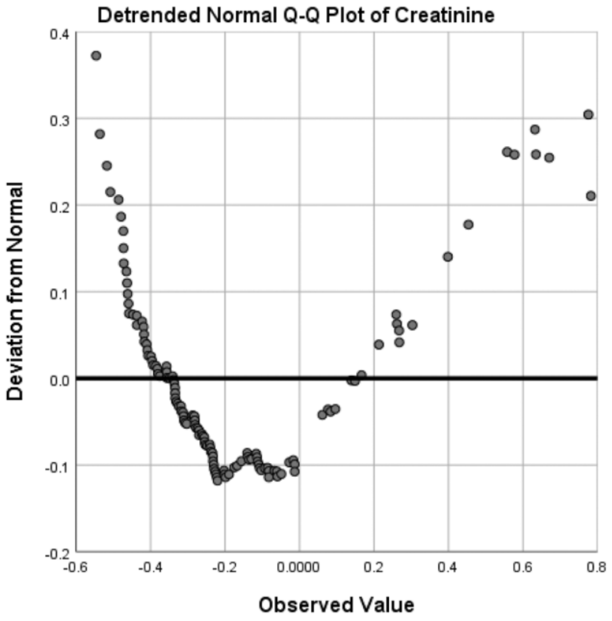

Supplementary Figure 29. The quantile-quantile (Q-Q) plot of normalized Creatinine which demonstrated the consistency of normalized value with expected normal value (upper panel) and the deviation of normalized values from expected normal value (lower panel).

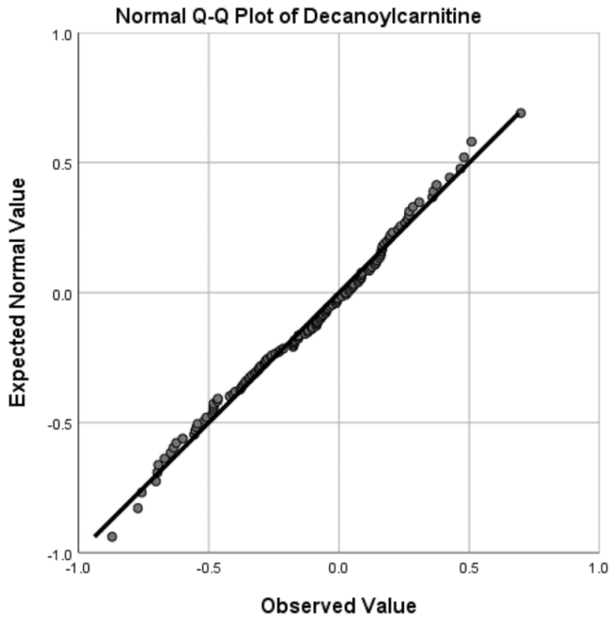

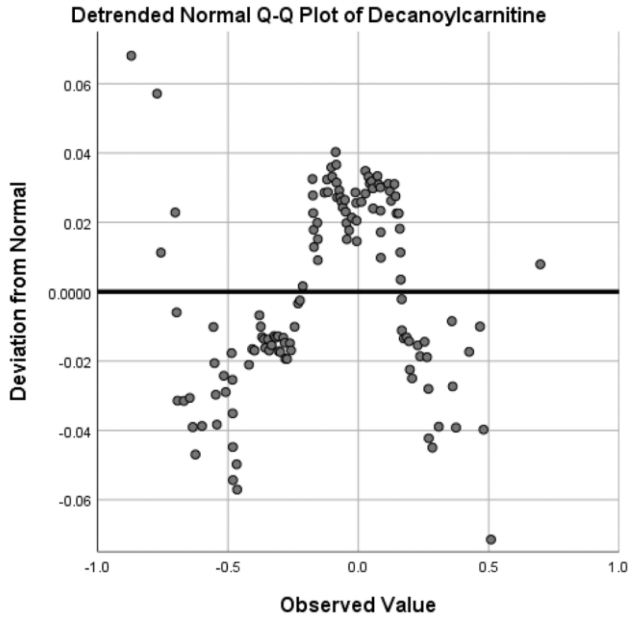

Supplementary Figure 30. The quantile-quantile (Q-Q) plot of normalized Decanoylcarnitine which demonstrated the consistency of normalized value with expected normal value (upper panel) and the deviation of normalized values from expected normal value (lower panel).

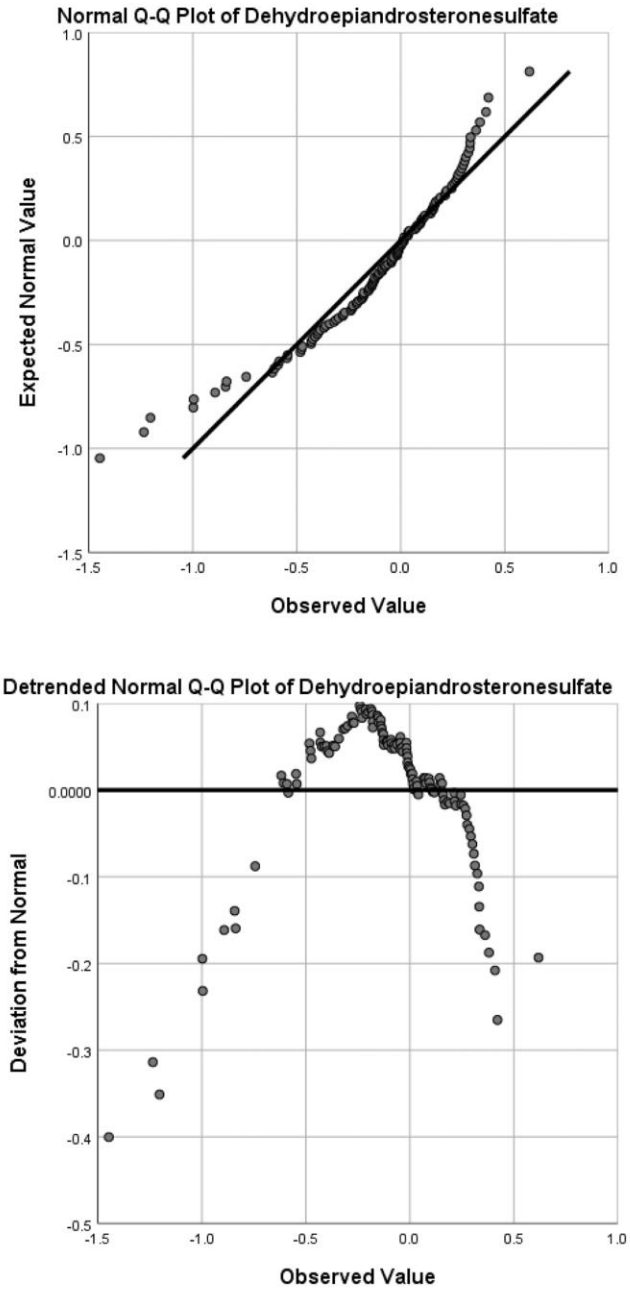

Supplementary Figure 31. The quantile-quantile (Q-Q) plot of normalized Dehydroepiandrosteronesulfate which demonstrated the consistency of normalized value with expected normal value (upper panel) and the deviation of normalized values from expected normal value (lower panel).

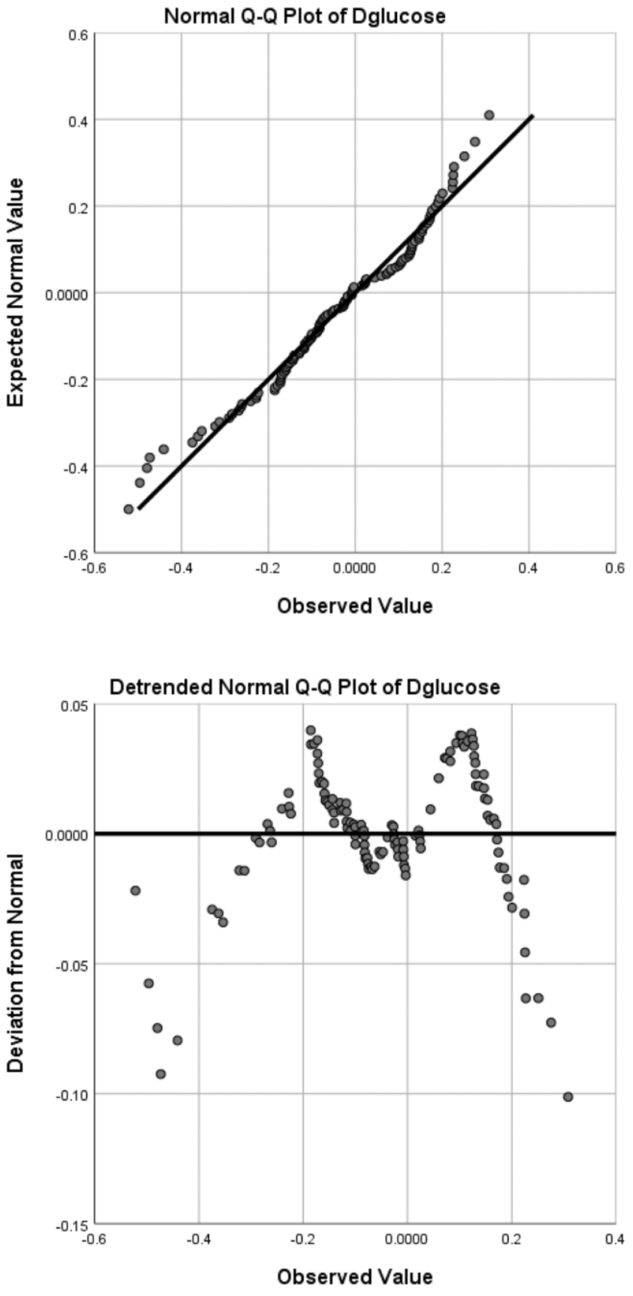

Supplementary Figure 32. The quantile-quantile (Q-Q) plot of normalized Dglucose which demonstrated the consistency of normalized value with expected normal value (upper panel) and the deviation of normalized values from expected normal value (lower panel).

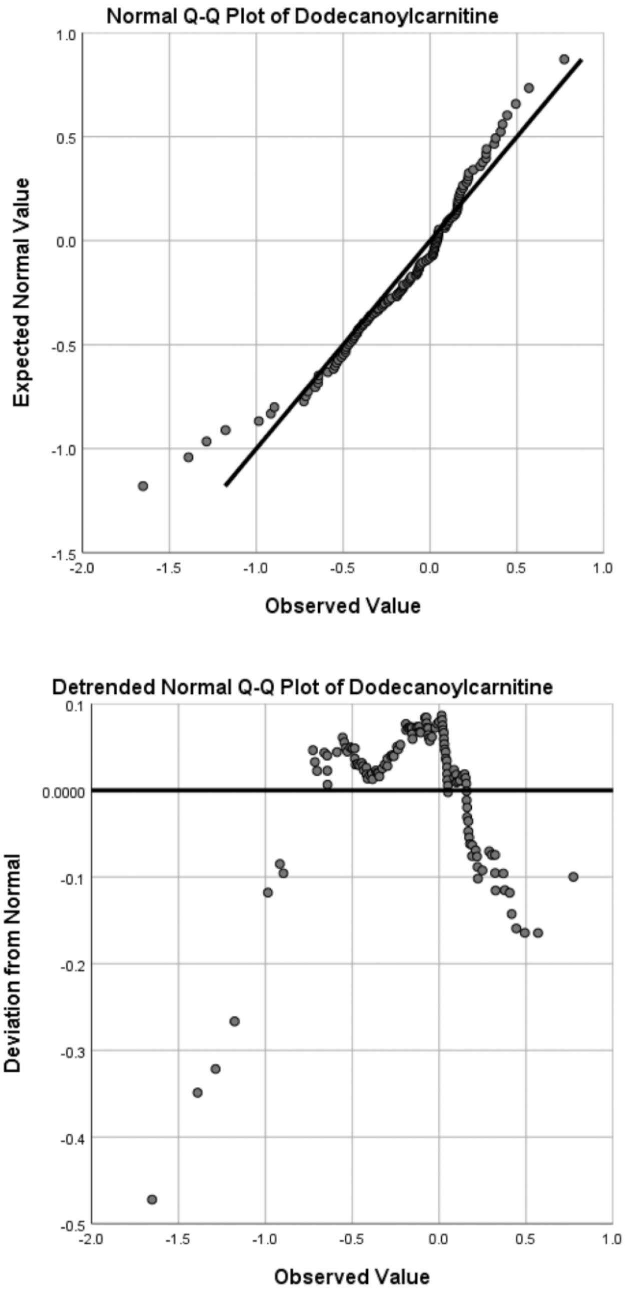

Supplementary Figure 33. The quantile-quantile (Q-Q) plot of normalized Dodecanoylcarnitine which demonstrated the consistency of normalized value with expected normal value (upper panel) and the deviation of normalized values from expected normal value (lower panel).

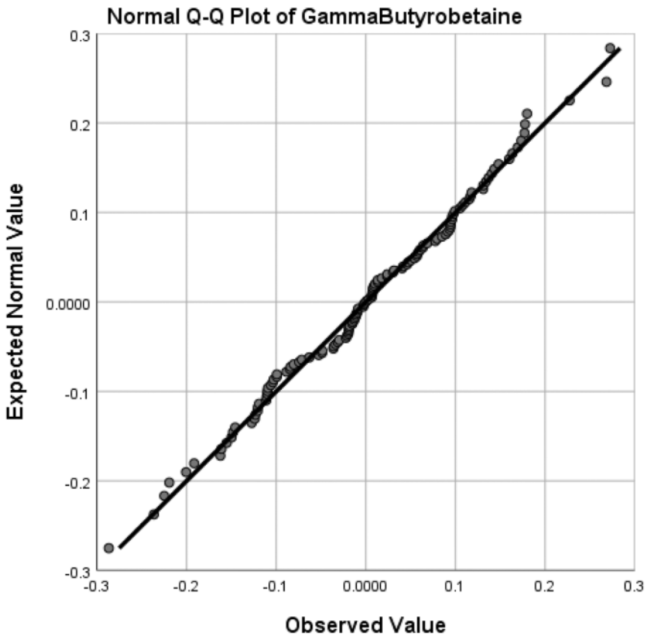

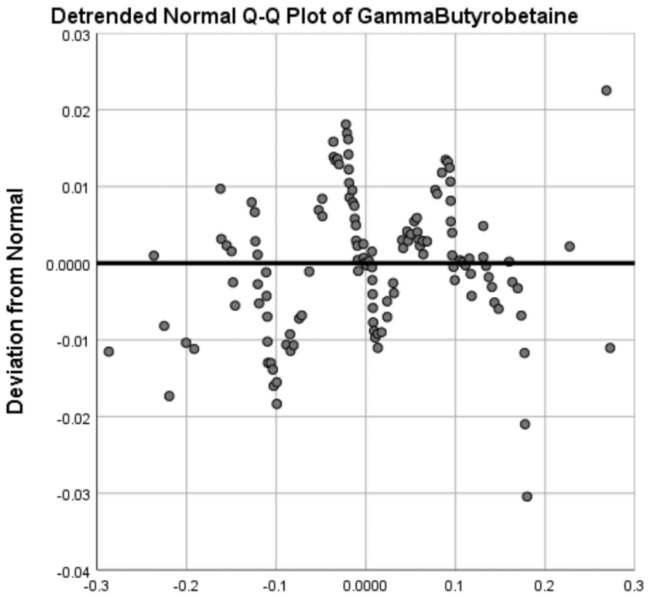

Supplementary Figure 34. The quantile-quantile (Q-Q) plot of normalized GammerButyrobetaine which demonstrated the consistency of normalized value with expected normal value (upper panel) and the deviation of normalized values from expected normal value (lower panel).

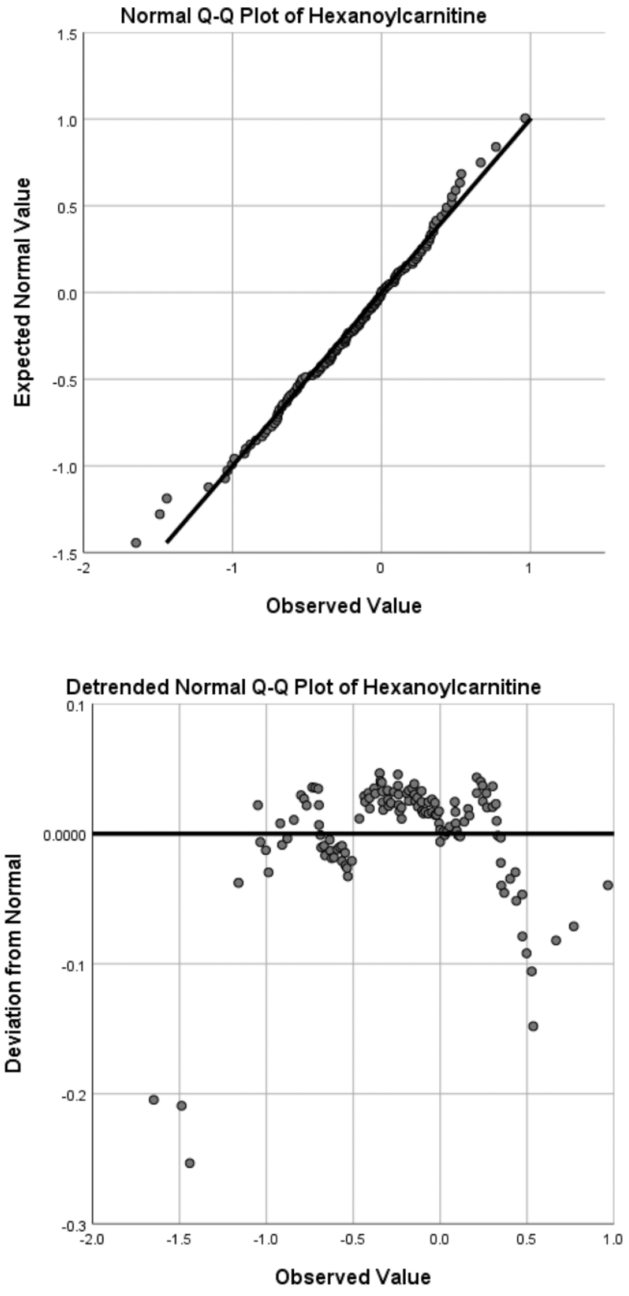

Supplementary Figure 35. The quantile-quantile (Q-Q) plot of normalized Hexanoylcarnitine which demonstrated the consistency of normalized value with expected normal value (upper panel) and the deviation of normalized values from expected normal value (lower panel).

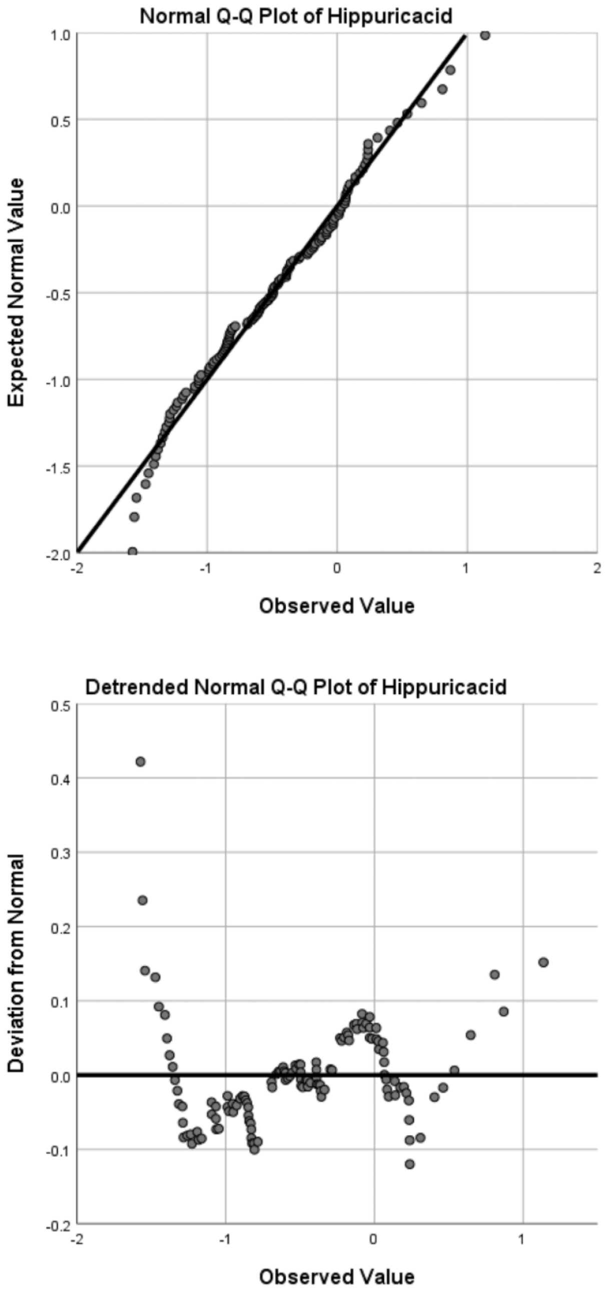

Supplementary Figure 36. The quantile-quantile (Q-Q) plot of normalized Hippuricacid which demonstrated the consistency of normalized value with expected normal value (upper panel) and the deviation of normalized values from expected normal value (lower panel).

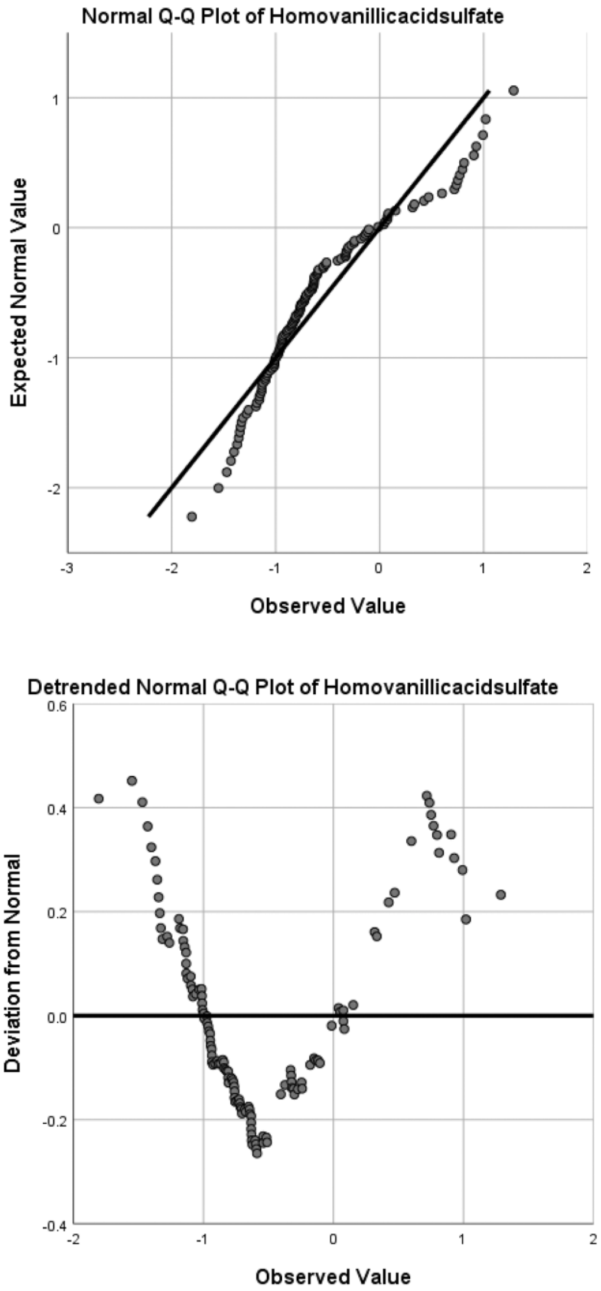

Supplementary Figure 37. The quantile-quantile (Q-Q) plot of normalized Homovanillicacidsulfate which demonstrated the consistency of normalized value with expected normal value (upper panel) and the deviation of normalized values from expected normal value (lower panel).

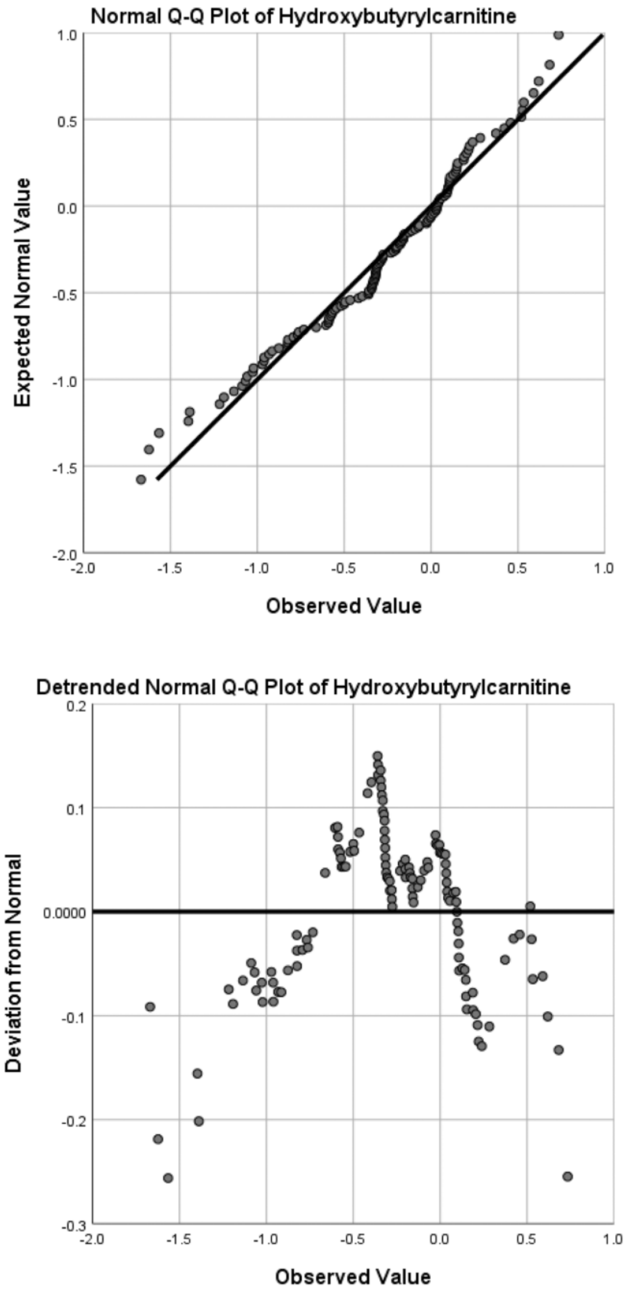

Supplementary Figure 38. The quantile-quantile (Q-Q) plot of normalized Hydroxybutyrylcarnitine which demonstrated the consistency of normalized value with expected normal value (upper panel) and the deviation of normalized values from expected normal value (lower panel).

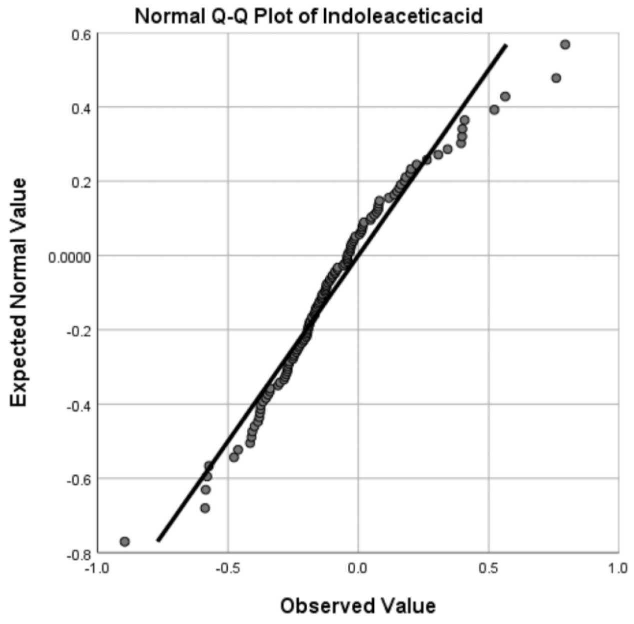

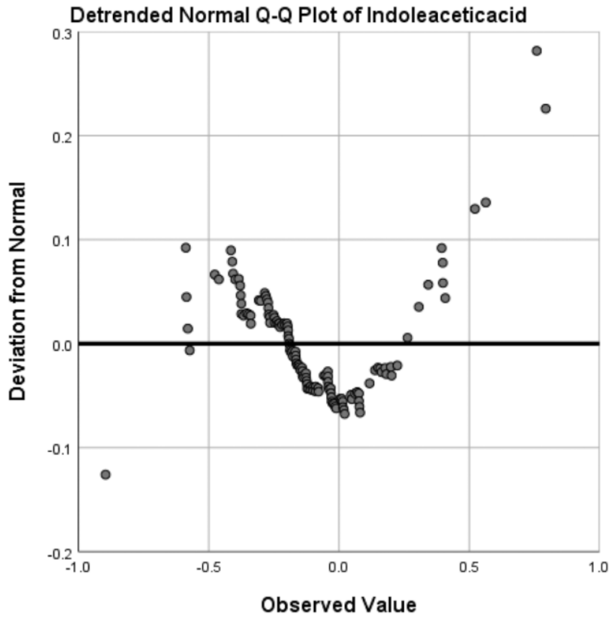

Supplementary Figure 39. The quantile-quantile (Q-Q) plot of normalized Indoleaceticacid which demonstrated the consistency of normalized value with expected normal value (upper panel) and the deviation of normalized values from expected normal value (lower panel).

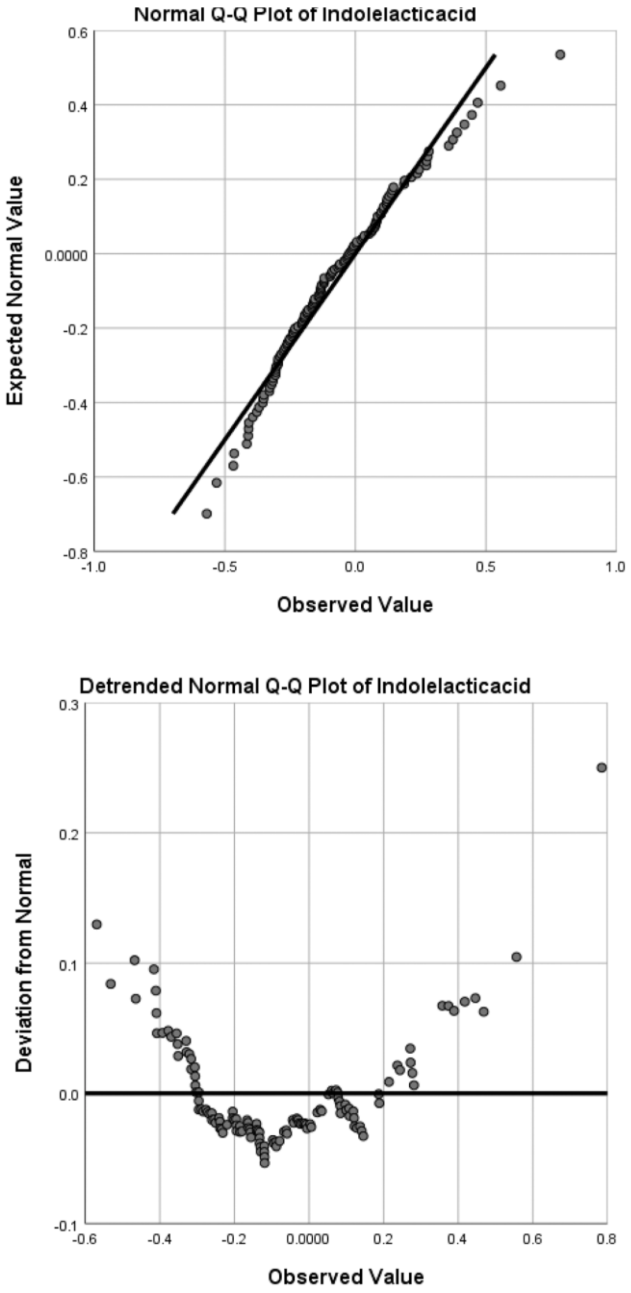

Supplementary Figure 40. The quantile-quantile (Q-Q) plot of normalized Indolelacticacid which demonstrated the consistency of normalized value with expected normal value (upper panel) and the deviation of normalized values from expected normal value (lower panel).

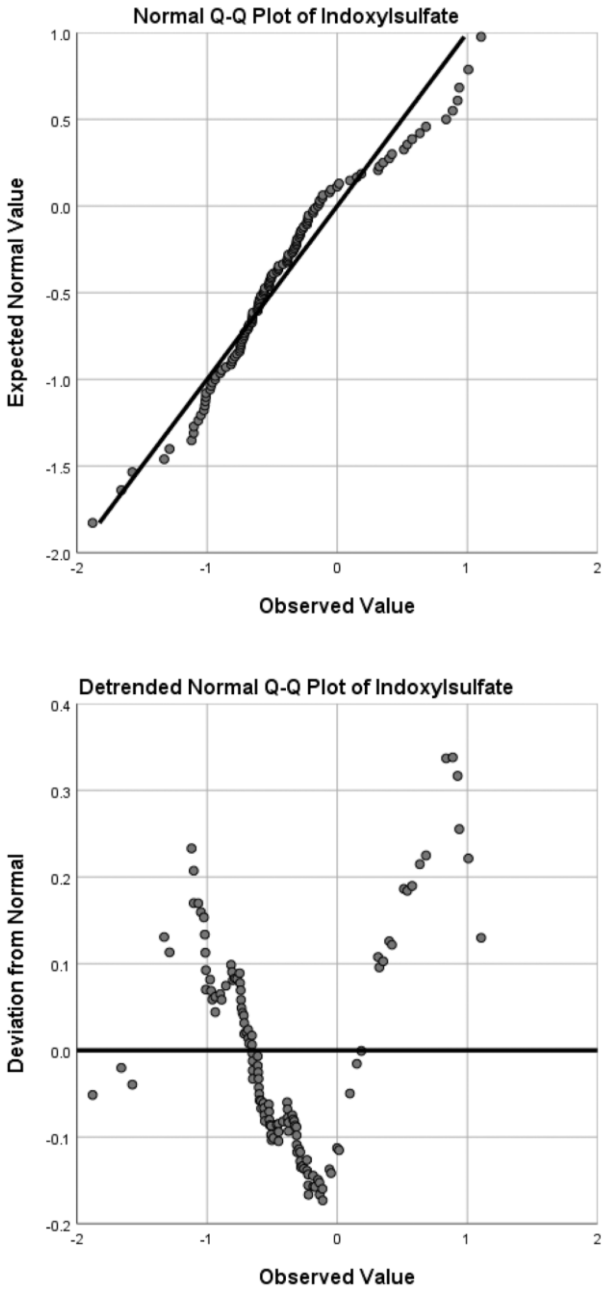

Supplementary Figure 41. The quantile-quantile (Q-Q) plot of normalized Indoxylsulfate which demonstrated the consistency of normalized value with expected normal value (upper panel) and the deviation of normalized values from expected normal value (lower panel).

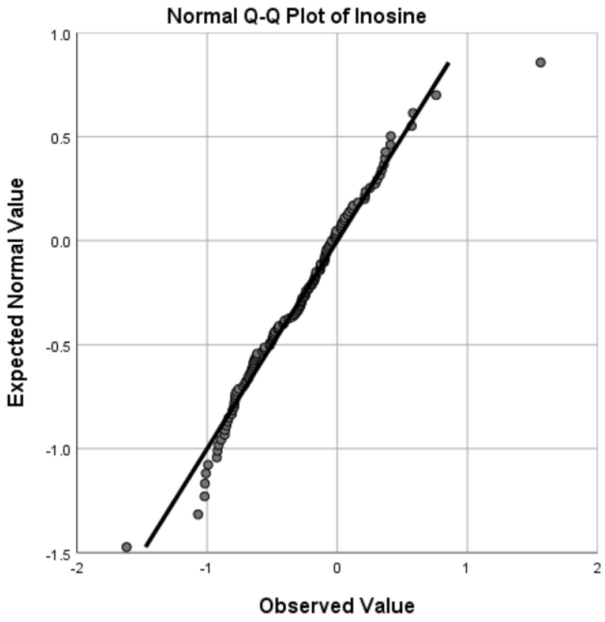

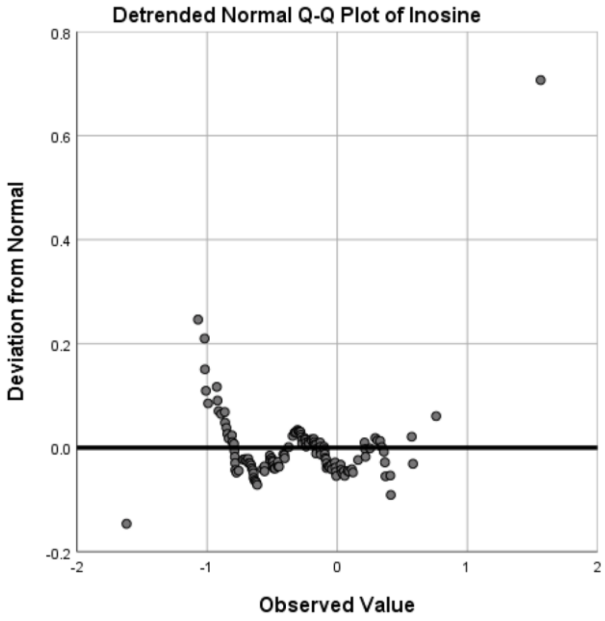

Supplementary Figure 42. The quantile-quantile (Q-Q) plot of normalized Inosine which demonstrated the consistency of normalized value with expected normal value (upper panel) and the deviation of normalized values from expected normal value (lower panel).

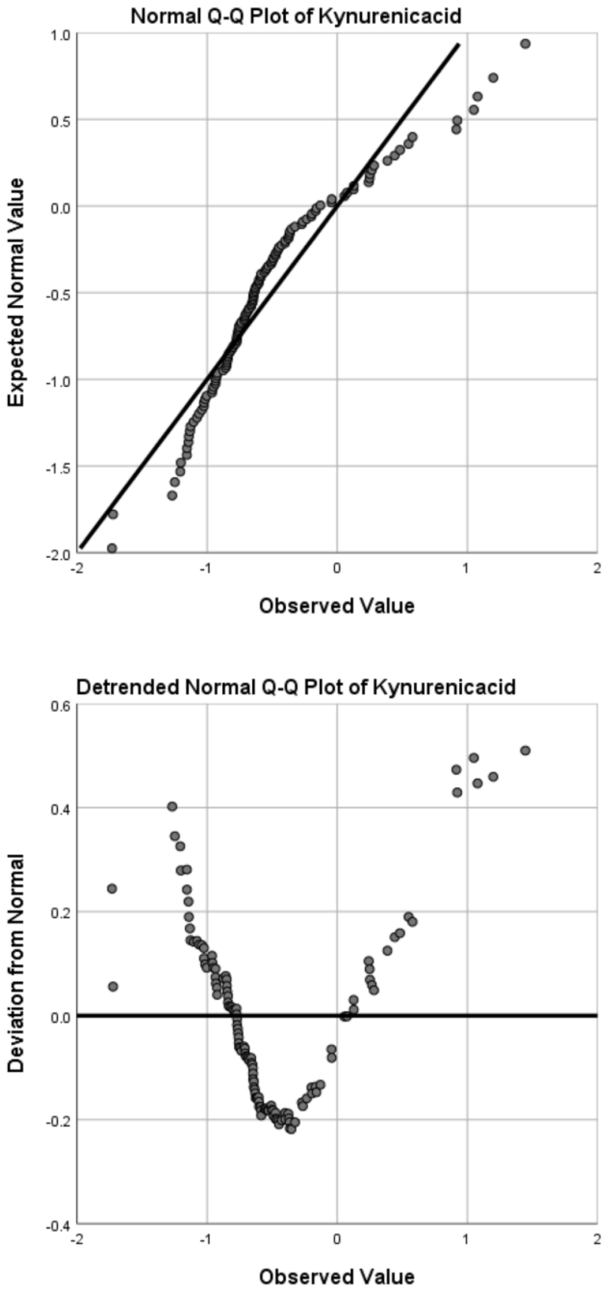

Supplementary Figure 43. The quantile-quantile (Q-Q) plot of normalized Kynurenicacid which demonstrated the consistency of normalized value with expected normal value (upper panel) and the deviation of normalized values from expected normal value (lower panel).

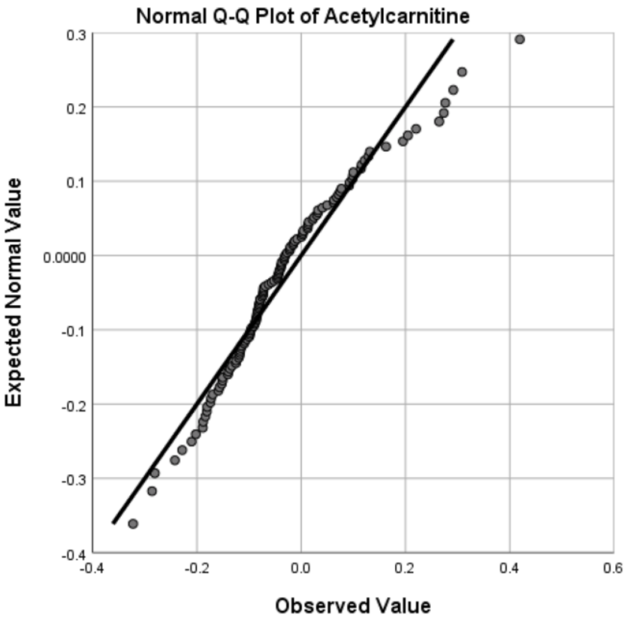

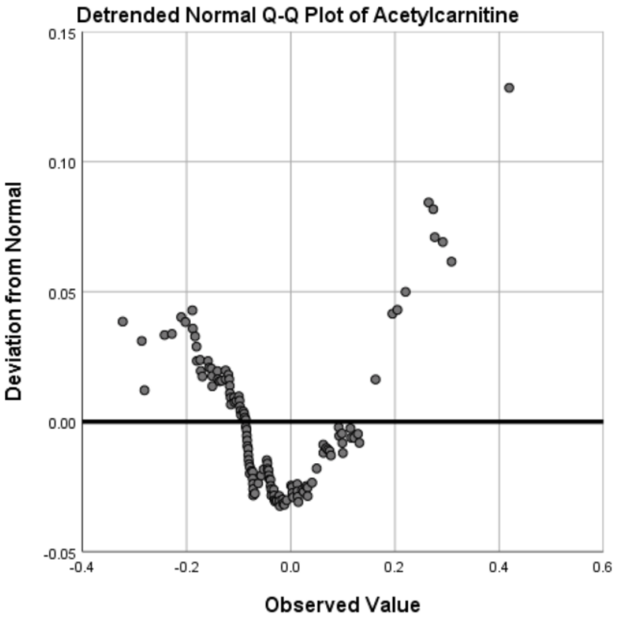

Supplementary Figure 44. The quantile-quantile (Q-Q) plot of normalized Acetylcarnitine which demonstrated the consistency of normalized value with expected normal value (upper panel) and the deviation of normalized values from expected normal value (lower panel).

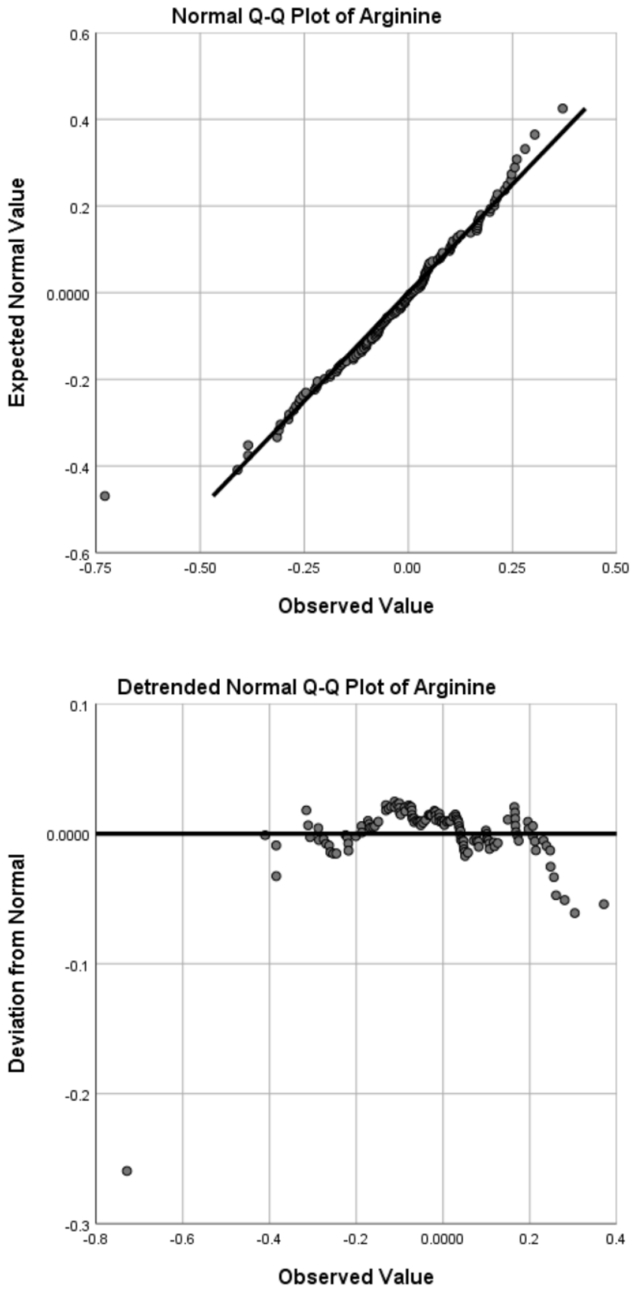

Supplementary Figure 45. The quantile-quantile (Q-Q) plot of normalized Arginine which demonstrated the consistency of normalized value with expected normal value (upper panel) and the deviation of normalized values from expected normal value (lower panel).

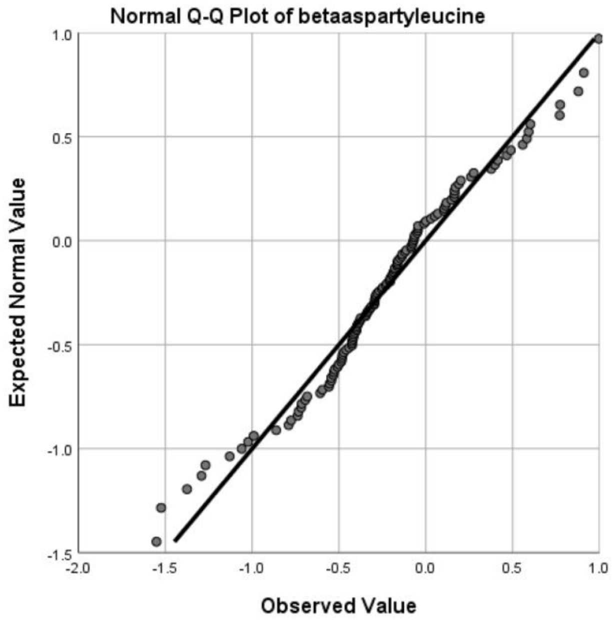

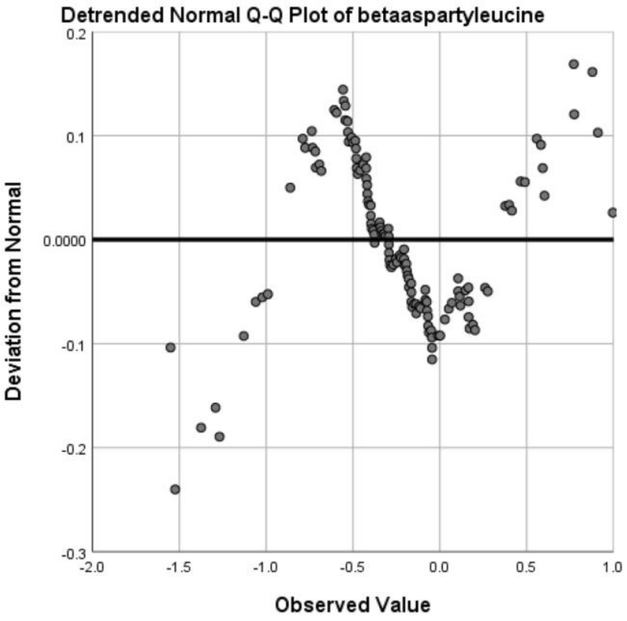

Supplementary Figure 46. The quantile-quantile (Q-Q) plot of normalized betaaspartyleucine which demonstrated the consistency of normalized value with expected normal value (upper panel) and the deviation of normalized values from expected normal value (lower panel).

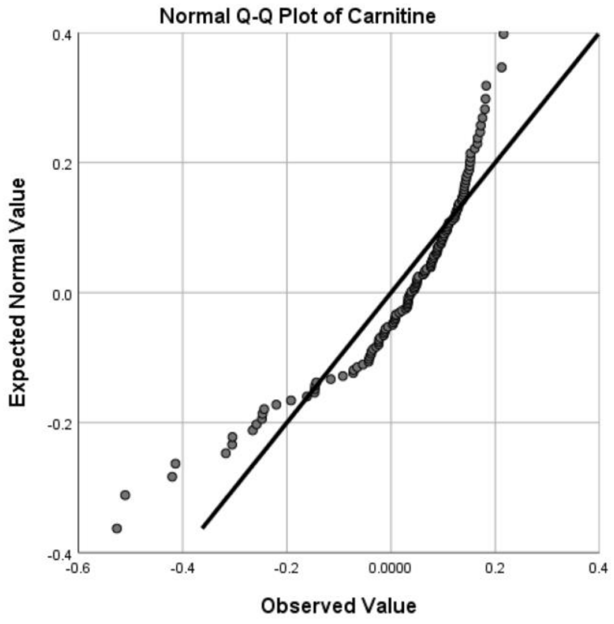

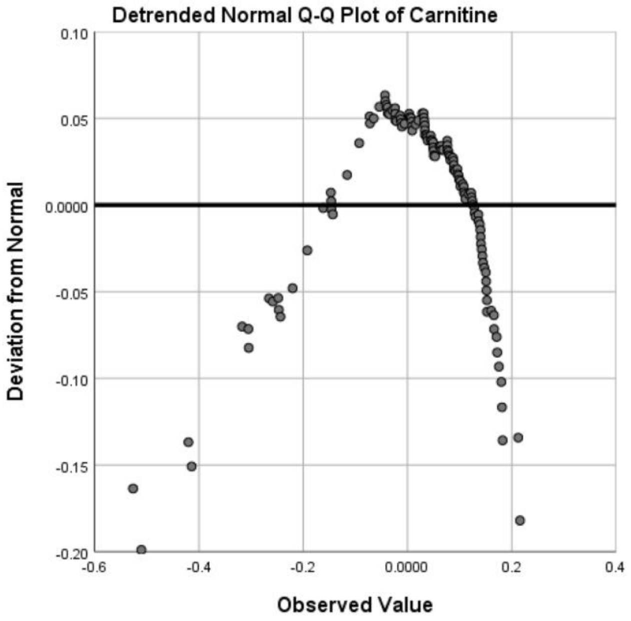

Supplementary Figure 47. The quantile-quantile (Q-Q) plot of normalized Carnitine which demonstrated the consistency of normalized value with expected normal value (upper panel) and the deviation of normalized values from expected normal value (lower panel).

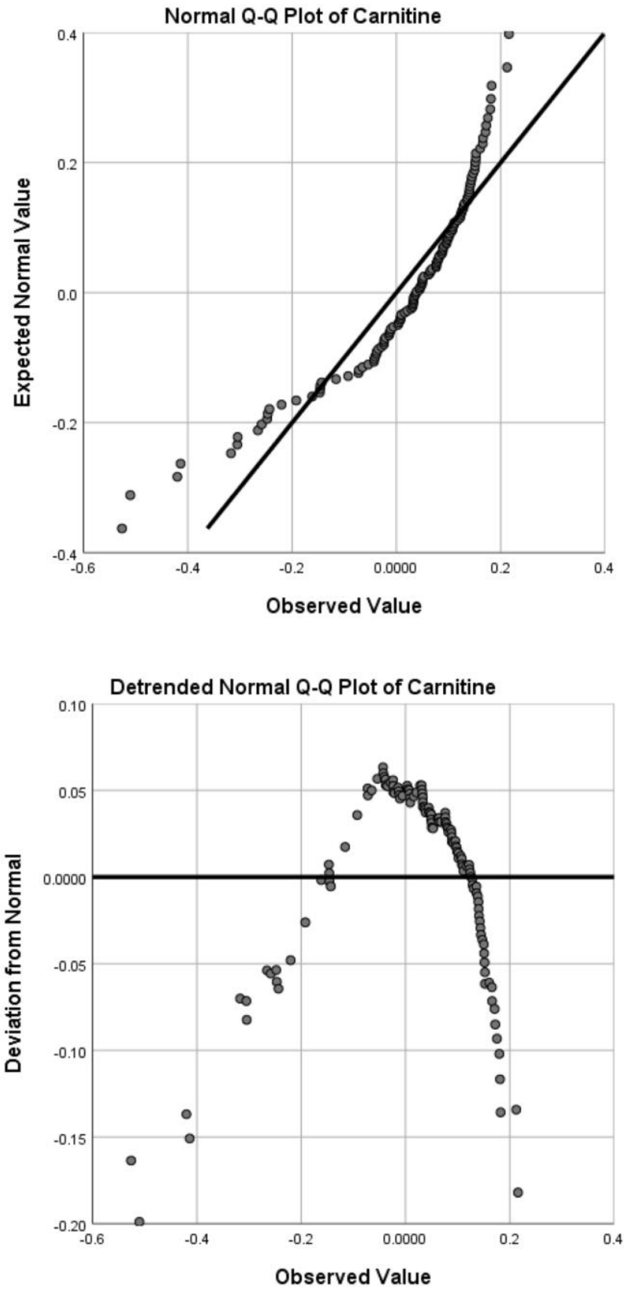

Supplementary Figure 48. The quantile-quantile (Q-Q) plot of normalized Glutamicacid which demonstrated the consistency of normalized value with expected normal value (upper panel) and the deviation of normalized values from expected normal value (lower panel).

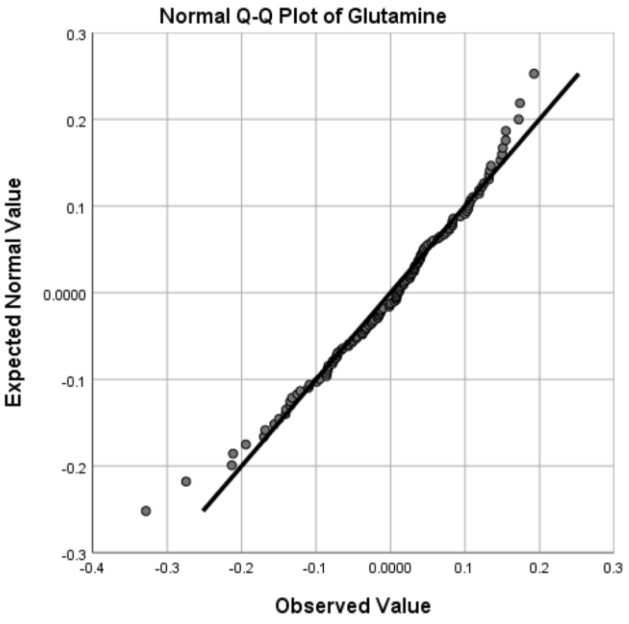

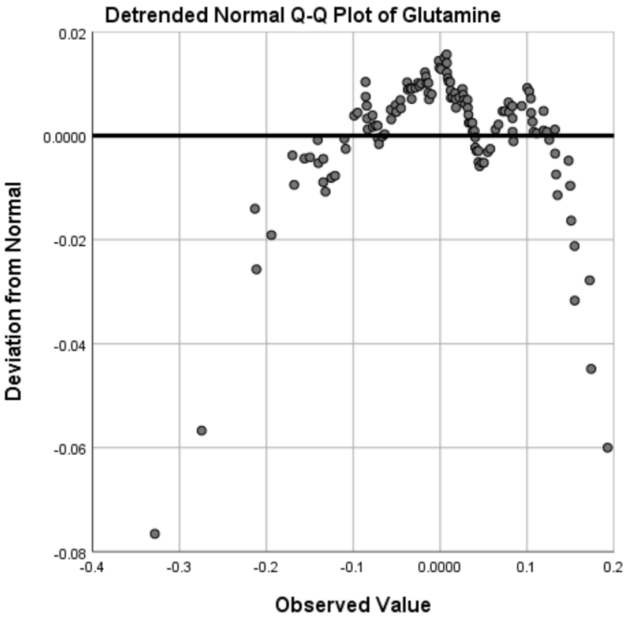

Supplementary Figure 49. The quantile-quantile (Q-Q) plot of normalized Glutamine which demonstrated the consistency of normalized value with expected normal value (upper panel) and the deviation of normalized values from expected normal value (lower panel).

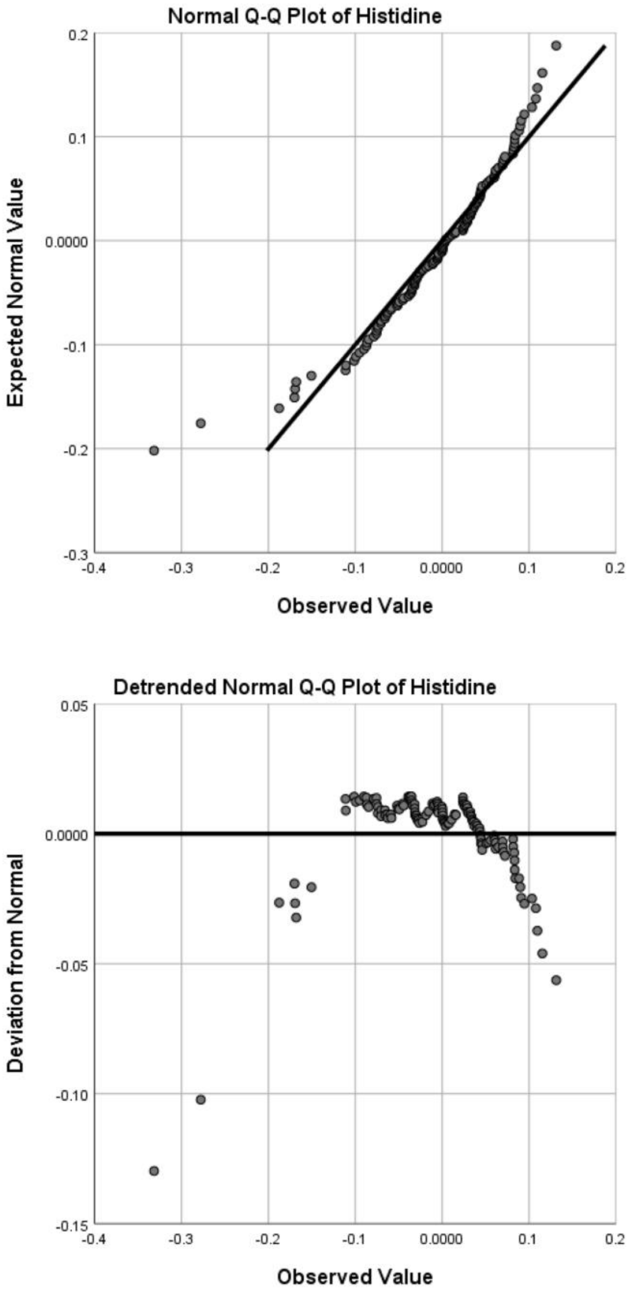

Supplementary Figure 50. The quantile-quantile (Q-Q) plot of normalized Histidine which demonstrated the consistency of normalized value with expected normal value (upper panel) and the deviation of normalized values from expected normal value (lower panel).

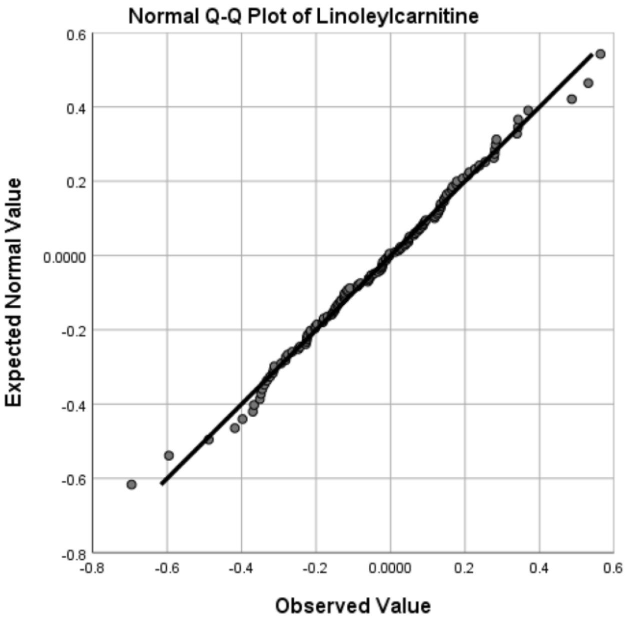

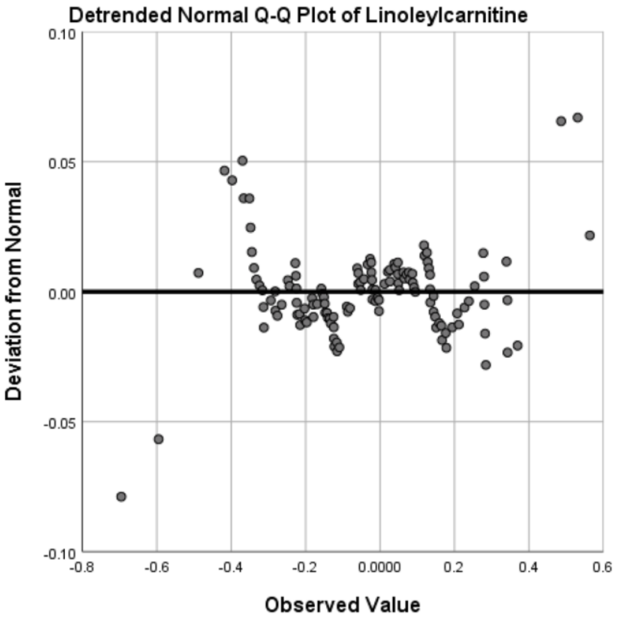

Supplementary Figure 51. The quantile-quantile (Q-Q) plot of normalized Linoleylcarnitine which demonstrated the consistency of normalized value with expected normal value (upper panel) and the deviation of normalized values from expected normal value (lower panel).

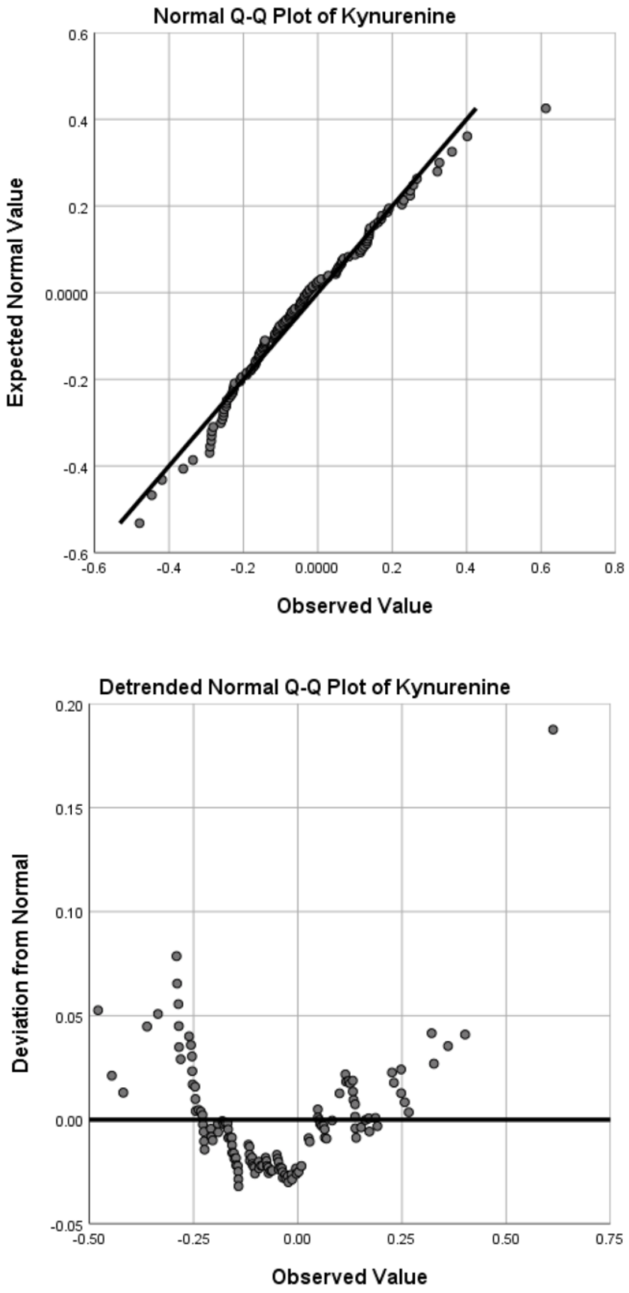

Supplementary Figure 52. The quantile-quantile (Q-Q) plot of normalized Kynurenine which demonstrated the consistency of normalized value with expected normal value (upper panel) and the deviation of normalized values from expected normal value (lower panel).

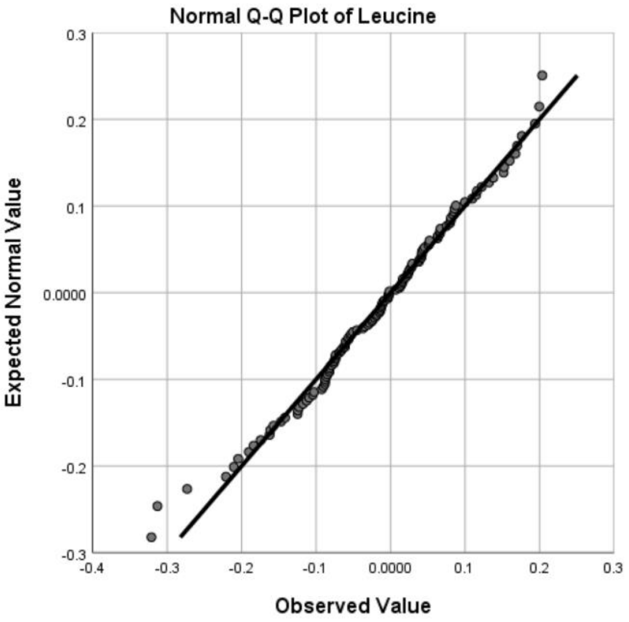

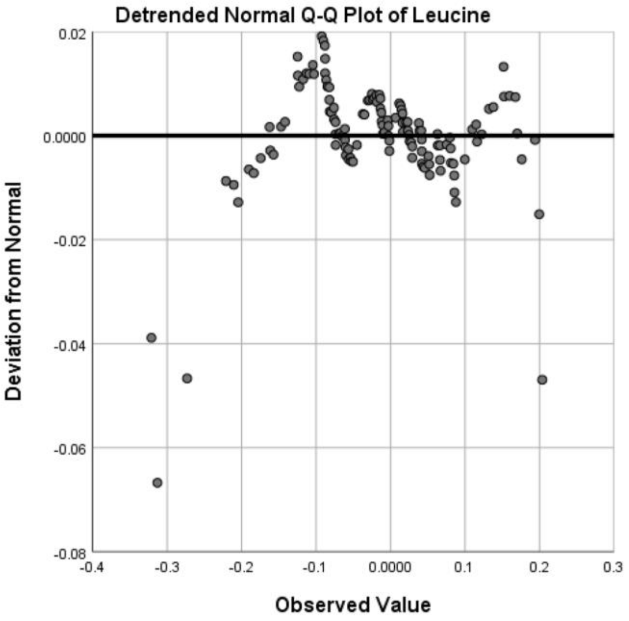

Supplementary Figure 53. The quantile-quantile (Q-Q) plot of normalized Leucine which demonstrated the consistency of normalized value with expected normal value (upper panel) and the deviation of normalized values from expected normal value (lower panel).

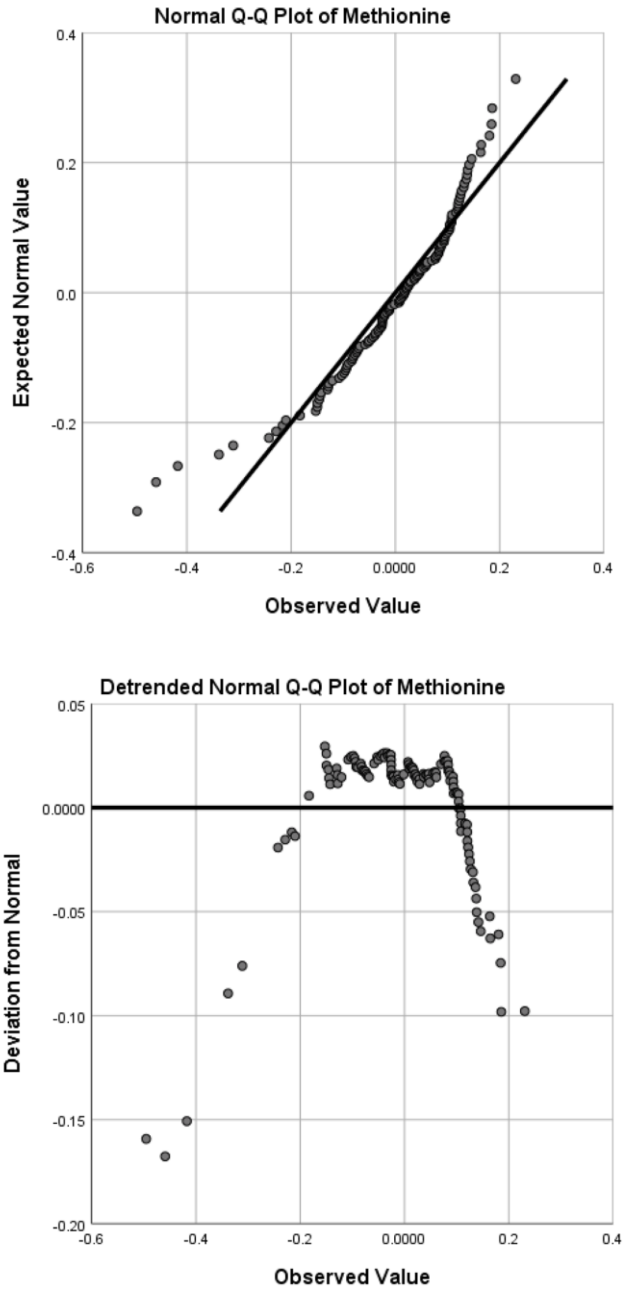

Supplementary Figure 54. The quantile-quantile (Q-Q) plot of normalized Methionine which demonstrated the consistency of normalized value with expected normal value (upper panel) and the deviation of normalized values from expected normal value (lower panel).

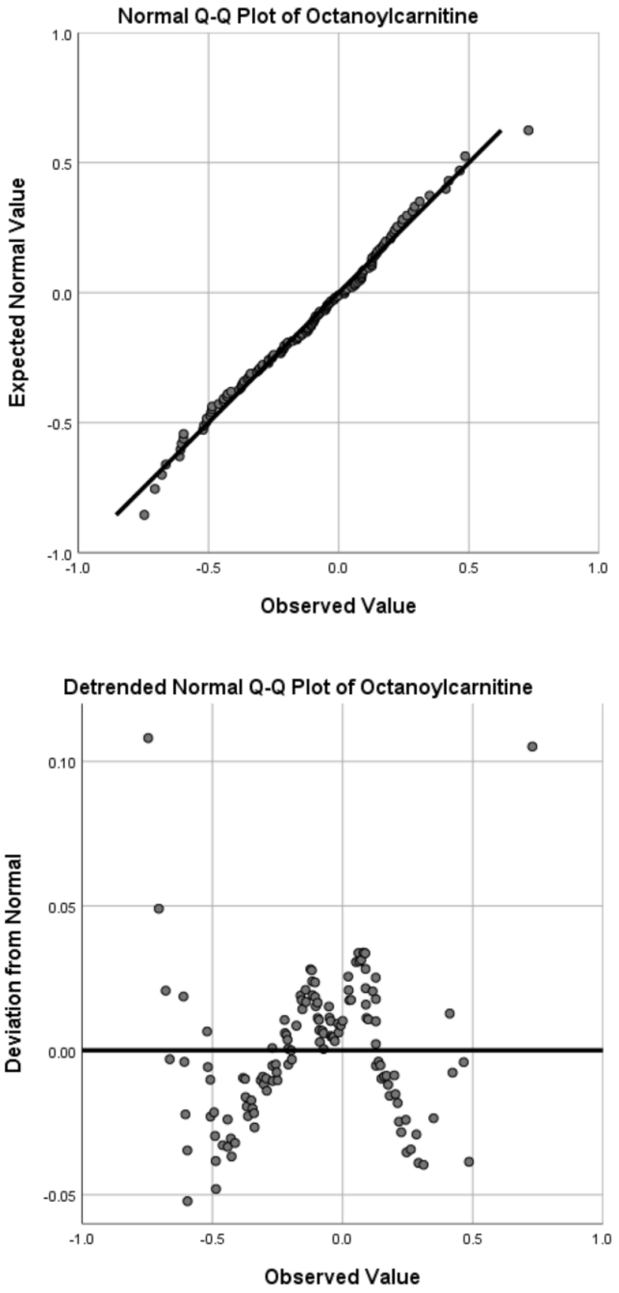

Supplementary Figure 55. The quantile-quantile (Q-Q) plot of normalized Octanoylcarnitine which demonstrated the consistency of normalized value with expected normal value (upper panel) and the deviation of normalized values from expected normal value (lower panel).

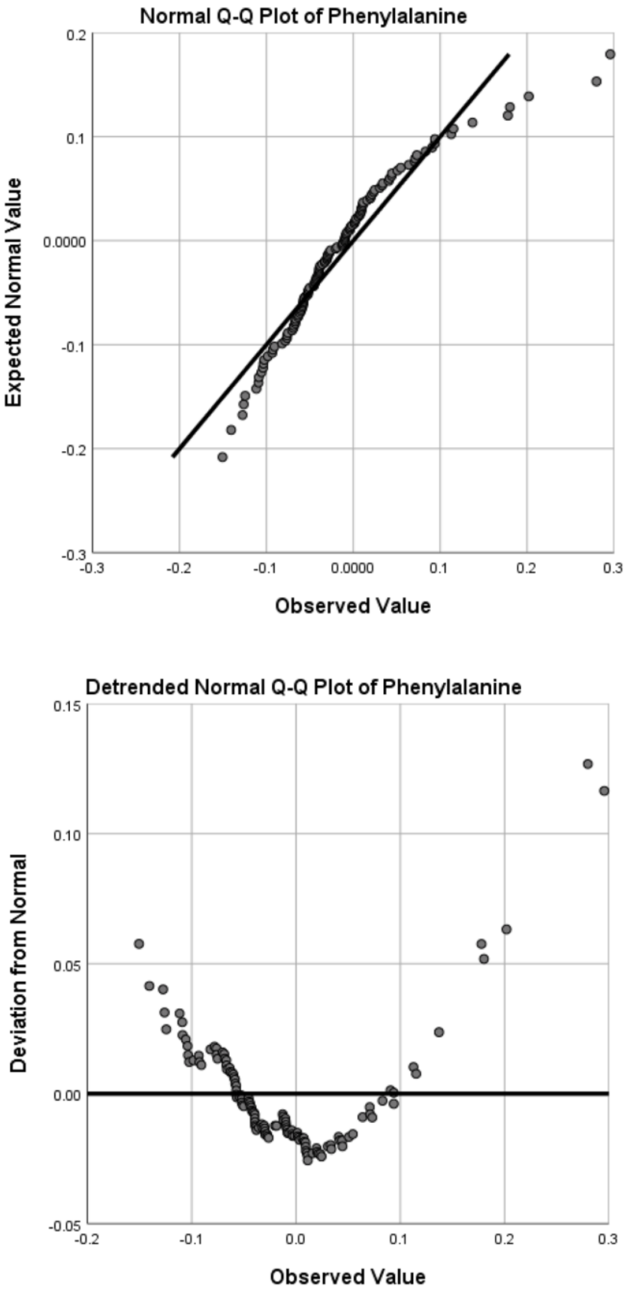

Supplementary Figure 56. The quantile-quantile (Q-Q) plot of normalized Phenylalanine which demonstrated the consistency of normalized value with expected normal value (upper panel) and the deviation of normalized values from expected normal value (lower panel).

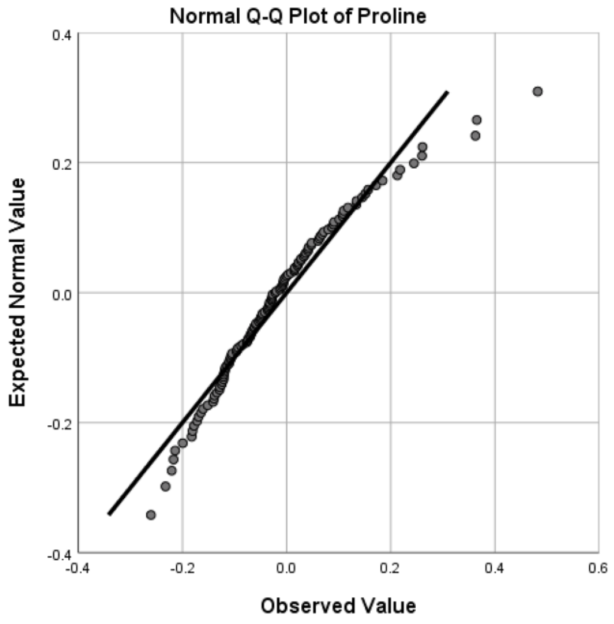

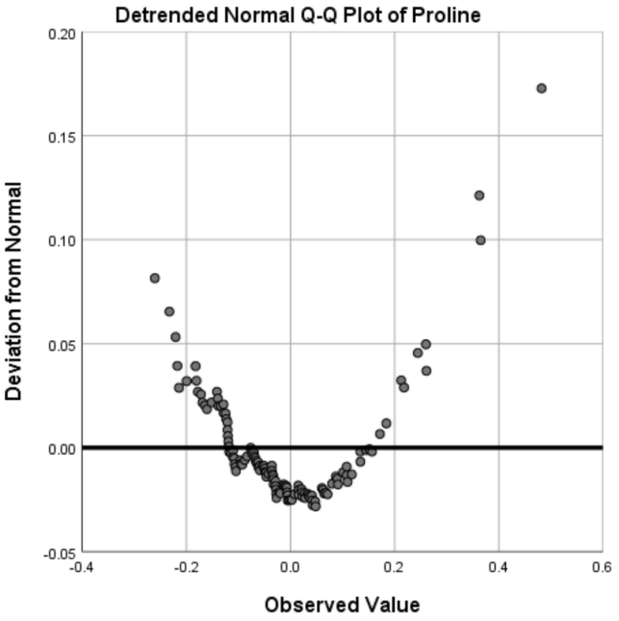

Supplementary Figure 57. The quantile-quantile (Q-Q) plot of normalized Proline which demonstrated the consistency of normalized value with expected normal value (upper panel) and the deviation of normalized values from expected normal value (lower panel).

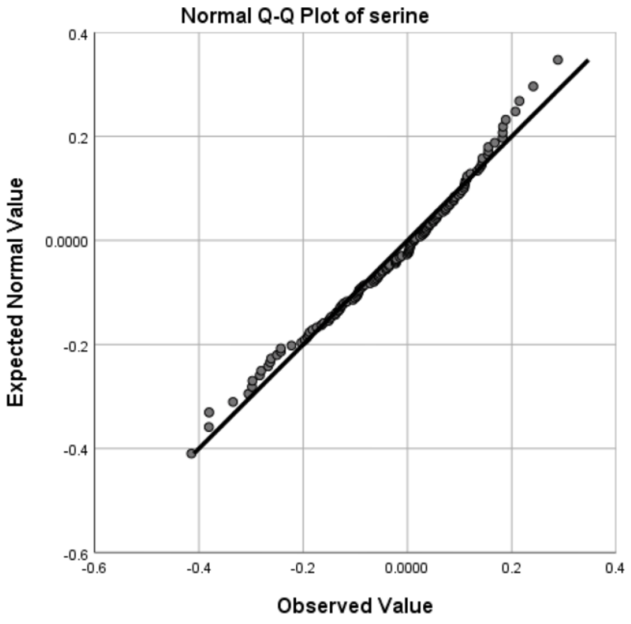

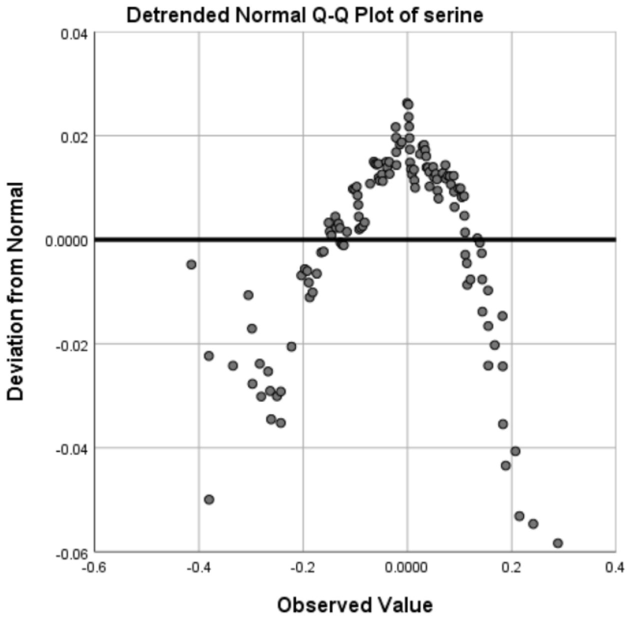

Supplementary Figure 58. The quantile-quantile (Q-Q) plot of normalized serine which demonstrated the consistency of normalized value with expected normal value (upper panel) and the deviation of normalized values from expected normal value (lower panel).

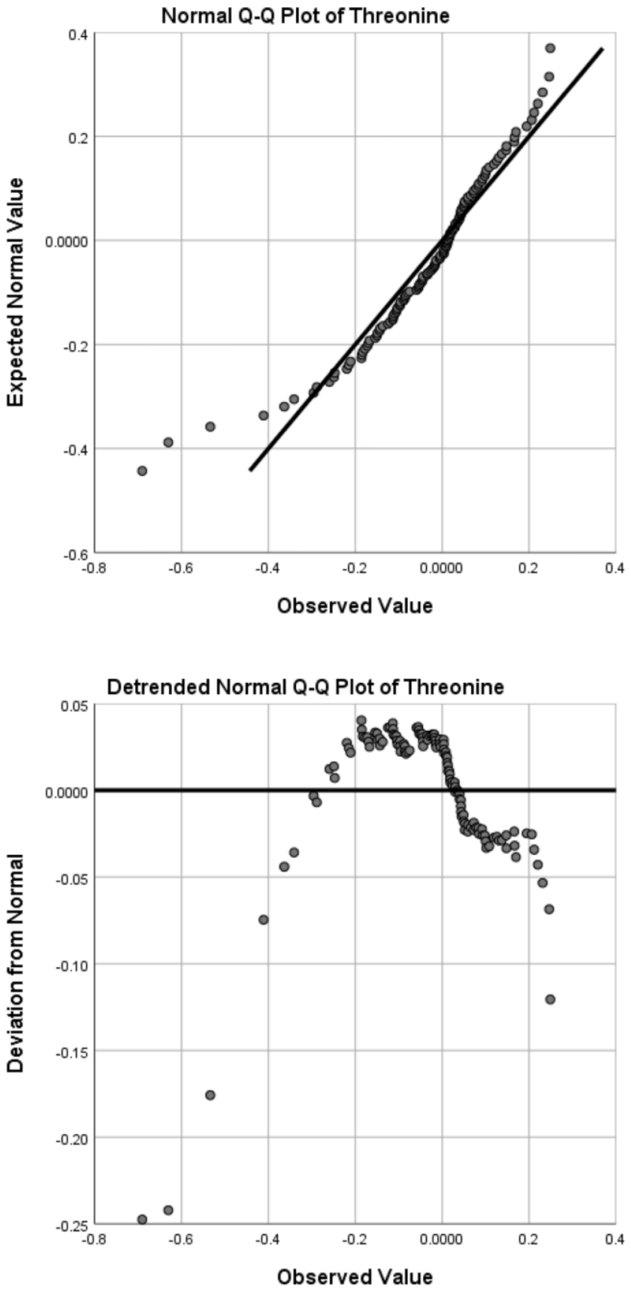

Supplementary Figure 59. The quantile-quantile (Q-Q) plot of normalized Threonine which demonstrated the consistency of normalized value with expected normal value (upper panel) and the deviation of normalized values from expected normal value (lower panel).

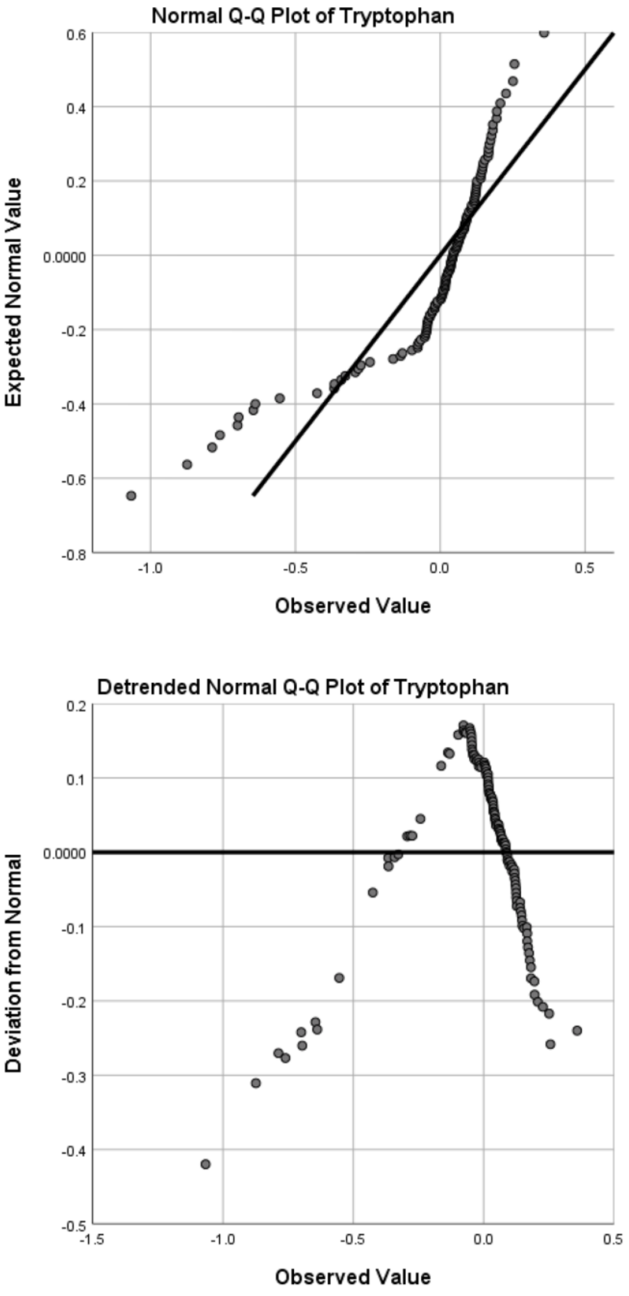

Supplementary Figure 60. The quantile-quantile (Q-Q) plot of normalized Tryptophan which demonstrated the consistency of normalized value with expected normal value (upper panel) and the deviation of normalized values from expected normal value (lower panel).

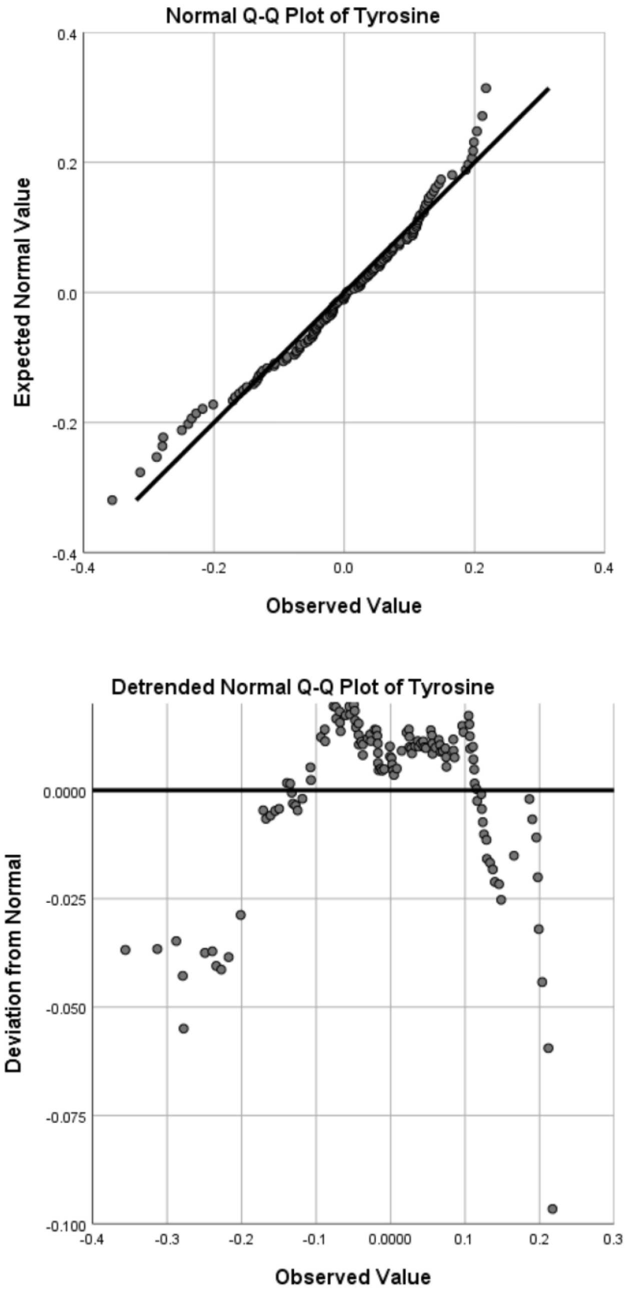

Supplementary Figure 61. The quantile-quantile (Q-Q) plot of normalized Tyrosine which demonstrated the consistency of normalized value with expected normal value (upper panel) and the deviation of normalized values from expected normal value (lower panel).

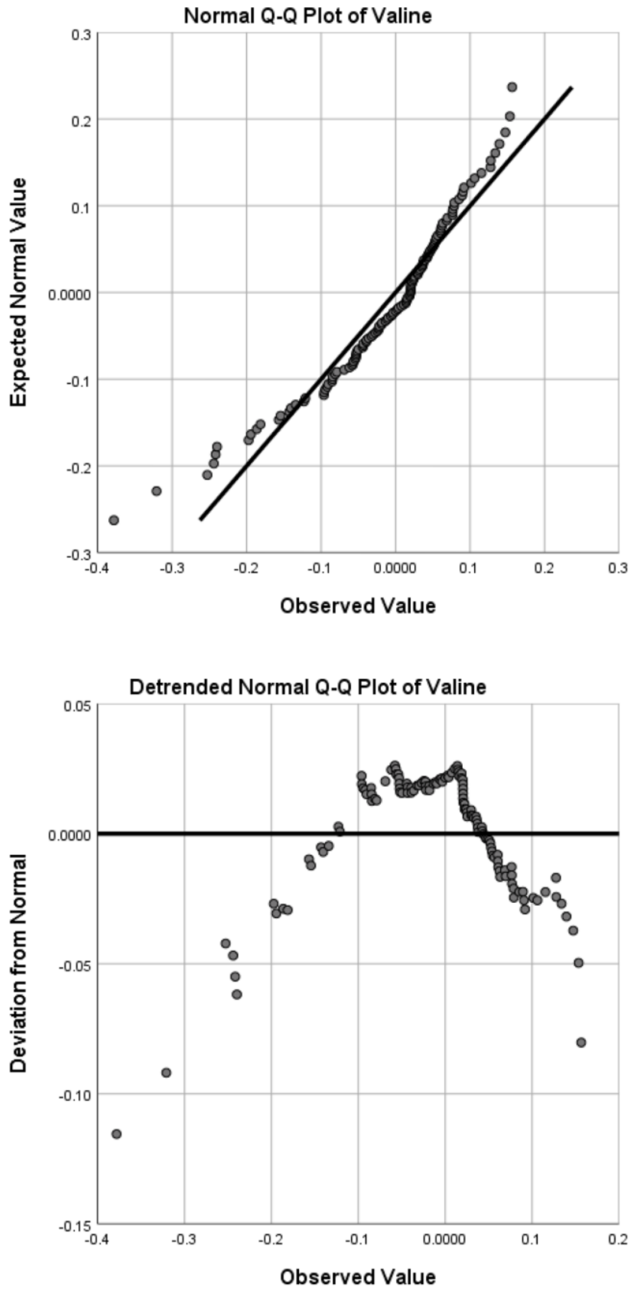

Supplementary Figure 62. The quantile-quantile (Q-Q) plot of normalized Valine which demonstrated the consistency of normalized value with expected normal value (upper panel) and the deviation of normalized values from expected normal value (lower panel).

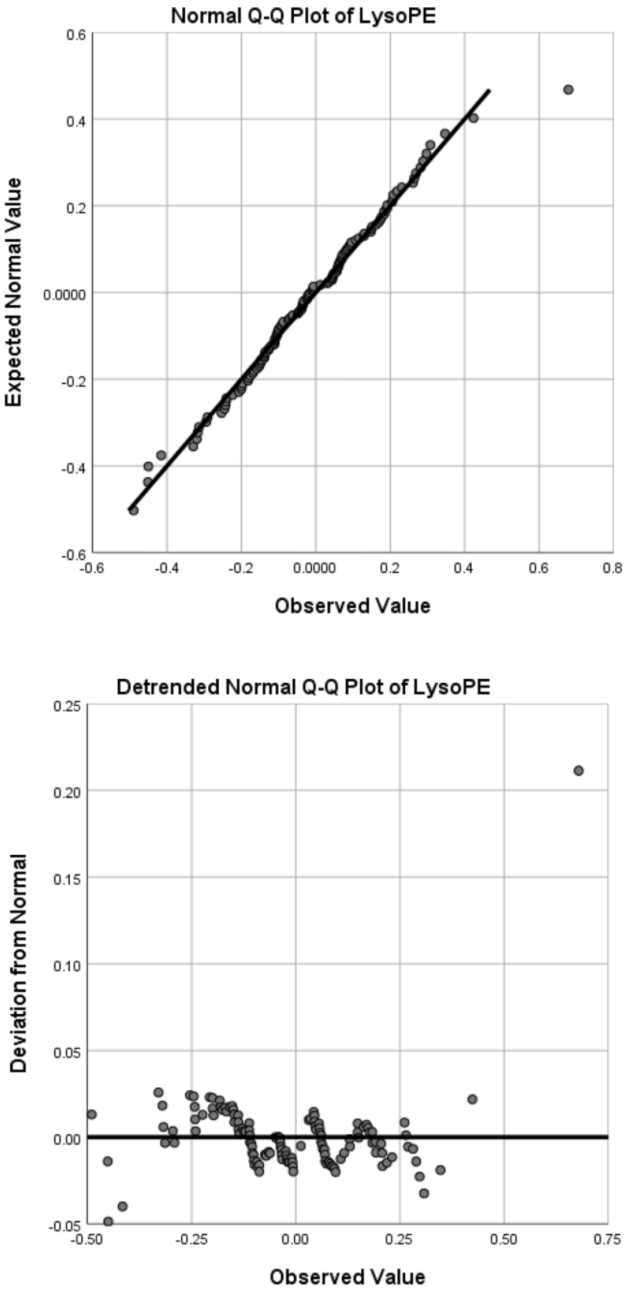

Supplementary Figure 63. The quantile-quantile (Q-Q) plot of normalized LysoPE which demonstrated the consistency of normalized value with expected normal value (upper panel) and the deviation of normalized values from expected normal value (lower panel).

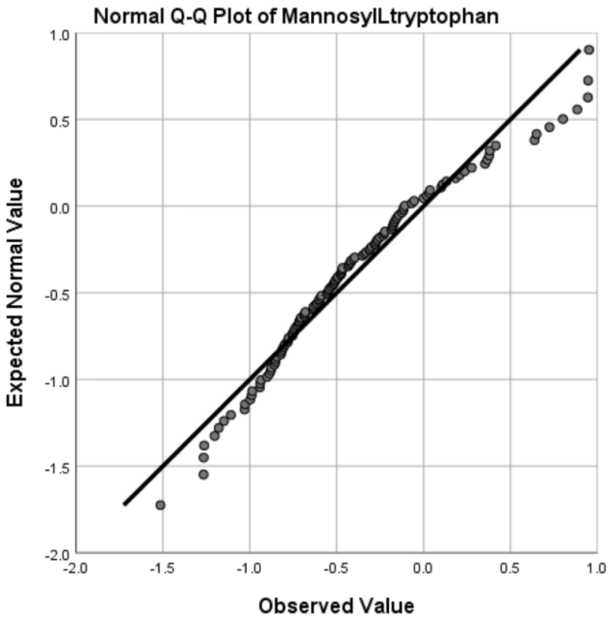

Supplementary Figure 64. The quantile-quantile (Q-Q) plot of normalized MannosylLtryptophan which demonstrated the consistency of normalized value with expected normal value (upper panel) and the deviation of normalized values from expected normal value (lower panel).

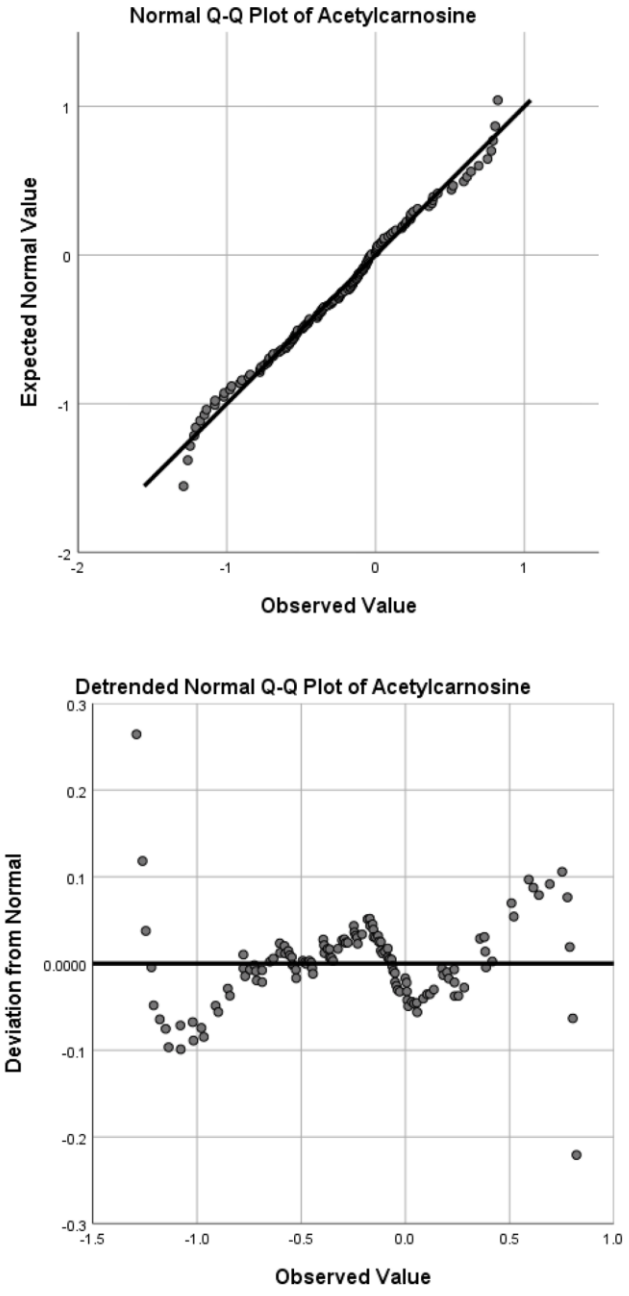

Supplementary Figure 65. The quantile-quantile (Q-Q) plot of normalized Acetylcarnosine which demonstrated the consistency of normalized value with expected normal value (upper panel) and the deviation of normalized values from expected normal value (lower panel).

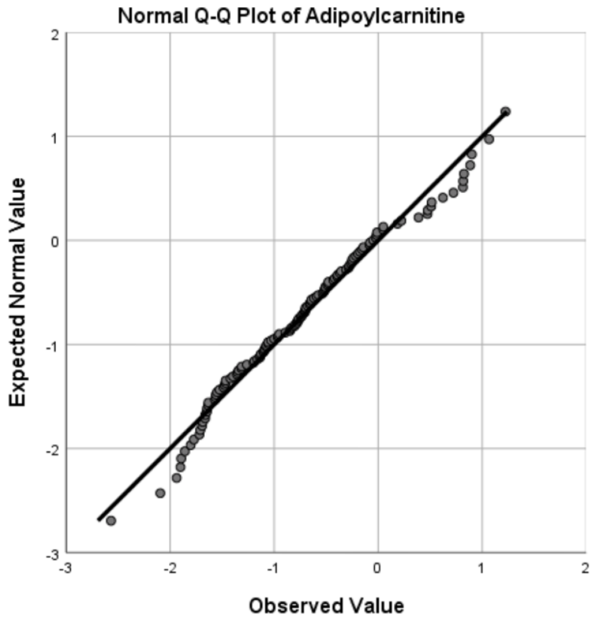

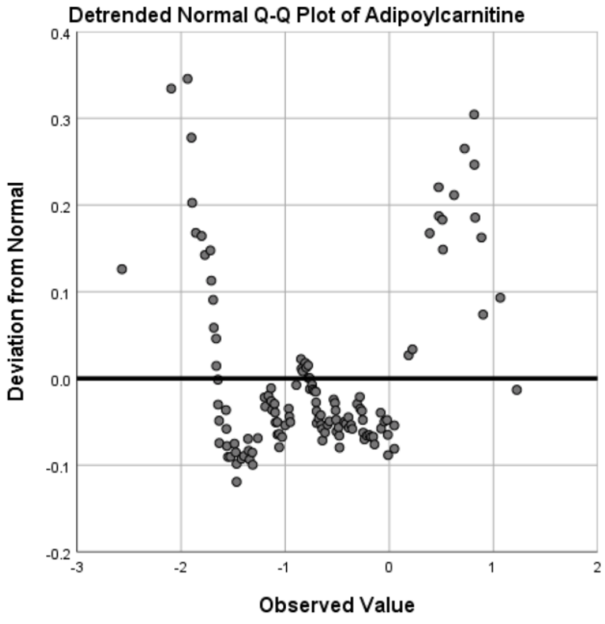

Supplementary Figure 66. The quantile-quantile (Q-Q) plot of normalized Adipoylcarnitine which demonstrated the consistency of normalized value with expected normal value (upper panel) and the deviation of normalized values from expected normal value (lower panel).

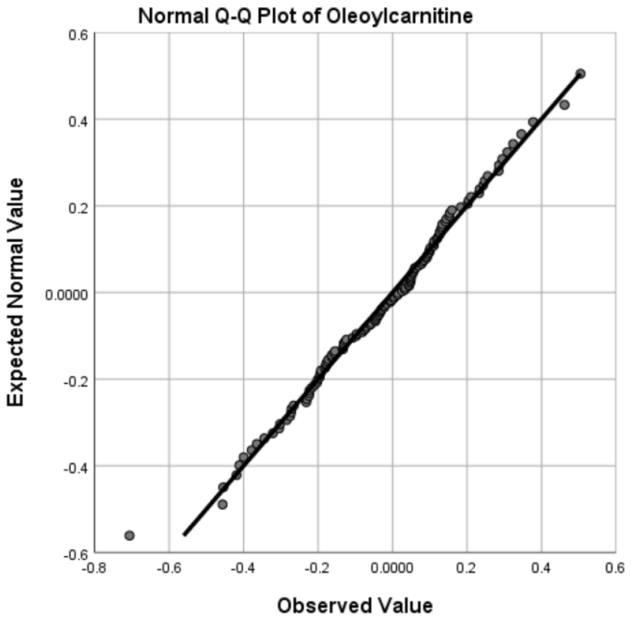

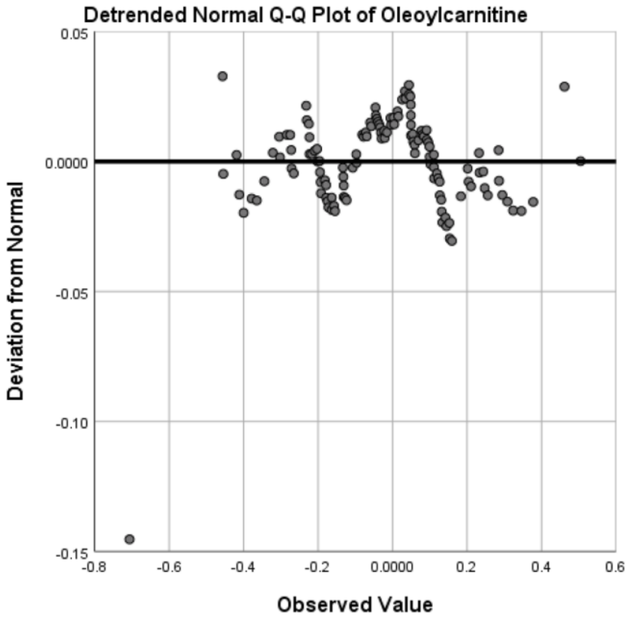

Supplementary Figure 67. The quantile-quantile (Q-Q) plot of normalized Oleoylcarnitine which demonstrated the consistency of normalized value with expected normal value (upper panel) and the deviation of normalized values from expected normal value (lower panel).

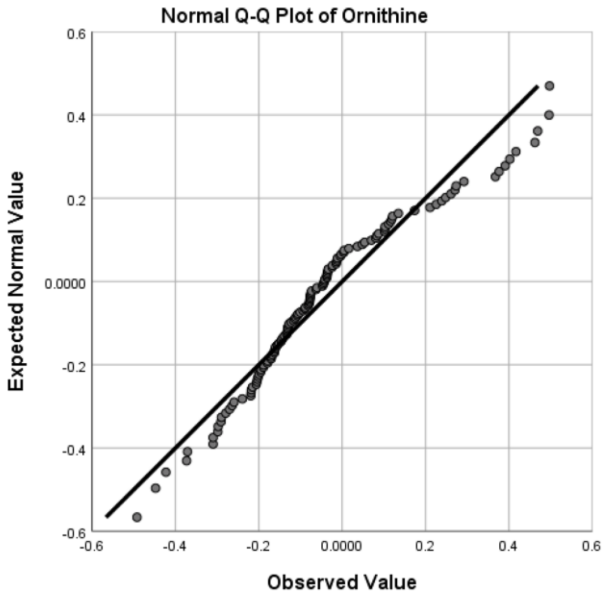

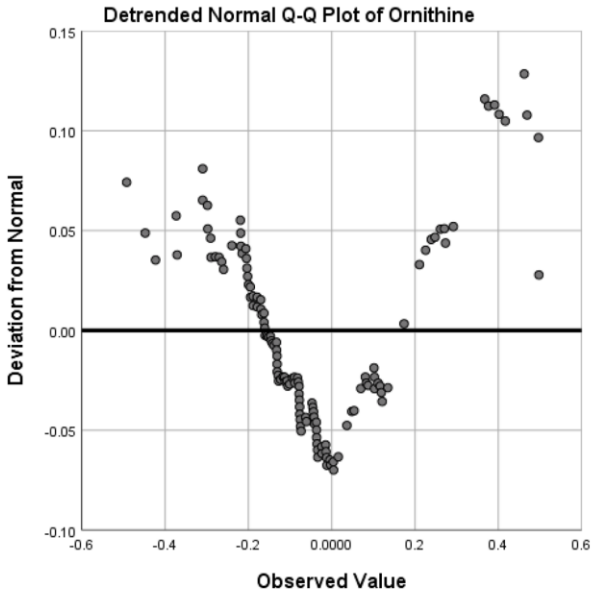

Supplementary Figure 68. The quantile-quantile (Q-Q) plot of normalized Ornithine which demonstrated the consistency of normalized value with expected normal value (upper panel) and the deviation of normalized values from expected normal value (lower panel).

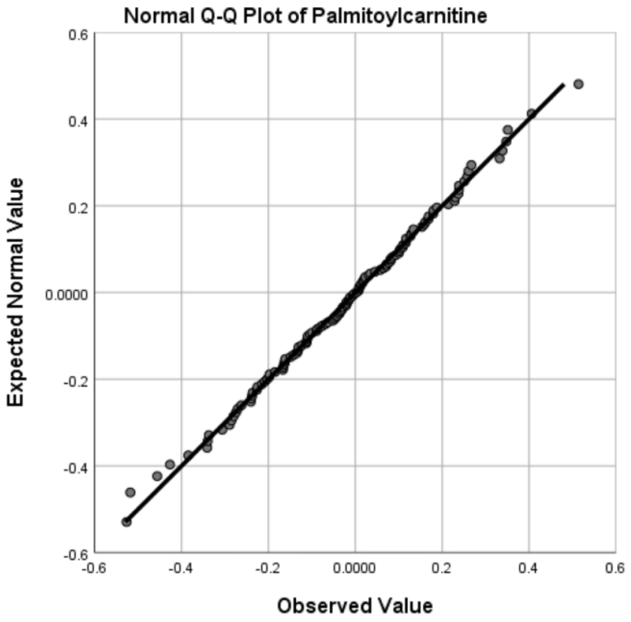

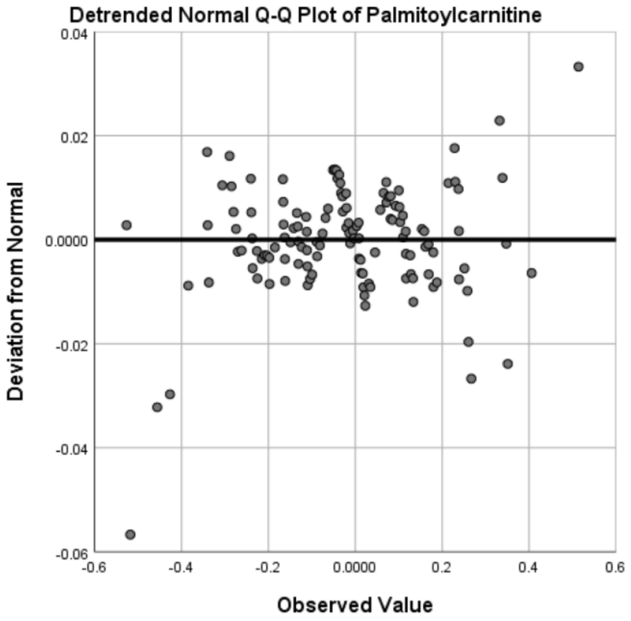

Supplementary Figure 69. The quantile-quantile (Q-Q) plot of normalized Palmitoylcarnitine which demonstrated the consistency of normalized value with expected normal value (upper panel) and the deviation of normalized values from expected normal value (lower panel).

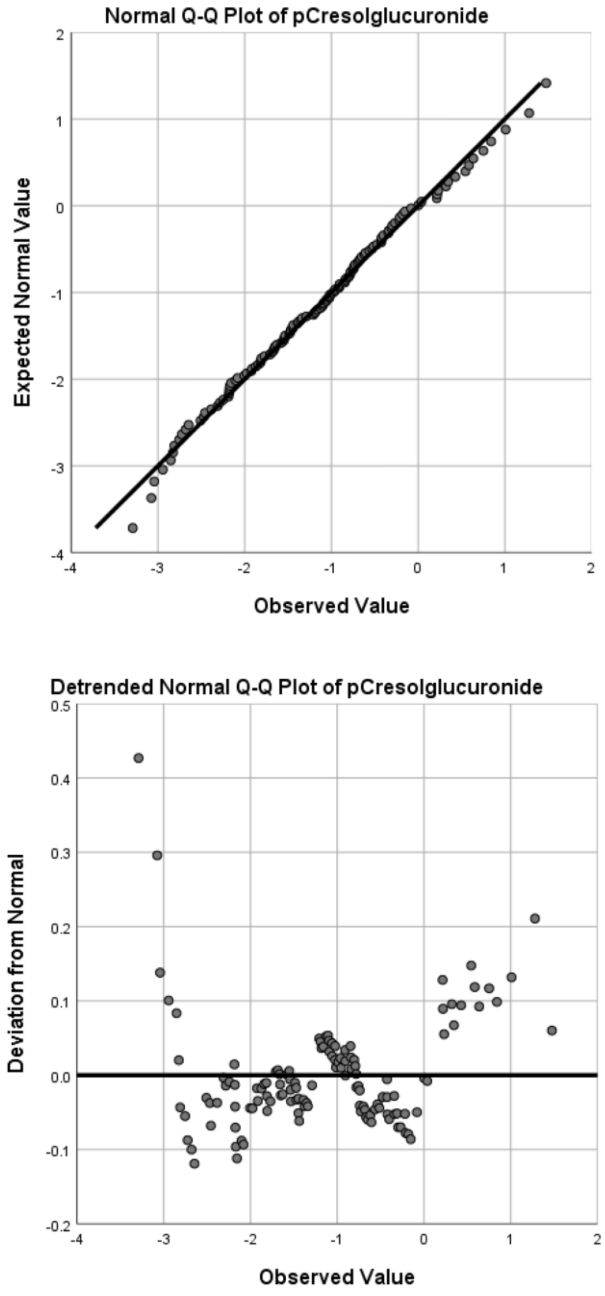

Supplementary Figure 70. The quantile-quantile (Q-Q) plot of normalized pCresolglucuronide which demonstrated the consistency of normalized value with expected normal value (upper panel) and the deviation of normalized values from expected normal value (lower panel).

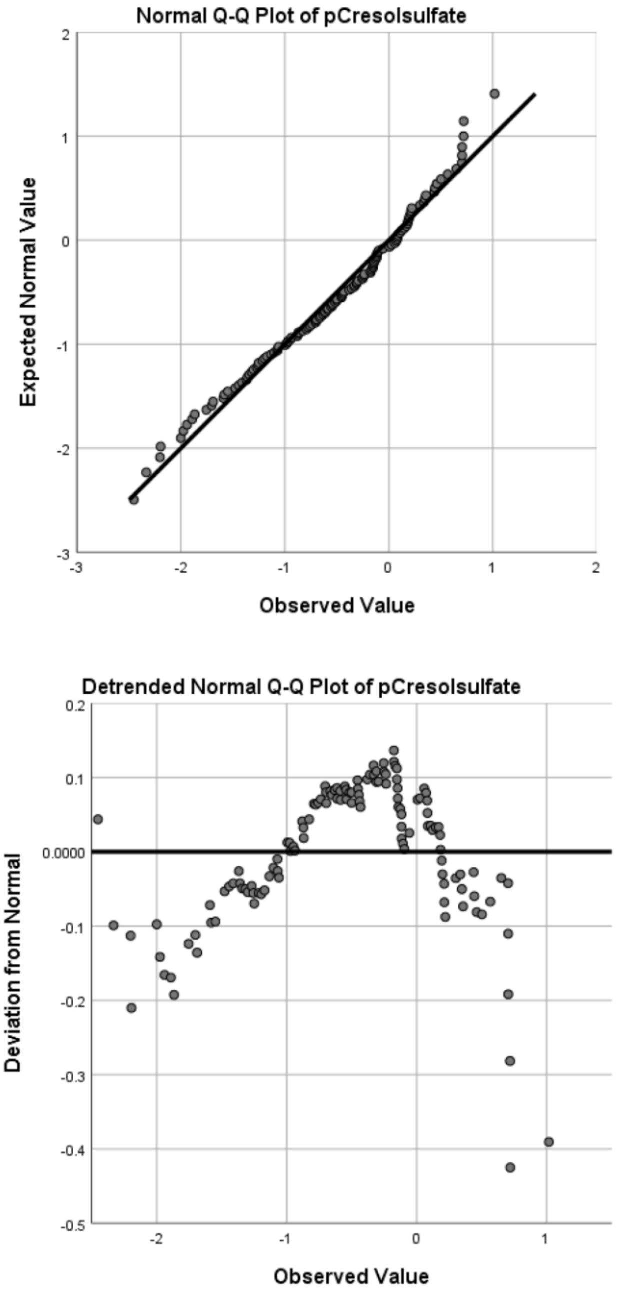

Supplementary Figure 71. The quantile-quantile (Q-Q) plot of normalized pCresolsulfate which demonstrated the consistency of normalized value with expected normal value (upper panel) and the deviation of normalized values from expected normal value (lower panel).

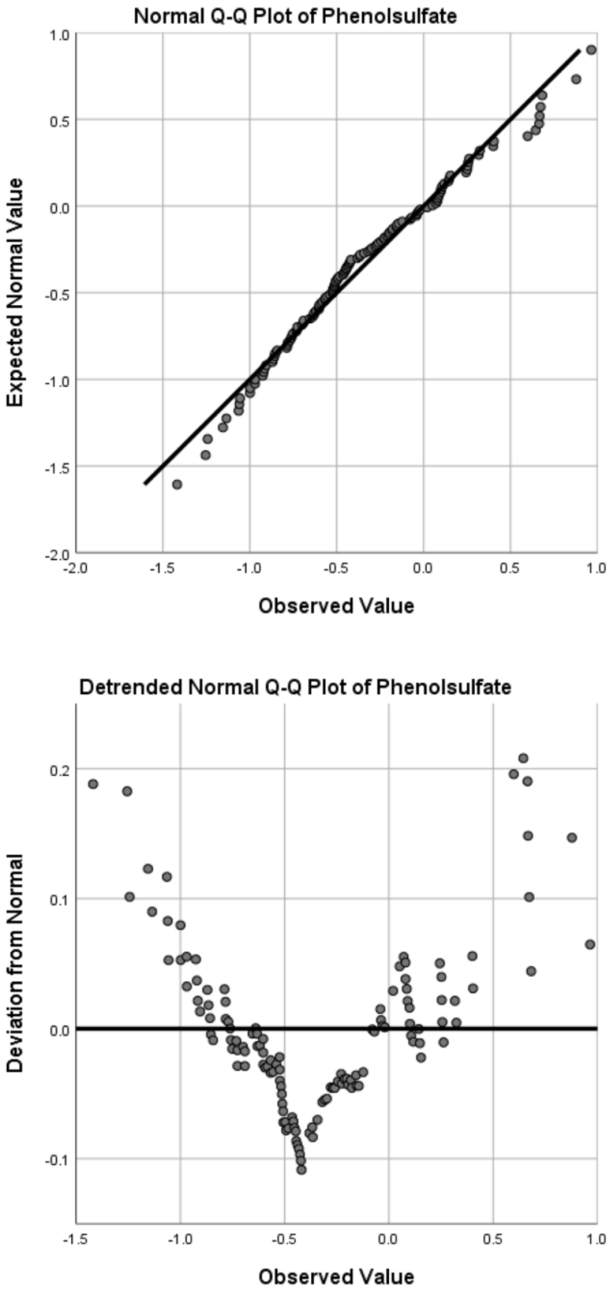

Supplementary Figure 72. The quantile-quantile (Q-Q) plot of normalized Phenolsulfate which demonstrated the consistency of normalized value with expected normal value (upper panel) and the deviation of normalized values from expected normal value (lower panel).

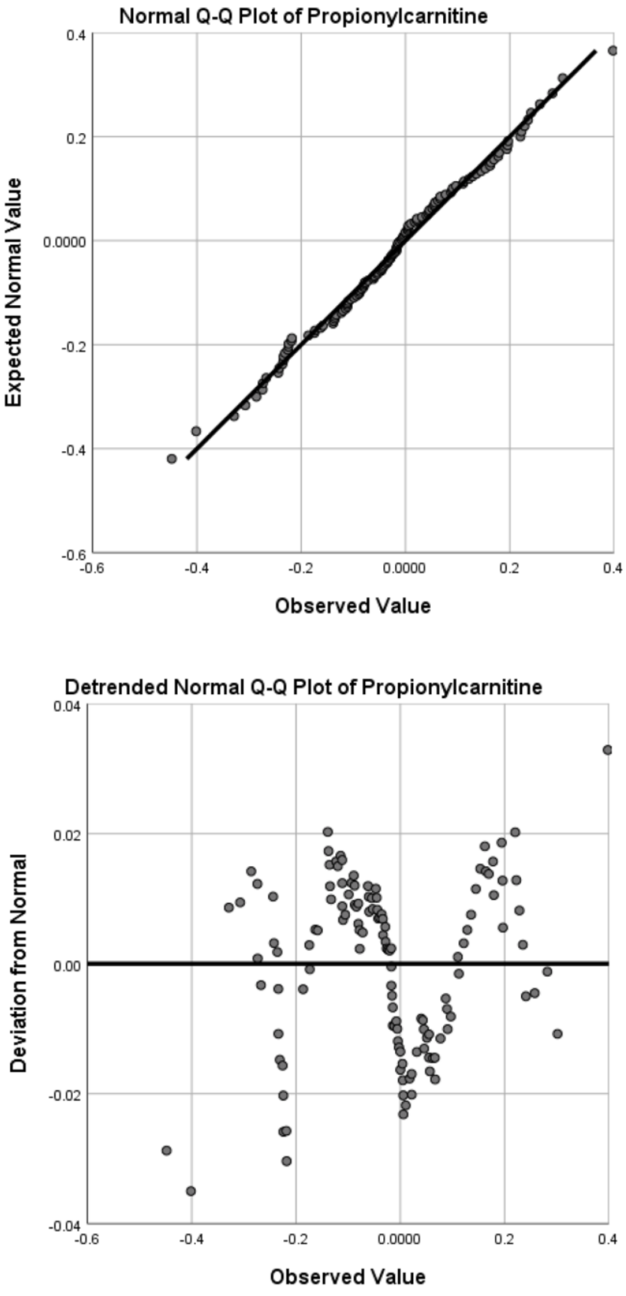

Supplementary Figure 73. The quantile-quantile (Q-Q) plot of normalized Propionylcarnitine which demonstrated the consistency of normalized value with expected normal value (upper panel) and the deviation of normalized values from expected normal value (lower panel).

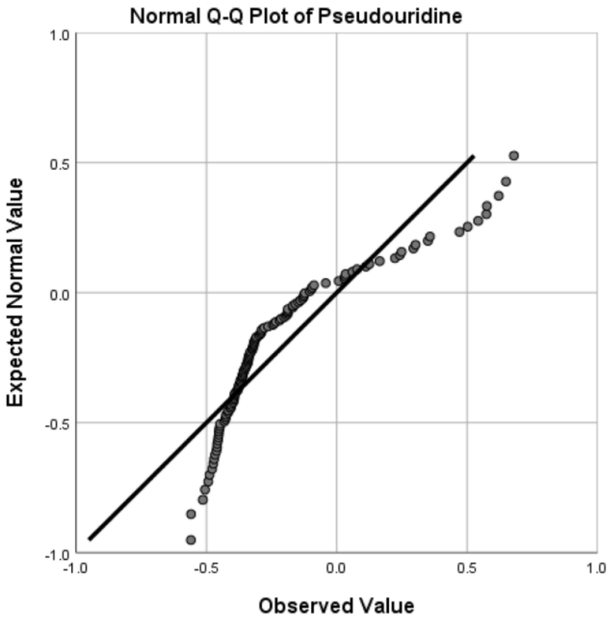

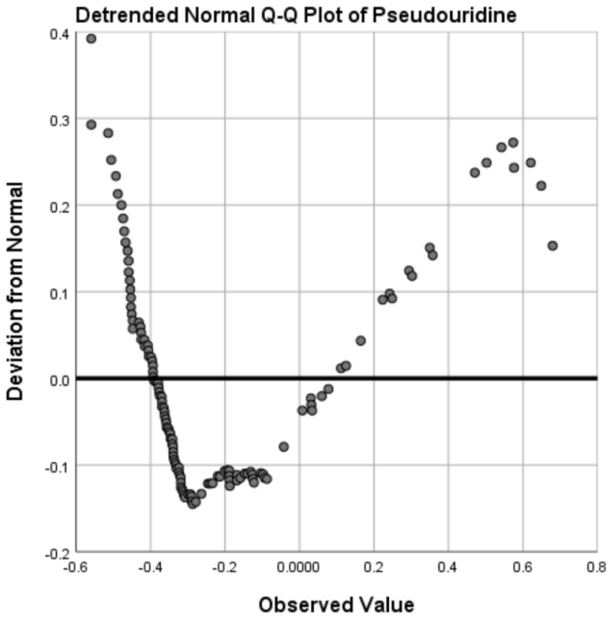

Supplementary Figure 74. The quantile-quantile (Q-Q) plot of normalized Pseudouridine which demonstrated the consistency of normalized value with expected normal value (upper panel) and the deviation of normalized values from expected normal value (lower panel).

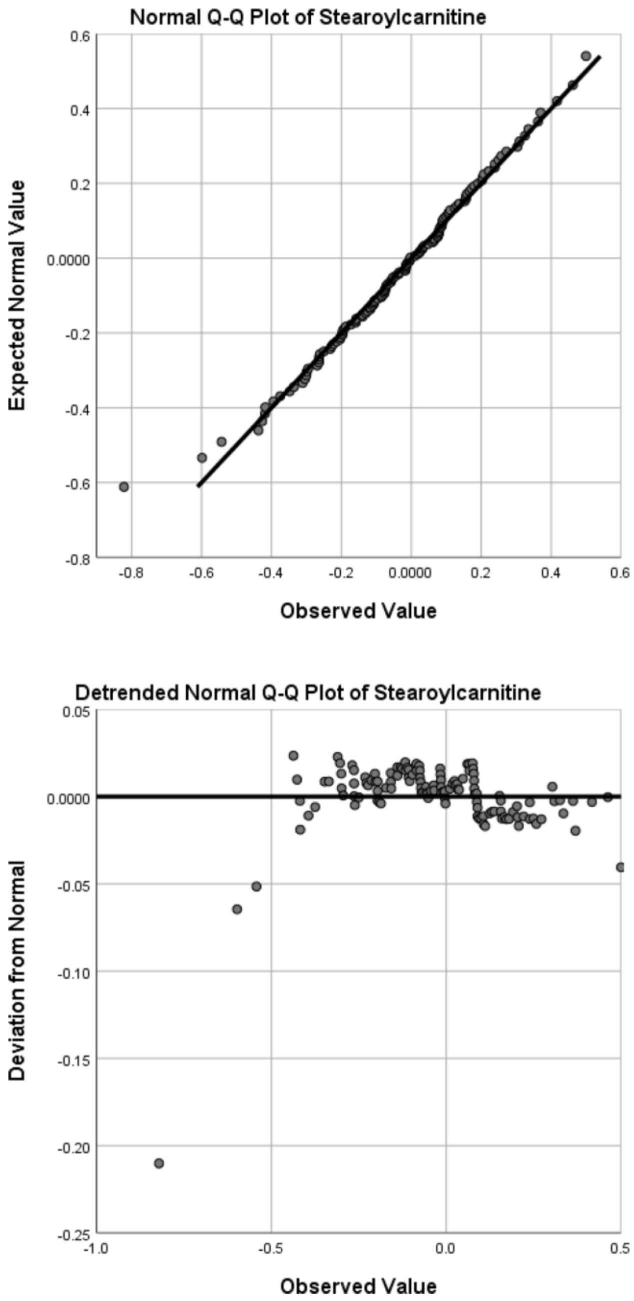

Supplementary Figure 75. The quantile-quantile (Q-Q) plot of normalized Stearoylcarnitine which demonstrated the consistency of normalized value with expected normal value (upper panel) and the deviation of normalized values from expected normal value (lower panel).

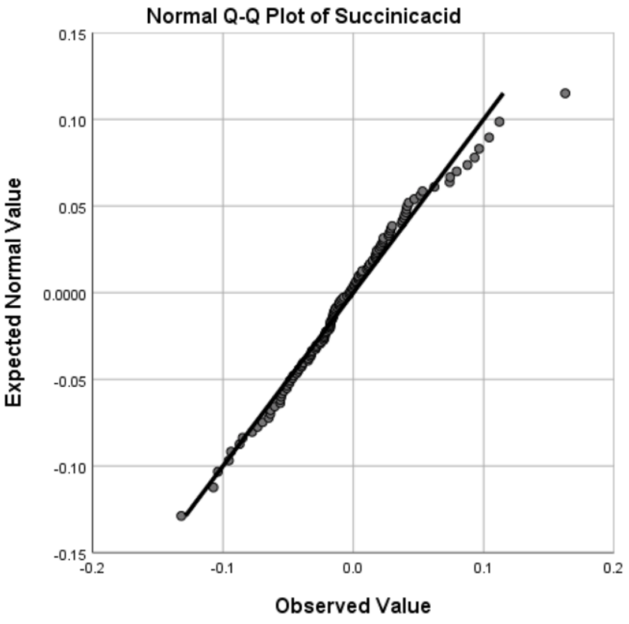

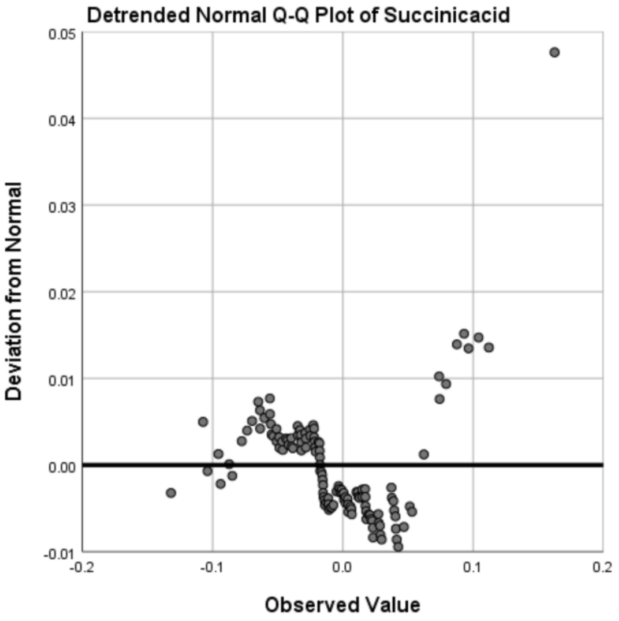

Supplementary Figure 76. The quantile-quantile (Q-Q) plot of normalized Succinicacid which demonstrated the consistency of normalized value with expected normal value (upper panel) and the deviation of normalized values from expected normal value (lower panel).

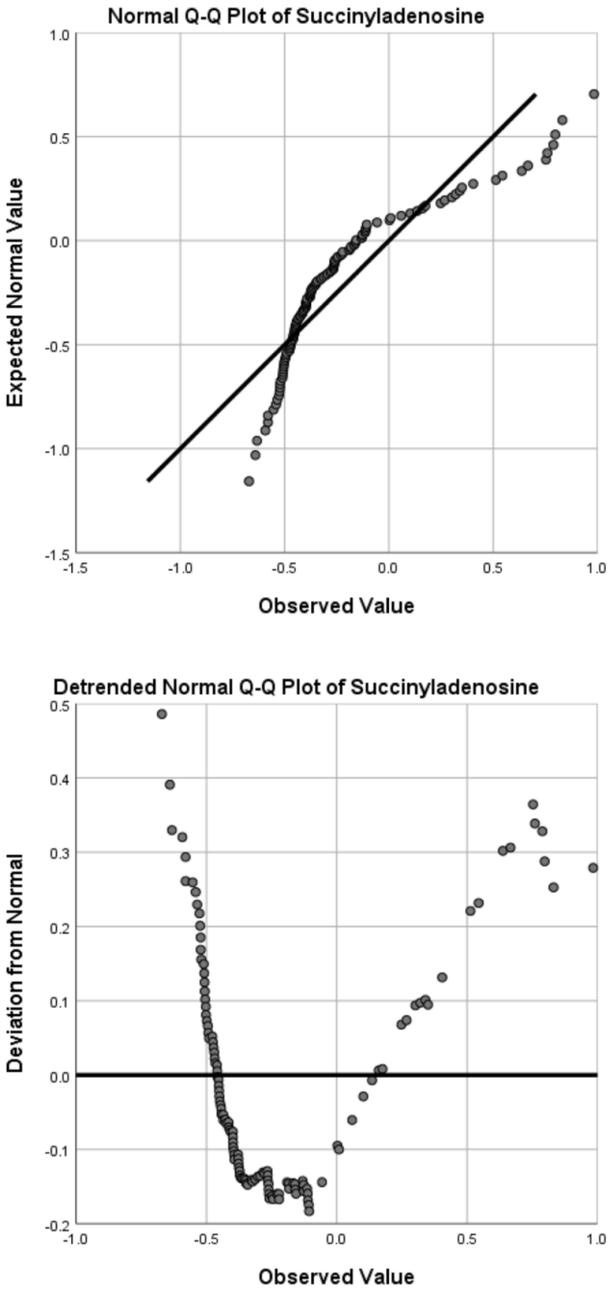

Supplementary Figure 77. The quantile-quantile (Q-Q) plot of normalized Succinyladenosine which demonstrated the consistency of normalized value with expected normal value (upper panel) and the deviation of normalized values from expected normal value (lower panel).

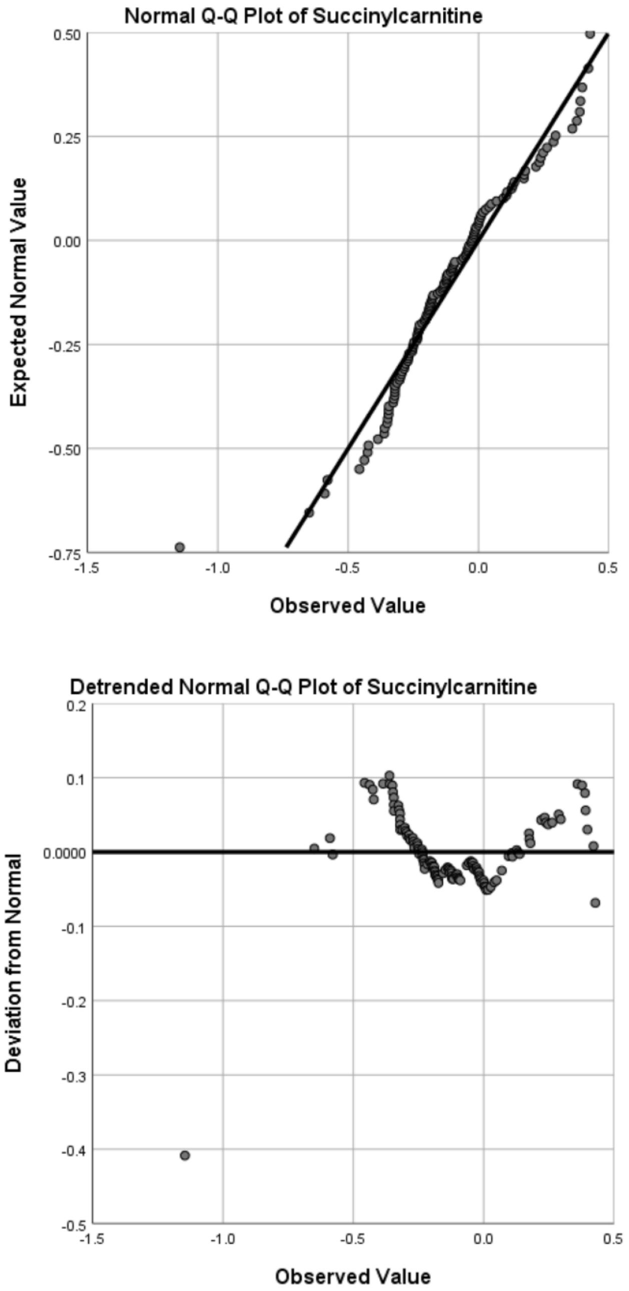

Supplementary Figure 78. The quantile-quantile (Q-Q) plot of normalized Succinylcarnitine which demonstrated the consistency of normalized value with expected normal value (upper panel) and the deviation of normalized values from expected normal value (lower panel).

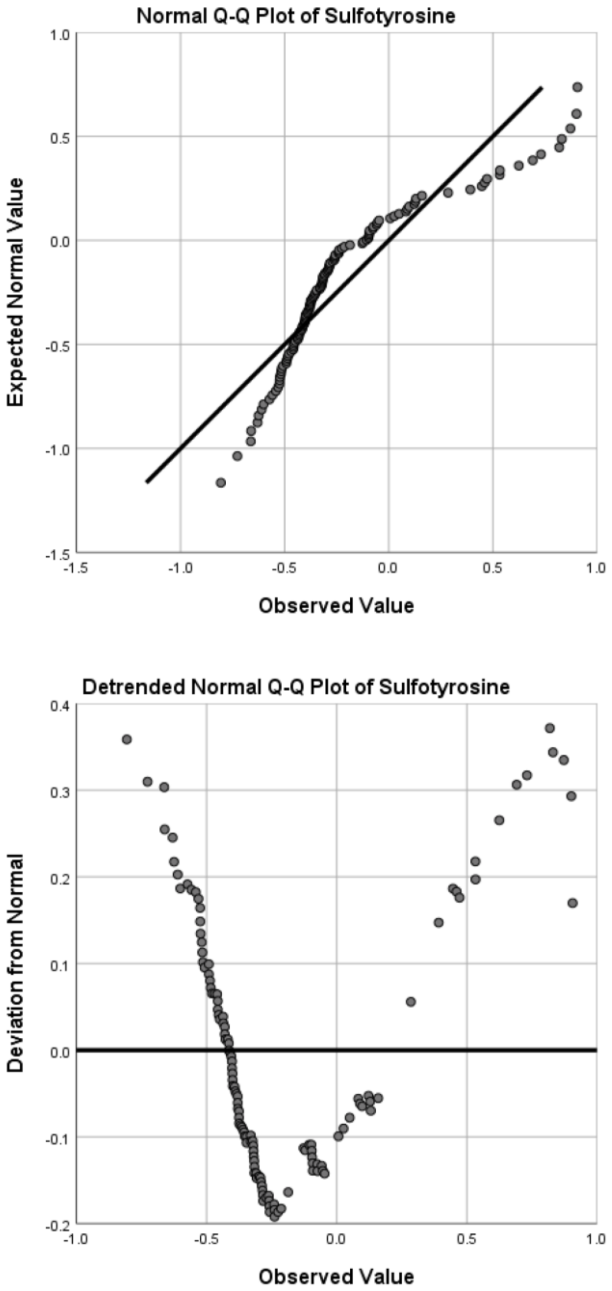

Supplementary Figure 79. The quantile-quantile (Q-Q) plot of normalized Sulfotyrosine which demonstrated the consistency of normalized value with expected normal value (upper panel) and the deviation of normalized values from expected normal value (lower panel).

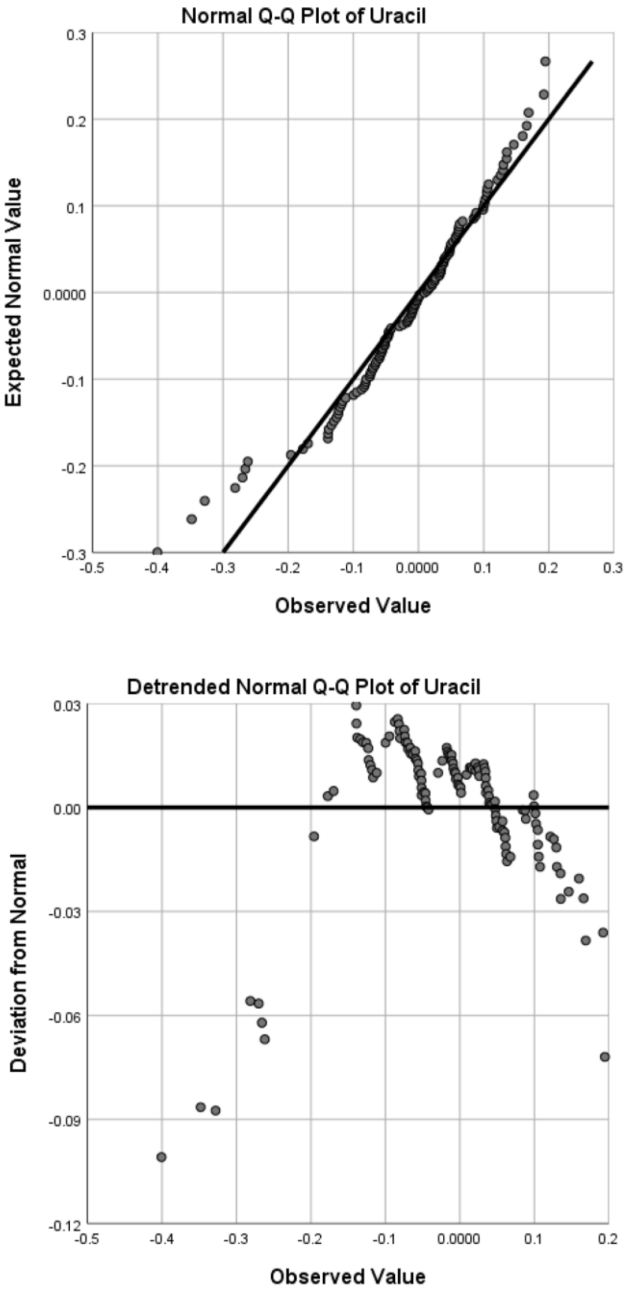

Supplementary Figure 80. The quantile-quantile (Q-Q) plot of normalized Uracil which demonstrated the consistency of normalized value with expected normal value (upper panel) and the deviation of normalized values from expected normal value (lower panel).

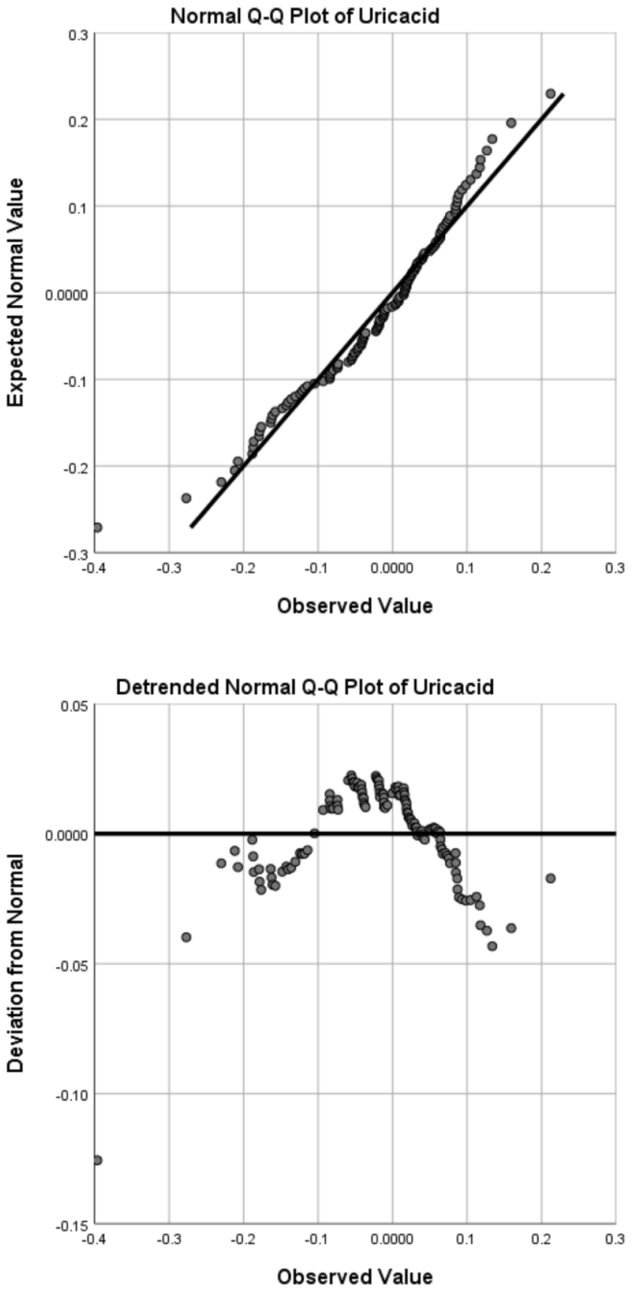

Supplementary Figure 81. The quantile-quantile (Q-Q) plot of normalized Uricacid which demonstrated the consistency of normalized value with expected normal value (upper panel) and the deviation of normalized values from expected normal value (lower panel).

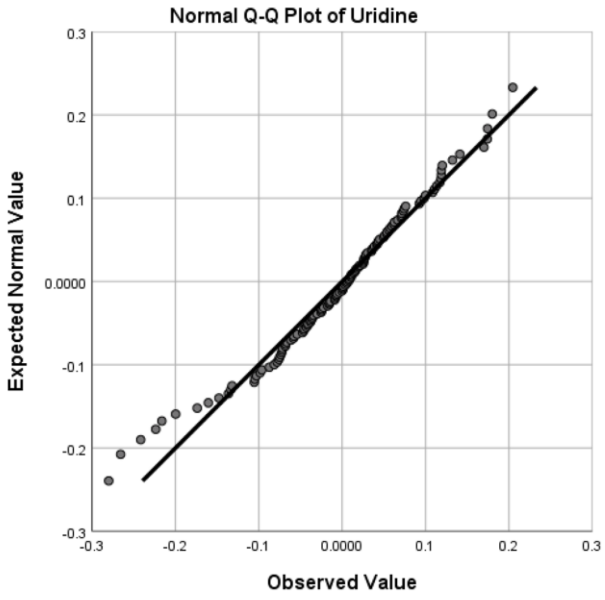

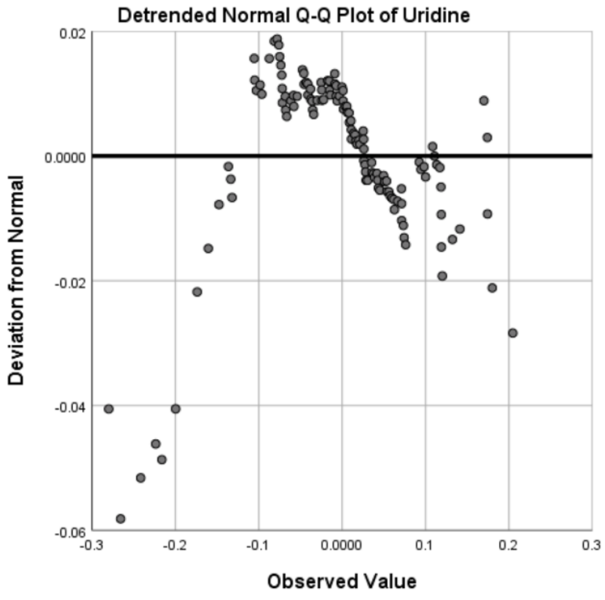

Supplementary Figure 82. The quantile-quantile (Q-Q) plot of normalized Uridine which demonstrated the consistency of normalized value with expected normal value (upper panel) and the deviation of normalized values from expected normal value (lower panel).

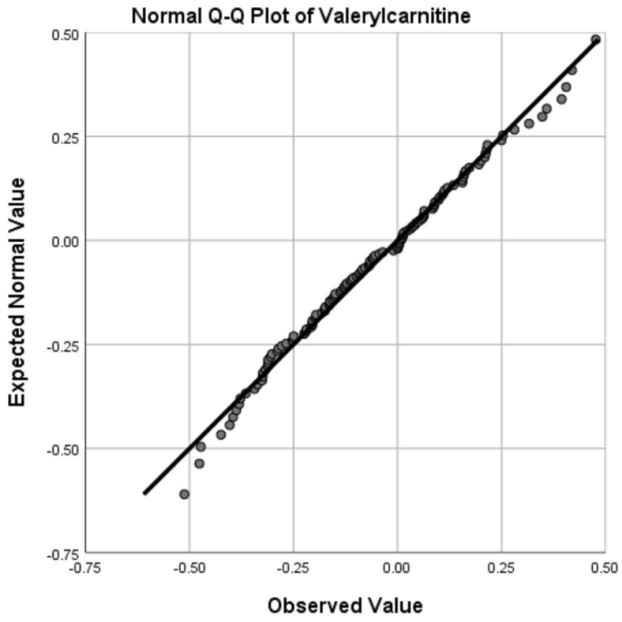

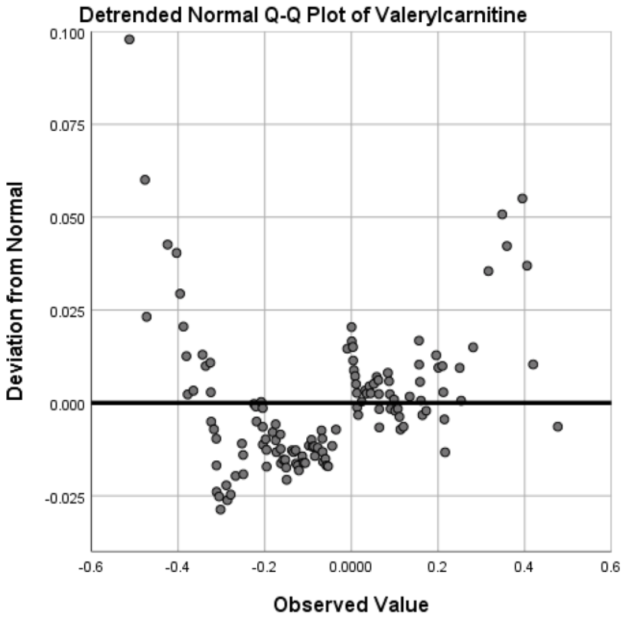

Supplementary Figure 83. The quantile-quantile (Q-Q) plot of normalized Valerylcarnitine which demonstrated the consistency of normalized value with expected normal value (upper panel) and the deviation of normalized values from expected normal value (lower panel).

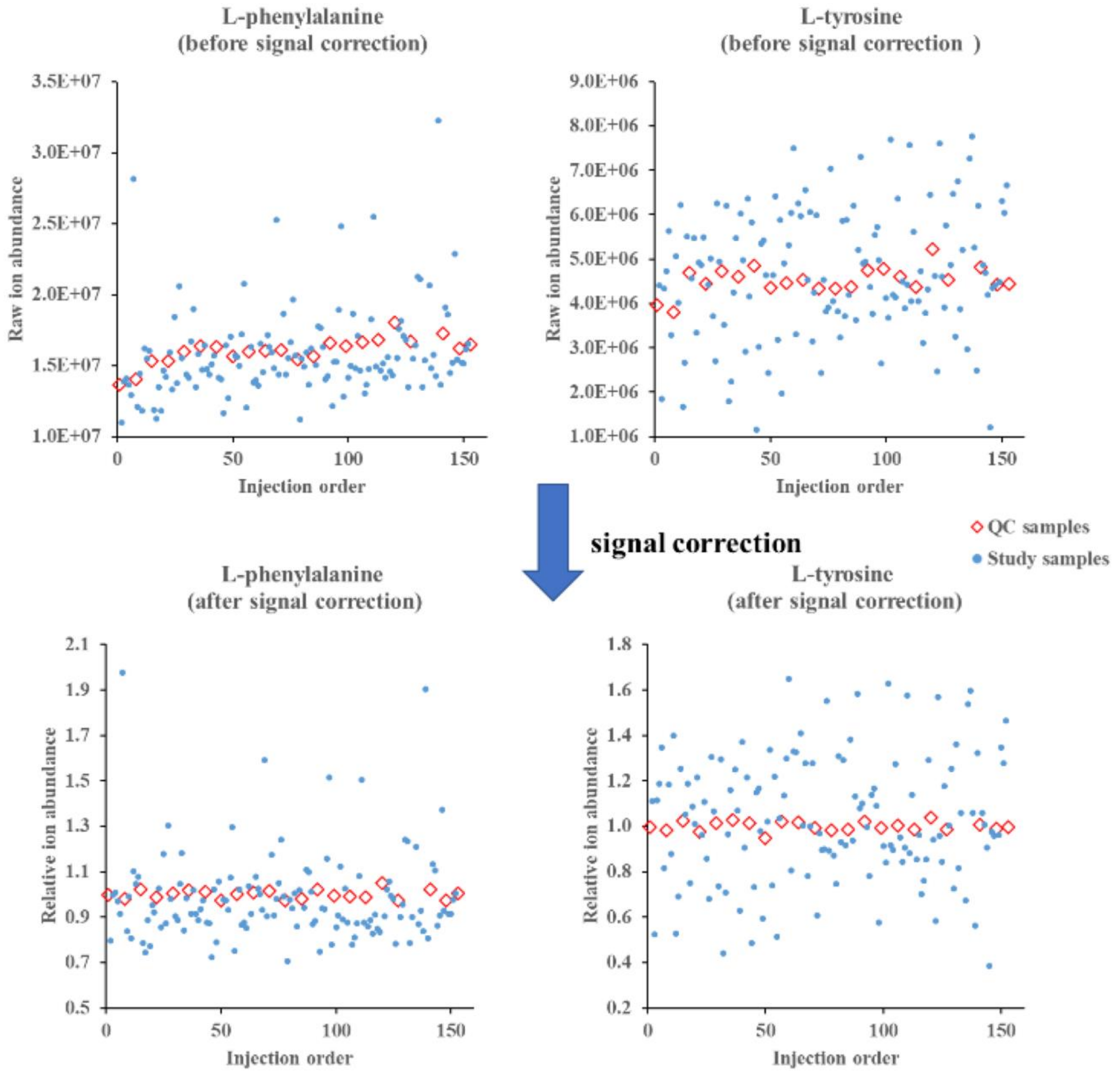

Supplementary Figure 84. Comparison of raw and relative ion abundances of L-tyrosine and L-phenylalanine in the study samples and QC samples before and after signal correction of data acquired by UPLC-Orbitrap-MS.
