# Supplementary material for: Insights into serum metabolic biomarkers for early detection of incident diabetic kidney disease in Chinese patients with type 2 diabetes by random forest
Source: Aging (Albany NY). 2024 Feb 12;16(4):3420–530. doi: 10.18632/aging.205542 (PMC10929832; doi:10.18632/aging.205542)
Supplement: Supplementary Tables 1, 3, 4, 7-9, 11, 12 and 14-16 [file aging-16-205542-s003.pdf]

SUPPLEMENTARY TABLES

Supplementary Table 1. Prevalence of microvascular and macrovascular complications in the patients with type 2 diabetes (*n* = 164) in each cohort.

	Diabetic retinopathy	Diabetic peripheral neuropathy	Macrovascular complication	At least one complication
**Discovery Cohort (%)**	21	38	43	58
**Validation Cohort (%)**	54	63	54	84
**Overall (%)**	32	46	46	67

Supplementary Table 3. The stability of relative ion abundance of internal standards in serum of subject samples and QC samples and overall features in QC samples.

Internal standards in serum of subject samples and QC samples							
**Internal standards**	**Dataset**	**Adduct**	**Retention time (min)**	**Detected m/z**	**Theoretical m/z**	**CV (%) of subject samples**	**CV (%) of quality control samples**
L-Tryptophan-(indole-D_5)	Discovery set	(M-H)−	2.95	208.1137	208.1140	18.04	2.16
	Validation set		2.96	208.1142	208.1140	27.08	1.61
	Discovery set	(M+H)+	2.95	210.1285	210.1285	4.24	2.43
	Validation set		2.96	210.1279	210.1285	12.14	4.28
Cholic acid D_4	Discovery set	(M-H)−	5.73	411.3054	411.3054	3.31	2.74
		(M+H-3H_2O)+	5.74	359.2878	359.2882	4.10	1.99
	Validation set	(M-H)−	5.72	411.3059	411.3054	3.44	1.31
		(M+H-3H_2O)+	5.73	359.2870	359.2882	9.82	2.84
cis-10-nonadecenoic acid	Discovery set	(M-H)−	13.68	295.2642	295.2643	29.61	15.28
	Validation set	(M-H)−	13.85	295.2647	295.2643	25.67	11.37
**Overall features in QC samples**							
**Dataset**	**ESI mode**	**QC samples (*n*)**	**Total features**	**Number of features with RSD ≤ 30% in QC samples**	**Number of features with RSD ≤ 20% in QC samples**	**Percentage in all features (RSD ≤ 30%)**	**Percentage in all features (RSD ≤ 20%)**
Discovery set	−	22	5234	4316	3596	82.46	68.70
	+	25	4701	3164	2368	67.30	50.37
Validation set	−	12	8302	7249	6255	87.32	75.34
	+	12	12802	10279	8125	80.29	63.47

Supplementary Table 4. Identification details and stability of metabolites in QC samples using UPLC-Orbitrap-MS.

Identified metabolites	Retention time (min)	Detected m/z	Theoretical m/z	Mass error (ppm)	Adduct	Molecular formula	Confirmation/ Supplier*	CV of QC (%)	
								Discovery set	Validation set
**L-Arginine**	0.67	173.1042	173.1044	0.6	$(M-H)^-$	C6H14N4O2	Sigma Aldrich	10.17	8.58
**L-Ornithine**	0.67	131.0823	131.0826	0	$(M-H)^-$	C5H12N2O2	Sigma Aldrich	11.08	5.23
**Choline**	0.78	104.1071	104.1070	−2.9	$(M)^+$	C5H14NO	Acros Organics	4.67	5.48
**L-Glutamine**	0.79	145.0616	145.0619	0.0	$(M-H)^-$	C5H10N2O3	Sigma Aldrich	1.08	1.25
**L-Citrulline**	0.80	174.0882	174.0884	0.6	$(M-H)^-$	C6H13N3O3	Sigma Aldrich	2.26	3.29
**D-Glucose**	0.80	215.0328	215.0328	1.4	$(M+Cl)^-$	C6H12O6	International laboratory	1.14	0.68
**L-Carnitine**	0.80	162.1125	162.1125	−3.1	$(M+H)^+$	C7H15NO3	CIL	1.50	3.63
**L−Glutamic acid**	0.80	148.0604	148.0604	−2.7	$(M+H)^+$	C5H9NO4	Sigma Aldrich	3.80	4.09

**L−Threonine**	0.80	120.0655	120.0655	−2.5	(M+H)$^+$	C4H9NO3	Sigma Aldrich	6.47	5.57
**Arabinose isomer**	0.80	195.0513	195.0510	1.5	(M+FA−H)−	C5H10O5	Santa Cruz	1.46	1.88
**Betaine**	0.81	118.0863	118.0863	−3.4	(M+H)$^+$	C5H11NO2	Sigma Aldrich	1.70	3.06
**Creatinine**	0.81	114.0662	114.0662	−2.6	(M+H)$^+$	C4H7N3O	Acros Organics	2.24	3.93
**2−Hydroxyethanesulfonate**	0.82	124.9911	124.9914	0.0	(M−H)$^-$	C2H6O4S	Sigma Aldrich	3.78	8.46
***γ*−Butyrobetaine**	0.83	146.1175	146.1176	−3.4	(M+H)$^+$	C7H15NO2	TRC	3.76	2.37
**L−Proline**	0.83	116.0707	116.0706	−2.6	(M+H)$^+$	C5H9NO2	Sigma Aldrich	1.56	3.69
**1,5−Anhydro−D−glucitol**	0.85	199.0376	199.0379	1.0	(M+Cl)−	C6H12O5	TRC	2.09	1.82
**N−Acetylcarnosine**	0.89	269.1243	269.1243	−2.6	(M+H)$^+$	C11H16N4O4	Santa Cruz	5.91	7.34
**5−Methylthio−D−ribose**	0.93	181.0529	181.0529	−2.8	(M+H)$^+$	C6H12O4S	Online database (HMDB)	6.12	8.15
**Pseudouridine**	0.93	243.0622	243.0623	1.2	(M−H)$^-$	C9H12N2O6	Supelco	1.78	3.65
**L−Valine**	0.94	118.0863	118.0863	−3.4	(M+H)$^+$	C5H11NO2	Sigma Aldrich	1.89	4.29
**L−Acetylcarnitine**	0.95	204.1230	204.1230	−2.9	(M+H)$^+$	C9H17NO4	CIL	2.35	6.67
**L,L−TMAP isomer**	0.96	229.1546	229.1547	−3.5	(M+H)$^+$	C11H20N2O3	ChemPartner [3]	4.05	6.07
**Uric acid**	1.00	169.0356	169.0356	−3.0	(M+H)$^+$	C5H4N4O3	Sigma Aldrich	1.78	4.69
**L,L−TMAP**	1.06	229.1546	229.1547	−3.5	(M+H)$^+$	C11H20N2O3	ChemPartner [3]	2.31	5.44
**L−Methionine**	1.07	148.0435	148.0438	1.8	(M−H)−	C5H11NO2S	Sigma Aldrich	5.03	7.31
**Citric acid**	1.09	191.0195	191.0197	1.0	(M−H)$^-$	C6H8O7	Sigma Aldrich	3.55	3.64
**Hydroxybutyrylcarnitine**	1.09	248.1491	248.1492	−3.6	(M+H)$^+$	C11H21NO5	Online databases	11.84	8.58
**Succinylcarnitine**	1.09	262.1284	262.1285	−3.8	(M+H)$^+$	C11H19NO6	Supelco	5.29	11.02
**Uracil**	1.09	113.0346	113.0346	−2.7	(M+H)$^+$	C4H4N2O2	Wako	5.11	6.35
**Uridine**	1.09	243.0622	243.0623	1.2	(M−H)$^-$	C9H12N2O6	Wako	1.69	1.14
**L−Tyrosine**	1.33	180.0664	180.0666	0.6	(M−H)$^-$	C9H11NO3	Sigma Aldrich	2.11	1.06
**Sulfotyrosine**	1.46	260.0234	260.0234	1.9	(M−H)$^-$	C9H11NO6S	Ref [4]	1.79	0.88
**Inosine**	1.50	267.0735	267.0735	1.9	(M−H)$^-$	C10H12N4O5	Acros Organics	2.30	1.17
**L−Leucine**	1.56	132.1019	132.1019	−3.0	(M+H)$^+$	C6H13NO2	Sigma Aldrich	1.81	6.27
**4−Acetamidobutanoic acid**	1.59	144.0664	144.0666	0.7	(M−H)$^-$	C6H11NO3	Matrix Scientific	1.62	1.44
**Propionylcarnitine**	1.67	218.1387	218.1387	−3.2	(M+H)$^+$	C10H19NO4	CIL	2.61	6.39
**2−Hydroxybutyric acid**	1.90	103.0398	103.0401	−1.0	(M−H)$^-$	C4H8O3	Sigma Aldrich	1.85	1.35
**2−(*α*−D−Mannopyranosyl) −L−tryptophan**	2.21	367.1497	367.1500	−3.8	(M+H)$^+$	C17H22N2O7	TRC	2.16	6.02
**L−Kynurenine**	2.42	209.0921	209.0921	−3.3	(M+H)$^+$	C10H12N2O3	Sigma Aldrich	3.00	8.09
**L−Phenylalanine**	2.48	164.0714	164.0717	0.6	(M−H)$^-$	C9H11NO2	Sigma Aldrich	2.02	2.36
**Succinyladenosine**	2.89	382.1005	382.1004	1.6	(M−H)$^-$	C14H17N5O8	TRC	3.06	3.07
***O*−Adipoylcarnitine**	2.90	290.1597	290.1598	−3.8	(M+H)$^+$	C13H23NO6	Supelco	3.39	5.11
**Butyrylcarnitine**	2.92	232.1543	232.1543	−3.4	(M+H)$^+$	C11H21NO4	CIL	2.62	5.65
**L−*β*−aspartyl−L−leucine**	2.93	247.1287	247.1288	−3.2	(M+H)$^+$	C10H18N2O5	Online database (HMDB)	6.71	4.89
**L−Tryptophan**	2.95	203.0824	203.0826	1.0	(M−H)$^-$	C11H12N2O2	Sigma Aldrich	2.21	1.26
**Homovanillic acid sulfate**	2.97	261.0073	261.0074	1.9	(M−H)$^-$	C9H10O7S	Cayman Chemical	12.40	2.42
**Kynurenic acid**	2.99	190.0499	190.0499	−3.2	(M+H)$^+$	C10H7NO3	Sigma Aldrich	14.71	4.95
**2−(3−(sulfooxy)phenyl)acet ic acid**	3.00	230.9967	230.9969	1.3	(M−H)$^-$	C8H8O6S	Online database (HMDB)	2.64	4.81
**Valerylcarnitine**	3.00	246.1699	246.1700	−3.7	(M+H)$^+$	C12H23NO4	Cayman Chemical	5.96	7.49
**Pyrocatechol sulfate**	3.07	188.9865	188.9863	1.1	(M−H)−	C6H6O5S	Online database (HMDB)	2.82	1.57
***α*−*N*−Phenylacetyl−L−glut amine**	3.07	263.1037	263.1037	1.9	(M−H)$^-$	C13H16N2O4	Santa Cruz	1.87	1.41
**Phenol sulfate**	3.10	172.9912	172.9914	1.2	(M−H)$^-$	C6H6O4S	Online databases	2.32	0.99
**Hexanoylcarnitine**	3.11	260.1855	260.1856	−3.5	(M+H)$^+$	C13H25NO4	Santa Cruz	28.21	8.88

**Hippuric acid**	3.14	178.0508	178.0510	0.6	$(M-H)^-$	C9H9NO3	Acros Organics	2.03	0.91
**Indoxyl sulfate**	3.15	212.0022	212.0023	1.4	$(M-H)^-$	C8H7NO4S	Sigma Aldrich	2.82	1.43
***p*−Cresol glucuronide**	3.17	283.0823	283.0823	1.8	$(M-H)^-$	C13H16O7	TRC	2.09	1.42
**2−Octenoylcarnitine**	3.32	286.2011	286.2013	−3.8	$(M+H)^+$	C15H27NO4	Online databases	3.15	6.83
***p*−Cresol sulfate**	3.36	187.0070	187.0071	1.1	$(M-H)^-$	C7H8O4S	CIL	1.82	1.00
**Indole−3−lactic acid**	3.44	204.0664	204.0666	1.0	$(M-H)^-$	C11H11NO3	Santa Cruz	2.25	1.28
**3−Hydroxydecanoyl carnitine**	3.62	332.2429	332.2431	−3.6	$(M+H)^+$	C17H33NO5	Online databases	5.28	5.99
**L−Octanoylcarnitine**	3.65	288.2167	288.2169	−3.5	$(M+H)^+$	C15H29NO4	CIL	2.63	5.72
**3−Indoleacetic acid**	3.78	176.0706	176.0706	−2.8	$(M+H)^+$	C10H9NO2	Sigma Aldrich	3.54	5.14
**Cortisol**	4.03	363.2163	363.2166	−3.6	$(M+H)^+$	C21H30O5	Sigma Aldrich	3.00	3.57
**9−Decenoylcarnitine**	4.13	314.2324	314.2326	−3.8	$(M+H)^+$	C17H31NO4	Online databases	2.18	6.51
**Bilirubin**	4.30	585.2706	585.2708	−2.7	$(M+H)^+$	C33H36N4O6	Acros Organics	4.95	5.13
**Decanoylcarnitine**	4.51	316.2480	316.2482	−3.8	$(M+H)^+$	C17H33NO4	Sigma Aldrich	2.80	2.82
**Dehydroepiandrosterone sulfate**	4.73	367.1584	367.1585	1.1	$(M-H)^-$	C19H28O5S	Cayman Chemical	3.63	1.64
**3,5−Tetradecadiencarnitine**	5.49	368.2793	368.2795	−3.5	$(M+H)^+$	C21H37NO4	Online databases	5.88	6.73
**Dodecanoylcarnitine**	5.57	344.2793	344.2795	−3.5	$(M+H)^+$	C19H37NO4	CIL	9.44	4.34
***cis*−5−Tetradecenoylcarnitine**	6.39	370.2949	370.2952	−3.5	$(M+H)^+$	C21H39NO4	Online databases	12.23	9.59
**LysoPE(18:1(11Z)/0:0)**	10.46	480.3083	480.3085	−2.9	$(M+H)^+$	C23H46NO7P	Online databases	14.54	9.97
**Stearoylcarnitine**	10.52	428.3732	428.3734	−3.3	$(M+H)^+$	C25H49NO4	CIL	16.91	8.10

*Acros Organics, NJ, USA. Cayman Chemical, Ann Arbor, MI, USA. Abbreviation: CIL: Cambridge Isotope Laboratories, Tewksbury, MA, USA. ChemPartner, Shanghai ChemPartner Co., Ltd., China. International Laboratory, San Francisco, CA, USA. Sigma Aldrich and Supelco, St. Louis, MO, USA. Matrix Scientific, Elgin, SC, USA. Santa Cruz, Dallas, TX, USA. TRC, Toronto Research Chemicals, Canada. Wako, Wako Pure Chemical Industries, Osaka, Japan. N, N, N−trimethyl−L−alanyl−Lproline betaine (L,L−TMAP).

Supplementary Table 7. Spearman rank correlation of metabolites with total BSA-related renal volume and renal resistive index in validation set (absolute Spearman *R* > 0.40).

Metabolites	Total BSA-related renal volume		Renal resistive index
	Validation (Stage 0–4) (*n* = 58)	Validation (Stage 1–4) (*n* = 48)	Validation (Stage 1–4) (*n* = 48)
**MDRD eGFR**	0.542	0.638	−0.525
**Bilirubin**	0.539	0.553	−0.423
**Stearoylcarnitine**	0.430	0.469	−0.148
**Ratio of tyrosine to phenylalanine**	0.387	0.425	−0.468
**L−Tryptophan**	0.319	0.375	−0.569
**L−Tyrosine**	0.309	0.326	−0.434
**SBP(mmHg)**	−0.082	−0.129	0.596
**N−Acetylcarnosine**	−0.137	−0.228	0.434
**Succinylcarnitine**	−0.206	−0.359	0.401
**Ratio of kynurenic acid to kynurenine**	−0.234	−0.309	0.499
**Citric acid**	−0.245	−0.322	0.484
**Phenol sulfate**	−0.248	−0.318	0.451
**Arabinose isomer**	−0.264	−0.402	0.607
**Indolelactic acid**	−0.307	−0.409	0.448
**3−hydroxydecanoyl carnitine**	−0.310	−0.489	0.384
**Indoleacetic acid**	−0.320	−0.444	0.196
**2−Octenoylcarnitine**	−0.342	−0.486	0.239

**MS−detected creatinine**	−0.352	−0.473	0.459
**Ratio of pseudouridine to uridine**	−0.369	−0.528	0.544
**L,L−TMAP isomer**	−0.376	−0.560	0.495
**O−Adipoylcarnitine**	−0.390	−0.578	0.356
**Urea (mmol/L)**	−0.391	−0.535	0.554
**Renal resistive index**	−0.392	−0.392	−
**2−(3−(sulfooxy)phenyl)acetic acid**	−0.401	−0.516	0.407
**2−Hydroxyethanesulfonate**	−0.429	−0.488	0.503
**Kynurenic acid**	−0.432	−0.537	0.567
**Homovanillic acid sulfate**	−0.436	−0.547	0.455
**4−Acetamidobutanoic acid**	−0.440	−0.557	0.494
**Sulfotyrosine**	−0.441	−0.572	0.535
**Serum Creatinine (mg/dL)**	−0.443	−0.571	0.446
**L,L−TMAP**	−0.449	−0.596	0.517
**Butyrylcarnitine**	−0.457	−0.617	0.369
**Pseudouridine**	−0.466	−0.599	0.588
**5−Methylthio−D−ribose**	−0.475	−0.618	0.466
**2−(α−D−Mannopyranosyl)−L−tryptophan**	−0.480	−0.604	0.586
**L−β−aspartyl−L−leucine**	−0.487	−0.589	0.578
**Serum cystatin C (mg/L)**	−0.503	−0.614	0.544
**Succinyladenosine**	−0.543	−0.615	0.556
**L−Kynurenine**	−0.562	−0.683	0.472
**Ratio of kynurenine to tryptophan**	−0.563	−0.640	0.613

Supplementary Table 8. Average AUC of single biomarkers for differentiation between stages in diabetic patients using RF classification in both discovery and validation sets.

Classification	Dataset	RF	MS-detected serum creatinine	Pseudouridine	L,L-TMAP	2-(α-D-Mannopyranosyl)-L-tryptophan	Succinyladenosine
**Stage 1a vs. Stage 1b-4**	Dis	AUC	0.849 ± 0.051	0.915 ± 0.054	0.898 ± 0.046	0.888 ± 0.066	0.888 ± 0.058
		Sens	0.721 ± 0.118	0.933 ± 0.053	0.810 ± 0.066	0.832 ± 0.120	0.832 ± 0.059
		Spec	0.876 ± 0.120	0.688 ± 0.148	0.902 ± 0.132	0.753 ± 0.156	0.791 ± 0.158
	Val	AUC	0.879 ± 0.078	0.915 ± 0.054	0.810 ± 0.066	0.893 ± 0.065	0.923 ± 0.050
		Sens	0.920 ± 0.100	0.933 ± 0.053	0.902 ± 0.132	0.852 ± 0.074	0.866 ± 0.067
		Spec	0.674 ± 0.183	0.688 ± 0.148	0.939 ± 0.022	0.806 ± 0.252	0.824 ± 0.179
**Stage 1a vs. Stage 1b-2**	Dis	AUC	0.754 ± 0.080	0.867 ± 0.072	0.848 ± 0.057	0.811 ± 0.074	0.816± 0.065
		Sens	0.643 ± 0.102	0.881 ± 0.089	0.751 ± 0.078	0.840 ± 0.093	0.688 ± 0.122
		Spec	0.805 ± 0.162	0.696 ± 0.138	0.812 ± 0.176	0.704 ± 0.145	0.807 ± 0.167
	Val	AUC	0.763 ± 0.109	0.757 ± 0.078	0.774 ± 0.115	0.780 ± 0.099	0.857 ± 0.066
		Sens	0.909 ± 0.115	0.651 ± 0.142	0.864 ± 0.121	0.672 ± 0.141	0.819 ± 0.143
		Spec	0.636 ± 0.209	0.760 ± 0.243	0.494 ± 0.209	0.794 ± 0.234	0.730 ± 0.173
**Stage 1b vs. Stage 2**	Dis	AUC	0.681 ± 0.094	0.741 ± 0.079	0.680 ± 0.078	0.707 ± 0.097	0.777 ± 0.069
		Sens	0.785 ± 0.177	0.679 ± 0.108	0.518 ± 0.127	0.687 ± 0.180	0.556 ± 0.121
		Spec	0.447 ± 0.187	0.772 ± 0.153	0.760 ± 0.185	0.598 ± 0.190	0.816 ± 0.170
	Val	AUC	0.600 ± 0.170	0.982 ± 0.031	0.668 ± 0.156	0.870 ± 0.092	0.889 ± 0.078
		Sens	0.480 ± 0.238	0.900 ± 0.100	0.478 ± 0.245	0.842 ± 0.258	0.766 ± 0.221
		Spec	0.684 ± 0.266	0.916 ± 0.150	0.778 ± 0.257	0.792 ± 0.152	0.802 ± 0.189
**Stage 1 vs. Stage 2-4**	Dis	AUC	0.918 ± 0.028	0.935 ± 0.035	0.906 ± 0.038	0.935 ± 0.026	0.899 ± 0.038

		Sens	0.724 ± 0.071	0.874 ± 0.058	0.782 ± 0.057	0.778 ± 0.064	0.787 ± 0.059
		Spec	0.907 ± 0.095	0.905 ± 0.083	0.939 ± 0.081	0.920 ± 0.098	0.902 ± 0.092
		AUC	0.927 ± 0.048	0.994 ± 0.010	0.928 ± 0.046	0.954 ± 0.034	0.971 ± 0.031
	Val	Sens	0.811 ± 0.081	0.966 ± 0.036	0.784 ± 0.085	0.939 ± 0.094	0.931 ± 0.079
		Spec	0.910 ± 0.145	0.976 ± 0.063	0.929 ± 0.141	0.857 ± 0.083	0.925 ± 0.106
		AUC	0.950 ± 0.025	0.982 ± 0.021	0.969 ± 0.020	0.974 ± 0.021	0.958 ± 0.028
	Dis	Sens	0.919 ± 0.079	0.904 ± 0.076	0.949 ± 0.057	0.923 ± 0.065	0.912 ± 0.075
**Stage 1-2 vs. Stage 3-4**		Spec	0.880 ± 0.080	0.949 ± 0.044	0.851 ± 0.074	0.898 ± 0.055	0.841 ± 0.077
		AUC	0.931 ± 0.039	0.951 ± 0.034	0.915 ± 0.046	0.939 ± 0.039	0.912 ± 0.044
	Val	Sens	0.932 ± 0.066	0.905 ± 0.096	0.845 ± 0.111	0.786 ± 0.133	0.899 ± 0.086
		Spec	0.817 ± 0.109	0.883 ± 0.090	0.858 ± 0.088	0.863 ± 0.136	0.843 ± 0.100
		AUC	0.989 ± 0.009	0.992 ± 0.020	0.982 ± 0.034	0.988 ± 0.024	0.993 ± 0.018
	Dis	Sens	0.967 ± 0.074	0.914 ± 0.096	0.934 ± 0.114	0.926 ± 0.098	0.942 ± 0.093
**Stage 1-3 vs. Stage 4**		Spec	0.975 ± 0.015	0.973 ± 0.027	0.950 ± 0.028	0.973 ± 0.028	0.983 ± 0.015
		AUC	0.976 ± 0.081	0.968 ± 0.061	0.984 ± 0.054	0.953 ± 0.055	0.955 ± 0.051
	Val	Sens	0.952 ± 0.163	0.882 ± 0.168	0.938 ± 0.135	0.906 ± 0.111	0.886 ± 0.124
		Spec	1.000 ± <0.001	0.983 ± 0.022	1.000 ± <0.001	0.995 ± 0.020	0.987 ± 0.039

Data were expressed as mean ± SD. Abbreviations: Dis: discovery; Val: validation; RF: random forest; Sens: sensitivity; Spec: specificity.

Supplementary Table 9. Multivariate linear regression analyses among biomarkers, sex and log (MDRD eGFR) trained with discovery set and tested with validation set among diabetic patients.

**log (MDRD GFR)**	**Discovery set**				**Validation set**
	β	***p*-value**	***R*^{2*}**	**RMSE**	***R*^{2†}**
**Model 1**			0.9583	0.0765	0.9709
**log (MS-detected creatinine)**	−1.25 (−1.30 to −1.20)	$p < 0.0001$			
**sex**	0.16 (0.13 to 0.19)	9.76E-18			
**Model 2**			0.9531	0.0812	0.9528
**log (pseudouridine)**	−1.22 (−1.27 to −1.17)	$p < 0.0001$			
**sex**	0.02 (−0.02 to 0.05)	0.3352			
**Model 3**			0.9375	0.0938	0.9569
**log (L,L-TMAP)**	−0.95 (−1.00 to −0.91)	$p < 0.0001$			
**sex**	0.06 (0.03 to 0.10)	0.0009			
**Model 4**			0.8908	0.1240	0.9116
**log (succinyladenosine)**	−0.94 (−1.01 to −0.88)	$p < 0.0001$			
**Sex**	0.01 (−0.03 to 0.06)	0.5662			
**Model 5**			0.8690	0.1358	0.9499
**log (2-(*α*-D-mannopyranosyl)-L-tryptophan)**	−0.67 (−0.72 to 0.62)	$p < 0.0001$			
**sex**	0.01 (−0.04 to 0.06)	0.6834			

β, unstandardized coefficient of linear regression. sex, female = 1 and male = 2. Discovery set, *n* = 106; validation set, *n* =56. RMSE, root mean square error. $^{*}R^2$ was based on the predicted log (MDRD eGFR) against actual log (MDRD eGFR) using the equation of the model and data of discovery set. $^{\dagger}R^2$ was based on the predicted log (MDRD eGFR) against actual log (MDRD eGFR) using the equation of the model of discovery set and data of validation set.

Supplementary Table 11. Analysis of 106 follow-up patients by random forest.

ABCD group		AB group		CD group	
**Index**	**Mean AUC and Quartile of 100 Iterations**	**Index**	**Mean AUC and Quartile of 100 Iterations**	**Index**	**Mean AUC and Quartile of 100 Iterations**
Sex	0.5625 (0.5234–0.625)	Sex	0.6429 (0.5–0.7143)	Sex	0.5556 (0.5–0.6667)
Age	0.7031 (0.6445–0.7822)	Age	0.7245 (0.6327–0.8367)	Age	0.7407 (0.6327–0.8302)
eGFR	0.7822 (0.7031–0.8359)	eGFR	0.8367 (0.6939–0.9184)	SAdo	0.7809 (0.6975–0.858)
Pseu	0.8125 (0.7397–0.8867)	SAdo	0.7296 (0.6607–0.8393)	eGFR	0.7963 (0.6914–0.8704)
SAdo	0.8174 (0.748–0.8716)	Pseu	0.8622 (0.7245–0.9388)	Cr	0.8086 (0.7207–0.8781)
Cr	0.8438 (0.7925–0.8926)	UACR	0.898 (0.8367–0.9796)	ADT	0.8117 (0.713–0.9012)
ADT	0.8691 (0.8179–0.918)	Cr	0.9133 (0.8265–0.9796)	Pseu	0.8302 (0.7346–0.909)
UACR	0.8887 (0.8262–0.9185)	ADT	0.9184 (0.8673–0.9923)	UACR	0.8889 (0.7901–0.9213)
ADT+UACR	0.9443 (0.9141–0.9727)	ADT+UACR	0.9796 (0.9388–1)	SAdo+UACR	0.9012 (0.8194–0.9506)
UACR+ADT +SAdo+Cr	0.9482 (0.9248–0.9805)	ADT+SAdo+UA CR	0.9796 (0.9388–1)	ADT+UACR	0.9136 (0.8395–0.963)
ADT+SAdo+ UACR	0.9502 (0.9062–0.9805)	UACR+ADT+Cr	1 (0.9592–1)	UACR+Pseu+ Cr	0.929 (0.8765–0.9753)
		UACR+ADT+Ag e+Sex	1 (0.9592–1)	UACR+Pseu	0.9352 (0.8765–0.9753)

Pseu:pseudouridine.

Supplementary Table 12. Pearson correlation of MDRD GFR with three another GFRs that were calculated by the three newly reported equations.

**GFR calculation methods**	**Equations**	**Pearson correlation coefficients with MDRD GFR**
**MDRD Study equation**[1]	Estimated GFR = 186 × (serum creatinine)$^{-1.154}$ × (age in years)$^{-0.203}$ × 0.742 (if female) × 1.210 (if African American)	–
**CKD-EPI** $_{\text{creatinine}}$ **equation**[2]	Estimated GFR = 141 × min (serum creatinine/κ, 1)α × max (serum creatinine/κ, 1)$^{-1.209}$ × 0.993$^{\text{Age in year}}$ × 1.018 (if female) × 1.159 (if black), where κ is 0.7 for females and 0.9 for males, α is −0.329 for females and −0.411 for males. min indicates the minimum of ratio of serum creatinine to κ or 1, and max indicates the maximum of ratio of serum creatinine to κ or 1.	Discovery (*n* = 128): 0.9523 Validation (*n* = 66): 0.9729
**CKD-EPI** $_{\text{cystatin C}}$ **equation**[2]	Estimated GFR = 133 × min(serum cystatin C/0.8, 1)$^{-0.499}$ × max(serum cystatin C/0.8, 1)$^{-1.328}$ × 0.996^{Age} × 0.932 (if female), where min indicates the minimum of ratio of serum cystatin C to 0.8 or 1, and max indicates the maximum of ratio of serum cystatin C to 0.8 or 1.	Validation (*n* = 58): 0.9468
**CKD-EPI** $_{\text{creatinine–cystatin C}}$ **equation**[2]	Estimated GFR = 135 × min (serum creatinine/κ, 1)$^{\alpha}$ × max (serum creatinine/κ, 1)$^{-0.601}$ × min (serum cystatin C/0.8, 1)$^{-0.375}$ × max(serum cystatin C/0.8, 1)$^{-0.711}$ × 0.995^{Age} × 0.969 (if female) × 1.08 (if black), where κ is 0.7 for females and 0.9 for males, and α is −0.207 for males and −0.248 for females.	Validation (*n* = 58): 0.9681

Supplementary Table 14. Gradient diluted and corresponding concentrations of standards.

	100% (ng/ml)	90% (ng/ml)	80% (ng/ml)	60% (ng/ml)	50% (ng/ml)	40% (ng/ml)	20% (ng/ml)	10% (ng/ml)	8% (ng/ml)	6% (ng/ml)
2-(A-D-Mannopyranosyl-) L-Tryptophan	50.00	45.00	40.00	30.00	25.00	20.00	10.00	5.00	4.00	3.00
Succinyladenosine	58.00	52.20	46.40	34.80	29.00	23.20	11.60	5.80	4.64	3.48
pseudouridine	160.00	144.00	128.00	96.00	80.00	64.00	32.00	16.00	12.80	9.60

Supplementary Table 15. Recovery rate and precision of selected metabolites.

Metabolite	Recovery rate
2-(A-D-Mannopyranosyl-)L-Tryptophan	83%
Succinyladenosine	95%
pseudouridine	94%
Tryptophan-D5	95%

Supplementary Table 16. Q1/Q3 mass and MRM conditions for the selected metabolites.

Metabolite	Q1 mass (Da)	Q3 mass (Da)	Dwelling time (msec)	Declustering Potential (volts)	Collision energy (volts)
2-(A-D-Mannopyranosyl-) L-Tryptophan	367.1	247.1	100	152.15	19.3
Succinyladenosine	384.1	252	100	122.58	27.09
pseudouridine	245.1	191	100	108.61	19.97
Tryptophan-D5	210	192	100	61.85	16.69
