# Supplementary material for: Insights into serum metabolic biomarkers for early detection of incident diabetic kidney disease in Chinese patients with type 2 diabetes by random forest
Source: Aging (Albany NY). 2024 Feb 12;16(4):3420–530. doi: 10.18632/aging.205542 (PMC10929832; doi:10.18632/aging.205542)
Supplement: Supplementary Tables 2, 5, 6, 10 and 13 [file aging-16-205542-s004.docx]

**Supplementary Table 2. Clinical features of participants in the discovery and validation cohorts.**

| Clinical parameters | Discovery cohort | | | | | | Validation cohort | | | | | | |
| --- | --- | --- | --- | --- | --- | --- | --- | --- | --- | --- | --- | --- | --- |
|  | **Stages (eGFR ranges, mL/min/1.73 m^2^)** | | | | | | **Stages (eGFR ranges, mL/min/1.73 m^2^)** | | | | | | |
|  | **0**  **(90-120)** | **1a***  **(≥119)** | **1b**  **(90-118)** | **2**  **(60-89)** | **3**  **(30-59)** | **4**  **(<30)** | **0**  **(90-120)** | **1a***  **(≥119)** | **1b**  **(90-118)** | **2**  **(60-89)** | **3**  **(30-59)** | **4**  **(<30)** |  |
| n | 20 | 20 | 19 | 30 | 29 | 20 | 10 | 10 | 10 | 10 | 15 | 11 |  |
| Age (years) | 50(7) | 51(5) | 54(5) ^a^ | 55(7) ^a^ | 60(9) ^abcd^ | 61(5)^abcd^ | 50(5.6) | 50(2) | 50(3) | 54(7) | 58.9(6) ^abc^ | 55.7(9) |  |
| Sex (Male, n) | 10 | 10 | 10 | 15 | 9 | 12 | 5 | 5 | 5 | 5 | 9 | 7 |  |
| Duration of diabetes (years) | - | 7(6) | 5(6) | 10(6) ^c^ | 10(8) ^c^ | 12(6)^bc^ | - | 5.9(4.7) | 8.7(3.3) | 9.3(7.4) | 8.1 (4.7) | 15.4(10.2)^be^ |  |
| BMI (kg/m^2^) | 21.8(1.5) | 23.3(4.1) | 25.7(3.5)^a^ | 25.2 (3.1) ^a^ | 27.0(3.0)^ab^ | 27.0(3.3) ^a^ | 20.8(1.2) | 23.4(2.8) ^a^ | 24.9(1.5) ^af^ | 25.9(3.5) ^af^ | 25.0 (3.0) ^af^ | 22.5(2.8) |  |
| Systolic BP (mmHg) | 117(13) | 128(20) ^a^ | 136(23) ^a^ | 132(20) ^a^ | 135(20) ^a^ | 149(26)^abd^ | 111(5) | 122(17) | 126(2.3) | 147 (19) ^abc^ | 139 (20) ^ab^ | 152(22)^abc^ |  |
| Diastolic BP (mmHg) | 74(11) | 80(11) | 86(14) ^abf^ | 78(12) | 79(9) | 77(12) | 77(7) | 82(11) | 87(21) | 91 (9) ^a^ | 86 (12) | 85(11) |  |
| Total cholesterols (mmol/L) | 4.7(0.5) | 5.3(1.1) ^ac^ | 4.5(1.2) | 4.9(1.2) | 5.0(1.4) | 4.8(1.8) | 4.5(0.5) | 5.0(0.7) | 5.2(1.1) | 4.7 (0.9) | 4.9 (1.1) | 4.8(1.5) |  |
| Serum creatinine (umol/L) | 65(62-75) | 49(45-62) ^a^ | 73(55-77) ^b^ | 83(72-91) ^abc^ | 121(107-139) ^abcd^ | 335(232-627)^abcde^ | 62(51-63) | 48(43-60) ^a^ | 65(56-73)^b^ | 86 (72-106) ^abc^ | 119 (101-125) ^abcd^ | 709(289-858) ^abcde^ |  |
| eGFR (ml/min/1.73 m^2^) | 97(93-104)^def^ | 135(124-151) ^acdef^ | 102(96-105) ^def^ | 78(72-84) ^ef^ | 52 (41-56)^f^ | 15(6-25) | 110(98-120) ^def^ | 137(121-149) ^acdef^ | 104(99-110)^def^ | 75 (70-78) ^ef^ | 55 (43-58)^f^ | 7(5-21) |  |
| FBG (mmol/L) | 5.1(0.3) | 9.9(2.7) ^acdef^ | 7.6(2.2) ^af^ | 8.0(2.7) ^af^ | 7.18(2.6)^af^ | 4.2(1.2) ^a^ | 4.2(0.5) | 10.8(4.3)^ab^ | 8.3(2.3) ^a^ | 6.7(3.8) | 7.8(3.1) ^a^ | 9.8(6.4)^a^ |  |
| HbA_1c_ (%) | - | 9.9(2.1)^cf^ | 7.8(1.5) ^b^ | 9.06 (1.7)^f^ | 8.3(2.2) ^b^ | 6.6(1.3) ^bcde^ | 5.4(0.3) | 9.79(3.2)^ade^ | 8.1(1.4) ^a^ | 7.0(1.5) ^a^ | 7.71.5) ^a^ | 8.2(2.6) ^a^ |  |
| Serum urea (mmol/L) | 4.3(1.1) | 4.3(0.9) | 4.9(0.9) ^b^ | 4.9(1.3) | 8.2(2.3)^abcd^ | 20.4(8.9)^abcde^ | 5.1(1.4) | 5.0(1.0) | 4.4(1.3) | 6.7 (1.8) ^abc^ | 7.7 (3.1) ^abc^ | 20.4(7.6)^abcde^ |  |
| Serum uric acid (μmol/L） | 312(57) | 305(81) | 340 (89) | 324 (75) | 416(73) ^abcd^ | 424(89)^abcd^ | 256(48) | 297(107) | 336(113) | 368(115) ^a^ | 377(121) ^a^ | 429(157) ^ab^ |  |
| UACR (mg/g Cr.) | 2.7(2.33-3.59) | 27(3.3-59) ^a^ | 12(2.8-34) ^a^ | 103(17-329) ^abc^ | 357(191-1325)^abcd^ | 2337(1556-3814)^abcde^ | 2.7(1.8-4.3) | 17(8.6-65) ^a^ | 4.5(2.0-39) | 42(7.0-159) | 73(10-426) | 2518(1784-4374) ^abcde^ |  |
| Urinary creatinine (μmol/L) | 11793(7909-16647) ^cf^ | 9369(6552-14750)^f^ | 6900(4795-14550) ^f^ | 8846(5144-9828) ^a^ | 6560(4595-9769) ^ab^ | 4285(3462-5807) ^abcde^ | 8014(3877-13799) | 12211(5264-13947) | 9478(8607-12632) | 10270 (8543-14183) | 8498(6812-13085) | 8287(5891-10347) |  |
| Urinary microalbumin(mg/L) | - | 30(6.3-51) | 12 (0.9-23) | 82 (4.7-216) ^bc^ | 305(120-944)^bc^ | 827(99-1680)^bcde^ | 3.9(0.5-5.0) | 20(11-37) | 5.0(3.3-64) | 41(9.9-296) ^a^ | 163(15-971) ^a^ | 2027(825-3687) ^abcde^ |  |
| Urinary *β*2-MG (mg/L) | 0.14(0.06-0.20) | 0.09(0.01-0.17)^a^ | 0.12(0.03-0.18) | 0.23(0.08-0.60) | 0.42(0.20-3.74) | 17.58(2.06-27.27) ^abcde^ | 0.09(0.04- 0.16) | 0.07(0.05-0.21) | 0.09 (0.06-0.13) | 0.13(0.11-0.34) | 1.32(0.42-5.07) | 32.79(15.21-72.81) ^abcde^ |  |
| Antihypertensive treatment (%) | - | 20 | 16 | 53 | 45 | 90 | - | 10 | 40 | 70 | 73 | 73 |  |
| Antidyslipidemia treatment (%) | - | 25 | 26 | 30 | 45 | 45 | - | 0 | 10 | 10 | 27 | 0 |  |
| Serum cystatin C (mg/L) | - | - | - | - | - | - | 0.8(0.7-0.8) | 0.7(0.6-0.8) | 0.8(0.7-0.9) | 1.2 (1.0-1.4)^abc^ | 1.8 (1.3-2.1) ^abcd^ | 6.47(4.3-8.0) ^abcde^ |  |

Data are expressed as mean (SD), median (IQR), or count (*n*). Significance level for superscript case letters (^a,b,c,d,e,f^ represent comparison with Stages 0, 1a, 1b, 2, 3 and 4, respectively): Unpaired Student’s *t*-tests, *p* ≤ 0.05. *Stage 1a is defined as two IQRs of median of normal participants in Stage 0 in the discovery cohort.

Supplementary Table 5. Metabolites that had significant fold changes at different stages with respect to the healthy subjects in both discovery and validation sets.

| **Metabolites** | **Fold change (median) *** | | ***P* value** | | ***FDR*** |  |
| --- | --- | --- | --- | --- | --- | --- |
|  | **Discovery set** | **Validation set** | **Discovery set** | **Validation set** | **Discovery set** | **Validation set** |
| **Stage 1a *vs* healthy subjects** |  |  |  |  |  |  |
| **Hydroxybutyrylcarnitine** | 4.66 | 2.33 | 0.0095 | 0.0067 | 0.037 | 0.053 |
| **D-Glucose** | 1.82 | 2.19 | 1.63E-08 | 0.0001 | 0.098 | 0.0824 |
| **Stearoylcarnitine** | 1.44 | 1.66 | 0.0064 | 0.0099 | 0.0577 | 0.0222 |
| **2-Hydroxybutyric acid** | 1.38 | 2.2 | 0.0014 | 3.72E-07 | 0.069 | 0.0396 |
| **Cortisol** | 1.35 | 1.79 | 0.0189 | 0.0003 | 0.0336 | 0.0377 |
| **L-Leucine** | 1.26 | 1.37 | 0.0039 | 0.0291 | 0.012 | 0.0741 |
| ***γ*-Butyrobetaine** | -1.28 | -1.2 | 0.0015 | 0.0403 | 0.0249 | 0.0553 |
| **L-Glutamine** | -1.56 | -1.35 | 1.06E-10 | 0.0025 | 0.0328 | 0.0991 |
| **1,5-Anhydro-D-glucitol** | -26.5 | -17.75 | 1.68E-11 | 0.0005 | 0.0061 | 0.0183 |
|  |  |  |  |  |  |  |
| **Stage 1b *vs* healthy subjects** |  |  |  |  | 0.0732 | 0.039 |
| **Hydroxybutyrylcarnitine** | 3.91 | 1.62 | 0.0015 | 0.0032 | 0.0316 | 0.0211 |
| **Ratio of glutamic acid to glutamine** | 2.28 | 1.43 | 7.80E-07 | 0.0294 | 0.0655 | 0.0761 |
| **L-Leucine** | 1.39 | 1.54 | 2.11E-05 | 0.0002 | 0.0534 | 0.0091 |
| **D-Glucose** | 1.32 | 1.65 | 2.73E-05 | 0.0001 | 0.0842 | 0.0615 |
| **2-Hydroxybutyric acid** | 1.17 | 1.87 | 0.0371 | 5.83E-06 | 0.0443 | 0.0298 |
| **L-Glutamine** | -1.25 | -1.19 | 0.0001 | 0.0031 | 0.0233 | 0.0192 |
| **Ratio of glutamine to glutamic acid** | -2.28 | -1.43 | 7.80E-07 | 0.0294 | 0.0761 | 0.0274 |
| **1,5-Anhydro-D-glucitol** | -7.88 | -7.18 | 4.93E-06 | 8.40E-06 | 0.0175 | 0.0302 |
|  |  |  |  |  |  |  |
| **Stage 2 *vs* healthy subjects** |  |  |  |  | 0.0397 | 0.0581 |
| **Hydroxybutyrylcarnitine** | 5.29 | 1.78 | 4.46E-05 | 0.0028 | 0.0307 | 0.0745 |
| **Arabinose isomer** | 2.4 | 1.7 | 8.83E-11 | 0.0006 | 0.0507 | 0.031 |
| **2-(*α*-D-Mannopyranosyl)-L-tryptophan** | 2.08 | 1.43 | 1.46E-07 | 0.0001 | 0.025 | 0.0165 |
| **2-[3-(sulfooxy)phenyl]acetic acid** | 1.86 | 2.39 | 0.0047 | 0.0011 | 0.0644 | 0.0973 |
| **4-Acetamidobutanoic acid** | 1.61 | 1.58 | 0.0001 | 0.001 | 0.0411 | 0.0783 |
| **Succinyladenosine** | 1.55 | 1.35 | 2.29E-07 | 0.0107 | 0.0719 | 0.0469 |
| **D-Glucose** | 1.41 | 1.24 | 9.69E-07 | 0.0325 | 0.0515 | 0.038 |
| **LysoPE(18:1(11Z)/0:0)** | 1.38 | 1.43 | 0.0004 | 0.0116 | 0.0439 | 0.0613 |
| **L,L-TMAP isomer** | 1.36 | 1.52 | 2.42E-05 | 0.0033 | 0.0246 | 0.0941 |
| **Succinylcarnitine** | 1.35 | 1.73 | 4.48E-05 | 0.0118 | 0.0294 | 0.0467 |
| **Creatinine** | 1.33 | 1.33 | 2.59E-06 | 0.0161 | 0.0266 | 0.0697 |
| **5-Methylthio-D-ribose** | 1.32 | 1.66 | 0.001 | 0.0019 | 0.0432 | 0.0527 |
| **L-Leucine** | 1.28 | 1.37 | 0.0005 | 0.0053 | 0.0247 | 0.018 |
| **L,L-TMAP** | 1.27 | 1.4 | 0.0003 | 0.0029 | 0.0357 | 0.0466 |
| **Pseudouridine** | 1.23 | 1.52 | 3.02E-07 | 0.0001 | 0.0157 | 0.0523 |
| **Ratio of pseudouridine to uridine** | 1.18 | 1.38 | 0.001 | 0.0063 | 0.0851 | 0.0542 |
| **Sulfotyrosine** | 1.15 | 1.28 | 0.0206 | 0.0189 | 0.0276 | 0.0987 |
| **Ratio of kynurenine to tryptophan** | 1.15 | 1.36 | 0.0214 | 0.0081 | 0.0241 | 0.0418 |
| **1,5-Anhydro-D-glucitol** | -8.97 | -2.26 | 1.25E-11 | 0.0197 | 0.0035 | 0.0451 |
|  |  |  |  |  |  |  |
| **Stage 3 *vs* healthy subjects** |  |  |  |  | 0.0768 | 0.0213 |
| **2-[3-(sulfooxy)phenyl]acetic acid** | 7.86 | 6.15 | 9.99E-10 | 1.71E-05 | 0.0245 | 0.0596 |
| **Hydroxybutyrylcarnitine** | 5.79 | 2.5 | 0.0002 | 1.43E-06 | 0.0983 | 0.0521 |
| **2-(*α*-D-Mannopyranosyl)-L-tryptophan** | 4.23 | 1.83 | 4.46E-14 | 1.96E-06 | 0.0782 | 0.0696 |
| ***O*-Adipoylcarnitine** | 4.22 | 5.53 | 2.39E-06 | 9.12E-07 | 0.0671 | 0.0751 |
| **Arabinose isomer** | 3.8 | 2.45 | 7.10E-12 | 2.54E-06 | 0.0014 | 0.007 |
| **N-Acetylcarnosine** | 3.11 | 1.93 | 0.0006 | 0.0039 | 0.0902 | 0.0063 |
| **4-Acetamidobutanoic acid** | 2.82 | 2.32 | 6.23E-14 | 3.99E-07 | 0.0208 | 0.0447 |
| **Kynurenic acid** | 2.6 | 3.05 | 2.83E-07 | 0.0001 | 0.0523 | 0.0862 |
| **5-Methylthio-D-ribose** | 2.48 | 2.22 | 2.97E-10 | 1.40E-06 | 0.0225 | 0.0329 |
| **Ratio of kynurenine to tryptophan** | 2.23 | 1.62 | 8.39E-11 | 0.0001 | 0.0837 | 0.0211 |
| **Succinyladenosine** | 2.2 | 1.95 | 1.85E-12 | 4.07E-07 | 0.0973 | 0.0561 |
| **Ratio of glutamic acid to glutamine** | 2.18 | 1.97 | 4.77E-07 | 0.0005 | 0.0069 | 0.0648 |
| **Homovanillic acid sulfate** | 2.09 | 3.72 | 0.0055 | 1.52E-06 | 0.0769 | 0.0162 |
| **L,L-TMAP** | 2.01 | 1.91 | 7.94E-10 | 1.25E-06 | 0.0635 | 0.0648 |
| **Ratio of kynurenic acid to kynurenine** | 1.97 | 1.91 | 0.0026 | 0.0029 | 0.0868 | 0.0036 |
| **2-Octenoylcarnitine** | 1.95 | 2.88 | 0.0019 | 0.0051 | 0.0248 | 0.0834 |
| **Sulfotyrosine** | 1.93 | 2 | 1.76E-09 | 7.44E-06 | 0.0212 | 0.0755 |
| **Urea (mmol/L)** | 1.91 | 1.61 | 5.01E-09 | 0.0124 | 0.0335 | 0.0042 |
| **Creatinine** | 1.88 | 1.78 | 4.34E-10 | 1.28E-05 | 0.0615 | 0.0685 |
| **L,L-TMAP isomer** | 1.88 | 2.09 | 5.13E-09 | 1.17E-07 | 0.053 | 0.018 |
| **Indole-3-lactic acid** | 1.87 | 1.63 | 8.09E-07 | 0.0002 | 0.0162 | 0.0663 |
| **Pseudouridine** | 1.84 | 2.24 | 4.94E-14 | 2.77E-07 | 0.0782 | 0.0373 |
| **Succinylcarnitine** | 1.79 | 2.52 | 2.25E-05 | 0.0002 | 0.0303 | 0.0412 |
| **L-Glutamic acid** | 1.73 | 1.78 | 2.31E-06 | 0.0016 | 0.0755 | 0.0618 |
| **Ratio of pseudouridine to uridine** | 1.72 | 2.15 | 1.14E-08 | 2.85E-06 | 0.0583 | 0.0364 |
| **Valerylcarnitine** | 1.66 | 1.46 | 6.70E-06 | 0.0261 | 0.0973 | 0.0115 |
| **Butyrylcarnitine** | 1.59 | 1.41 | 8.46E-06 | 0.044 | 0.0831 | 0.0954 |
| **L-Kynurenine** | 1.54 | 1.42 | 1.98E-07 | 0.0006 | 0.0766 | 0.0293 |
| **Cortisol** | 1.38 | 1.42 | 0.0288 | 0.0206 | 0.0979 | 0.0871 |
| **Choline** | 1.37 | 1.26 | 5.59E-07 | 0.0138 | 0.0448 | 0.0048 |
| **D-Glucose** | 1.22 | 1.43 | 0.031 | 4.68E-05 | 0.0559 | 0.0318 |
| **Uric acid** | 1.21 | 1.25 | 0.0011 | 0.0177 | 0.0585 | 0.0083 |
| **L-Leucine** | 1.2 | 1.3 | 0.0007 | 0.0192 | 0.0936 | 0.0625 |
| **L-Phenylalanine** | 1.18 | 1.28 | 0.0001 | 0.0013 | 0.0168 | 0.0193 |
| **L-Glutamine** | -1.24 | -1.16 | 1.27E-05 | 0.0035 | 0.0883 | 0.0556 |
| **Betaine** | -1.28 | -1.54 | 0.0048 | 0.0015 | 0.0912 | 0.0842 |
| **Ratio of tyrosine to phenylalanine** | -1.49 | -1.34 | 6.26E-06 | 0.0004 | 0.0866 | 0.0257 |
| **Ratio of glutamine to glutamic acid** | -2.18 | -2.05 | 4.77E-07 | 0.0005 | 0.0952 | 0.0025 |
| **1,5-Anhydro-D-glucitol** | -5.47 | -2.88 | 3.22E-07 | 0.0001 | 0.0836 | 0.0888 |
|  |  |  |  |  |  |  |
| **Stage 4 *vs* healthy subjects** |  |  |  |  |  |  |
| **2-[3-(sulfooxy)phenyl]acetic acid** | 139.84 | 267.82 | 1.96E-16 | 8.60E-12 | 0.0961 | 0.097 |
| ***p*-Cresol glucuronide** | 115.02 | 80.02 | 6.88E-12 | 0.0001 | 0.0639 | 0.0835 |
| **Homovanillic acid sulfate** | 36.66 | 73.94 | 1.50E-11 | 1.04E-08 | 0.0666 | 0.0326 |
| ***O*-Adipoylcarnitine** | 21.46 | 43.8 | 2.62E-13 | 1.29E-08 | 0.0126 | 0.0776 |
| **Arabinose isomer** | 20.08 | 24.29 | 7.28E-11 | 3.51E-08 | 0.0992 | 0.0378 |
| **L-*β*-aspartyl-L-leucine** | 14.67 | 8.44 | 2.42E-13 | 3.26E-06 | 0.0831 | 0.0742 |
| **Kynurenic acid** | 14.29 | 57.82 | 2.90E-11 | 6.14E-08 | 0.0619 | 0.0879 |
| **2-(*α*-D-Mannopyranosyl)-L-tryptophan** | 14.06 | 10.87 | 2.47E-16 | 5.27E-08 | 0.0946 | 0.0528 |
| ***N*-Acetylcarnosine** | 11.41 | 10.69 | 1.14E-11 | 3.14E-06 | 0.0355 | 0.0941 |
| **2-Hydroxyethanesulfonate** | 11.33 | 12.88 | 8.50E-12 | 1.00E-07 | 0.0728 | 0.0056 |
| ***α*-N-Phenylacetyl-L-glutamine** | 11.05 | 31.16 | 1.92E-11 | 2.83E-07 | 0.0549 | 0.0036 |
| **5-Methylthio-D-ribose** | 9.94 | 12.06 | 8.00E-18 | 3.92E-10 | 0.0293 | 0.0426 |
| **4-Acetamidobutanoic acid** | 9.88 | 36.26 | 5.32E-11 | 1.76E-09 | 0.0307 | 0.0914 |
| **Indoxyl sulfate** | 9.48 | 21.24 | 2.71E-10 | 3.22E-08 | 0.0085 | 0.0935 |
| **Succinyladenosine** | 9.13 | 10.67 | 9.46E-14 | 1.85E-09 | 0.0775 | 0.0118 |
| **Hydroxybutyrylcarnitine** | 9.07 | 4.5 | 0.0001 | 0.0002 | 0.0746 | 0.0934 |
| ***p*-Cresol sulfate** | 8.61 | 11.55 | 2.92E-06 | 3.41E-05 | 0.0565 | 0.088 |
| **Sulfotyrosine** | 7.6 | 8.34 | 7.50E-12 | 1.02E-11 | 0.0902 | 0.0888 |
| **Ratio of kynurenic acid to kynurenine** | 7.31 | 20.32 | 2.25E-08 | 1.55E-07 | 0.0582 | 0.0742 |
| **L,L-TMAP** | 7.29 | 12.58 | 2.44E-12 | 1.92E-09 | 0.0853 | 0.0522 |
| **Ratio of kynurenine to tryptophan** | 6.67 | 8.65 | 1.84E-11 | 2.37E-06 | 0.031 | 0.054 |
| **Ratio of pseudouridine to uridine** | 6.64 | 12.07 | 7.51E-13 | 7.23E-12 | 0.018 | 0.0598 |
| **Phenol sulfate** | 6.5 | 10.97 | 1.15E-07 | 1.07E-07 | 0.0627 | 0.0219 |
| **Pyrocatechol sulfate** | 6.32 | 4.9 | 5.31E-10 | 0.0207 | 0.0265 | 0.0906 |
| **Pseudouridine** | 5.17 | 12.36 | 2.11E-13 | 1.96E-11 | 0.0506 | 0.0735 |
| **L,L-TMAP isomer** | 5.16 | 11.91 | 3.41E-12 | 1.77E-09 | 0.0448 | 0.0461 |
| **Creatinine** | 4.39 | 9.01 | 1.03E-11 | 7.46E-10 | 0.0962 | 0.0227 |
| **Urea** | 4.05 | 3.44 | 2.73E-14 | 7.18E-09 | 0.0254 | 0.0933 |
| **Hippuric acid** | 3.38 | 6.08 | 0.0002 | 0.0118 | 0.0848 | 0.0978 |
| **Succinylcarnitine** | 3.22 | 5.52 | 7.75E-15 | 3.71E-07 | 0.0765 | 0.0788 |
| **Butyrylcarnitine** | 3.06 | 3.59 | 1.48E-10 | 0.0005 | 0.0539 | 0.025 |
| **Indole-3-lactic acid** | 2.89 | 2.95 | 1.04E-09 | 0.0001 | 0.0924 | 0.0207 |
| **2-Octenoylcarnitine** | 2.49 | 4.44 | 1.21E-06 | 0.0003 | 0.0835 | 0.005 |
| **L-Citrulline** | 2.3 | 2.43 | 1.25E-08 | 1.28E-06 | 0.0205 | 0.0667 |
| **Ratio of L-citrulline to arginine** | 2.12 | 2.35 | 0.0001 | 0.0002 | 0.0665 | 0.0907 |
| **L-Kynurenine** | 1.83 | 2.26 | 3.47E-10 | 8.51E-07 | 0.0044 | 0.0341 |
| **Valerylcarnitine** | 1.81 | 2.26 | 0.0002 | 0.0022 | 0.0247 | 0.0996 |
| **3-Indoleacetic acid** | 1.79 | 2.64 | 0.0003 | 9.34E-06 | 0.0826 | 0.0214 |
| **Ratio of glutamic acid to glutamine** | 1.7 | 1.98 | 5.21E-06 | 0.0182 | 0.0148 | 0.0779 |
| **L-Glutamic acid** | 1.63 | 1.62 | 1.59E-05 | 0.0438 | 0.0859 | 0.0757 |
| **Choline** | 1.57 | 1.31 | 2.89E-08 | 0.0006 | 0.0291 | 0.0368 |
| **9-Decenoylcarnitine** | 1.47 | 2.04 | 0.0059 | 0.0021 | 0.0638 | 0.0435 |
| **Citric acid** | 1.3 | 1.86 | 0.0027 | 1.22E-05 | 0.0785 | 0.0668 |
| **L-Phenylalanine** | 1.23 | 1.1 | 1.73E-06 | 0.046 | 0.0163 | 0.0527 |
| **Uric acid** | 1.18 | 1.37 | 0.0364 | 0.0087 | 0.0471 | 0.0134 |
| **Cortisol** | 1.13 | 2 | 0.033 | 4.35E-05 | 0.0677 | 0.0801 |
| **L-Glutamine** | -1.17 | -1.17 | 0.0146 | 0.0054 | 0.0166 | 0.019 |
| **Betaine** | -1.32 | -1.78 | 0.0122 | 0.0063 | 0.0442 | 0.0809 |
| **L-Tyrosine** | -1.59 | -1.58 | 1.86E-09 | 0.0002 | 0.0159 | 0.0256 |
| **Ratio of glutamine to glutamic acid** | -1.7 | -2.05 | 5.21E-06 | 0.0182 | 0.0225 | 0.0446 |
| **L-Carnitine** | -2.2 | -1.66 | 2.69E-08 | 0.0068 | 0.0286 | 0.0798 |
| **Ratio of tyrosine to phenylalanine** | -2.29 | -1.97 | 2.38E-12 | 7.91E-06 | 0.0914 | 0.0828 |
| **L-Tryptophan** | -2.93 | -5.26 | 4.66E-08 | 0.0001 | 0.0779 | 0.0772 |
| **Bilirubin** | -3.49 | -2.42 | 1.95E-09 | 0.0235 | 0.0318 | 0.0859 |
| **1,5-Anhydro-D-glucitol** | -4.88 | -7.15 | 7.64E-08 | 1.40E-06 | 0.0629 | 0.0931 |
|  |  |  |  |  |  |  |

*Fold change is the ratio of median at different stages over the healthy subjects (Stage 0) and the use of median is to minimize the effect of outliers. Negative sign indicates downward trend. P value is the Student’s t-test of log10-transformed data to minimize the variance. FDR is the false discovery rate.

Supplementary Table 6. Metabolites that had significant fold changes at different stages with respect to their later stage in both discovery and validation sets.

| **Metabolites** | **Fold change (median) *** | | ***P* value** | | ***FDR*** |  |
| --- | --- | --- | --- | --- | --- | --- |
|  | **Discovery set** | **Validation set** | **Discovery set** | **Validation set** | **Discovery set** | **Validation set** |
| **Stage 1b *vs* Stage 1a** |  |  |  |  |  |  |
| **L,L-TMAP** | 1.28 | 1.36 | 0.0005 | 0.0462 | 0.0962 | 0.0231 |
| **Creatinine** | 1.27 | 1.24 | 0.0349 | 0.0261 | 0.0681 | 0.0652 |
| ***γ*-Butyrobetaine** | 1.25 | 1.17 | 0.0012 | 0.0303 | 0.0149 | 0.0754 |
| **L,L-TMAP isomer** | 1.21 | 1.23 | 0.003 | 0.0413 | 0.0173 | 0.0344 |
| **Sulfotyrosine** | 1.21 | 1.43 | 0.0096 | 0.0138 | 0.0785 | 0.0767 |
| **Succinyladenosine** | 1.17 | 1.14 | 0.0095 | 0.0066 | 0.0945 | 0.0034 |
|  |  |  |  |  |  |  |
| **Stage 2 *vs* Stage 1b** |  |  |  |  |  |  |
| **2-(*α*-D-Mannopyranosyl)-L-tryptophan** | 1.7 | 1.5 | 0.0001 | 0.0003 | 0.0555 | 0.0474 |
| **5-Methylthio-D-ribose** | 1.49 | 1.52 | 0.0003 | 0.0019 | 0.052 | 0.0105 |
| **4-Acetamidobutanoic acid** | 1.42 | 1.59 | 0.0021 | 0.0008 | 0.0542 | 0.0016 |
| **Succinyladenosine** | 1.34 | 1.43 | 0.0089 | 0.0047 | 0.017 | 0.0288 |
| **Creatinine** | 1.27 | 1.31 | 0.0005 | 0.0294 | 0.0566 | 0.0981 |
| **Sulfotyrosine** | 1.21 | 1.28 | 0.0111 | 0.0308 | 0.094 | 0.0324 |
| **L,L-TMAP** | 1.21 | 1.31 | 0.0005 | 0.015 | 0.0273 | 0.0307 |
| **Ratio of pseudouridine to uridine** | 1.21 | 1.23 | 0.0384 | 0.014 | 0.061 | 0.0139 |
| **Pseudouridine** | 1.19 | 1.35 | 4.33E-05 | 0.0002 | 0.0286 | 0.0326 |
|  |  |  |  |  |  |  |
| **Stage 3 *vs* Stage 2** |  |  |  |  | 0.0517 | 0.0841 |
| ***O*-Adipoylcarnitine** | 3.62 | 3.33 | 1.59E-05 | 0.0124 | 0.0688 | 0.0181 |
| **2-(*α*-D-Mannopyranosyl)-L-tryptophan** | 2.04 | 1.28 | 1.28E-08 | 0.0194 | 0.0918 | 0.0751 |
| **5-Methylthio-D-ribose** | 1.88 | 1.34 | 2.34E-07 | 0.0144 | 0.0489 | 0.003 |
| **4-Acetamidobutanoic acid** | 1.75 | 1.47 | 7.44E-07 | 0.0025 | 0.0195 | 0.0067 |
| **L-Kynurenine** | 1.68 | 1.11 | 4.61E-07 | 0.0475 | 0.0717 | 0.0623 |
| **Indole-3-lactic acid** | 1.68 | 1.27 | 2.43E-07 | 0.0407 | 0.0558 | 0.0915 |
| **Sulfotyrosine** | 1.67 | 1.57 | 5.04E-07 | 0.0019 | 0.0472 | 0.0589 |
| **Arabinose isomer** | 1.59 | 1.44 | 0.0151 | 0.0393 | 0.0499 | 0.0291 |
| **L,L-TMAP** | 1.58 | 1.36 | 1.29E-06 | 0.0062 | 0.0012 | 0.0156 |
| **Pseudouridine** | 1.49 | 1.47 | 2.99E-09 | 0.0027 | 0.0805 | 0.035 |
| **Ratio of pseudouridine to uridine** | 1.46 | 1.56 | 0.0002 | 0.0157 | 0.0687 | 0.0582 |
| **Creatinine** | 1.42 | 1.34 | 3.60E-05 | 0.0122 | 0.005 | 0.0777 |
| **Succinyladenosine** | 1.42 | 1.45 | 4.72E-06 | 0.0063 | 0.0367 | 0.0081 |
| **L,L-TMAP isomer** | 1.38 | 1.38 | 0.0001 | 0.0047 | 0.0496 | 0.0752 |
| **Dehydroepiandrosterone sulfate** | -1.27 | -1.38 | 0.0401 | 0.0333 | 0.0929 | 0.0945 |
|  |  |  |  |  |  |  |
| **Stage 4 *vs* Stage 3** |  |  |  |  |  |  |
| **2-[3-(sulfooxy)phenyl]acetic acid** | 17.78 | 43.54 | 1.19E-09 | 1.69E-09 | 0.0164 | 0.0326 |
| **Homovanillic acid sulfate** | 17.54 | 19.86 | 1.74E-07 | 2.05E-06 | 0.0861 | 0.0495 |
| **p-Cresol glucuronide** | 6.43 | 53.75 | 1.39E-05 | 0.0003 | 0.0283 | 0.0193 |
| **Indoxyl sulfate** | 5.62 | 15.23 | 2.69E-07 | 2.28E-08 | 0.0966 | 0.0644 |
| **Kynurenic acid** | 5.49 | 18.97 | 3.35E-07 | 4.84E-06 | 0.0656 | 0.0301 |
| **Arabinose isomer** | 5.28 | 9.91 | 9.51E-07 | 2.10E-07 | 0.0304 | 0.0384 |
| ***O*-Adipoylcarnitine** | 5.08 | 7.93 | 3.04E-06 | 4.49E-06 | 0.0823 | 0.0372 |
| **Phenol sulfate** | 4.69 | 4.22 | 1.10E-06 | 1.16E-05 | 0.0389 | 0.0176 |
| **2-Hydroxyethanesulfonate** | 4.42 | 7.68 | 4.55E-08 | 2.54E-07 | 0.0492 | 0.0683 |
| **Succinyladenosine** | 4.15 | 5.46 | 2.97E-10 | 8.56E-12 | 0.0244 | 0.0977 |
| **5-Methylthio-D-ribose** | 4.01 | 5.43 | 2.30E-11 | 8.06E-08 | 0.0885 | 0.0745 |
| ***α*-N-Phenylacetyl-L-glutamine** | 3.97 | 11.58 | 1.44E-08 | 2.97E-06 | 0.0498 | 0.0533 |
| **Sulfotyrosine** | 3.94 | 4.17 | 1.40E-08 | 3.22E-09 | 0.0077 | 0.0494 |
| **N-Acetylcarnosine** | 3.67 | 5.53 | 1.00E-06 | 0.0002 | 0.0637 | 0.0199 |
| **L,L-TMAP** | 3.63 | 6.59 | 1.21E-08 | 2.78E-07 | 0.0107 | 0.0088 |
| **L-*β*-aspartyl-L-leucine** | 3.52 | 5.28 | 3.91E-08 | 2.52E-05 | 0.0832 | 0.034 |
| **4-Acetamidobutanoic acid** | 3.51 | 15.61 | 3.50E-07 | 7.30E-08 | 0.0482 | 0.0236 |
| **2-(*α*-D-Mannopyranosyl)-L-tryptophan** | 3.32 | 5.94 | 2.12E-08 | 2.97E-07 | 0.0899 | 0.0328 |
| **Pseudouridine** | 2.81 | 5.53 | 1.98E-09 | 1.84E-09 | 0.0407 | 0.0734 |
| **L,L-TMAP isomer** | 2.75 | 5.71 | 2.50E-08 | 2.91E-07 | 0.0691 | 0.0261 |
| **Pyrocatechol sulfate** | 2.42 | 4.26 | 0.0067 | 0.0135 | 0.034 | 0.0557 |
| **MS-detected creatinine** | 2.33 | 5.06 | 4.73E-08 | 1.27E-07 | 0.0675 | 0.0831 |
| ***p*-Cresol sulfate** | 2.33 | 13.03 | 0.01 | 1.49E-05 | 0.0711 | 0.0383 |
| **Urea** | 2.12 | 2.13 | 1.71E-08 | 1.21E-06 | 0.0862 | 0.0553 |
| **Butyrylcarnitine** | 1.92 | 2.54 | 2.39E-05 | 0.0039 | 0.0979 | 0.0549 |
| **Hippuric acid** | 1.91 | 4.83 | 0.0184 | 0.0067 | 0.0761 | 0.0218 |
| **Succinylcarnitine** | 1.8 | 2.19 | 7.46E-06 | 0.0002 | 0.0549 | 0.0214 |
| **3-Hydroxydecanoyl carnitine** | 1.65 | 1.95 | 0.0393 | 0.0037 | 0.0991 | 0.0421 |
| **3-Indoleacetic acid** | 1.38 | 2.4 | 0.0051 | 0.0001 | 0.0205 | 0.0201 |
| **L-Citrulline** | 1.25 | 2.19 | 0.0038 | 4.88E-05 | 0.0348 | 0.0306 |
| **Citric acid** | 1.21 | 1.56 | 0.0365 | 0.0009 | 0.0017 | 0.0167 |
| **L-Kynurenine** | 1.19 | 1.6 | 0.0128 | 0.0004 | 0.0441 | 0.0061 |
| **Uracil** | -1.14 | -1.27 | 0.0484 | 0.0423 | 0.0623 | 0.0655 |
| **L-Leucine** | -1.25 | -1.31 | 2.97E-06 | 0.0172 | 0.0053 | 0.0062 |
| **L-Tyrosine** | -1.31 | -1.84 | 4.60E-05 | 0.0008 | 0.0083 | 0.0394 |
| **L-Valine** | -1.47 | -1.08 | 1.51E-07 | 0.0392 | 0.0412 | 0.0588 |
| **L-Carnitine** | -1.98 | -1.49 | 1.04E-06 | 0.029 | 0.0907 | 0.0118 |
| **Bilirubin** | -1.99 | -2.83 | 0.0003 | 0.0376 | 0.0372 | 0.0134 |
| **L-Tryptophan** | -2.2 | -4.43 | 2.43E-06 | 0.0001 | 0.0582 | 0.0727 |
| **Ratio of pseudouridine to uridine** | 3.86 | 5.61 | 1.86E-09 | 4.68E-09 | 0.05 | 0.0596 |
| **Ratio of kynurenic acid to kynurenine** | 3.7 | 10.62 | 3.70E-06 | 3.61E-06 | 0.0896 | 0.0434 |
| **Ratio of kynurenine to tryptophan** | 2.99 | 5.34 | 1.80E-07 | 1.74E-05 | 0.0447 | 0.0733 |
| **Ratio of tyrosine to phenylalanine** | -1.54 | -1.47 | 0.0004 | 0.0178 | 0.0221 | 0.0736 |

*Fold change is the ratio of median at different stages over the healthy subjects (Stage 0) and the use of median is to minimize the effect of outliers. Negative sign indicates downward trend. *P* value is the Student’s t-test of log10-transformed data to minimize the variance. FDR is the false discovery rate.

**Supplementary Table 10. Description of 106 follow-up information.**

| **group** | **sex** | **age** | **ADT** | **succinyladenosine** | **pseudouridine** | **UACR** | **EPI eGFR before** | **EPI eGFR new** | **Cr before** | **Cr new** | **follow-up time(years)** |
| --- | --- | --- | --- | --- | --- | --- | --- | --- | --- | --- | --- |
| A | M | 67.29 | 96.98003472 | 27.47467076 | 264.4421378 | 8.377993348 | 60.10728519 | 70.63570191 | 94 | 93 | 4.282191781 |
| A | F | 78.53 | 71.16574914 | 17.8639968 | 218.3198715 | 1.442370796 | 60.83647793 | 77.08692004 | 69 | 64 | 4.663013699 |
| A | M | 73.65 | 97.92675915 | 25.70419242 | 385.2282727 | 2.99719762 | 61.40588816 | 84.2148331 | 89 | 74 | 3.967123288 |
| A | M | 58.98 | 99.3687654 | 36.13677776 | 296.0229552 | 412.0555686 | 63.71781694 | 72.16582606 | 94 | 96 | 4.071232877 |
| A | M | 59.52 | 63.1801847 | 23.69538435 | 256.3395969 | 5.905942602 | 65.15005218 | 80.71910507 | 92 | 87 | 4.531506849 |
| A | M | 54.38 | 81.5152861 | 31.09273025 | 276.2687258 | 1.294796395 | 65.81037186 | 74.88781853 | 94 | 95 | 5.202739726 |
| A | F | 57.30 | 84.41003405 | 26.97928496 | 331.6054194 | 8.482577057 | 67.07754197 | 95.26076355 | 72 | 58 | 4.561643836 |
| A | M | 61.32 | 78.47515107 | 20.82743067 | 286.9927175 | 9.734200214 | 67.88597568 | 80.10163986 | 88 | 87 | 3.830136986 |
| A | M | 61.43 | 62.44408816 | 18.79208875 | 223.8996179 | 3.107511347 | 68.77487236 | 82.36724717 | 87 | 86 | 1.734246575 |
| A | M | 63.12 | 80.48441006 | 23.95823413 | 223.210514 | 3.650968506 | 68.91830059 | 81.10215462 | 86 | 85 | 4.257534247 |
| A | M | 52.04 | 71.13090534 | 37.4354484 | 180.4400521 | 1.106672771 | 69.57921355 | 98.24956198 | 91 | 72 | 5.24109589 |
| A | F | 64.20 | 101.7411026 | 26.81887541 | 384.9284484 | 146.2799271 | 69.71561187 | 94.91616905 | 67 | 51 | 4.205479452 |
| A | M | 51.28 | 81.61233386 | 27.88381184 | 314.4196407 | 2.495390772 | 69.9524777 | 82.81543606 | 91 | 89 | 5.21369863 |
| A | M | 41.67 | 68.27550174 | 21.48132303 | 291.663502 | 6.871813197 | 70.15180851 | 100.5992486 | 96 | 81 | 3.342465753 |
| A | M | 68.65 | 99.19880625 | 27.03612031 | 243.811477 | 10.10604921 | 70.22505076 | 84.79170019 | 82 | 79 | 4.175342466 |
| A | M | 63.84 | 90.86434766 | 28.89173488 | 226.3904349 | 5.717051861 | 70.54907402 | 88.49397384 | 84 | 77 | 4.394520548 |
| A | M | 60.27 | 60.79378945 | 15.58768695 | 270.8628073 | 5.380644745 | 71.3129264 | 82.59224505 | 85 | 85 | 4.515068493 |
| A | M | 48.17 | 83.49182998 | 23.98165011 | 187.227846 | 4.618362244 | 72.45607731 | 92.12171105 | 90 | 83 | 5.169863014 |
| A | M | 48.16 | 120.4632841 | 26.39019293 | 330.6327221 | 94.19664351 | 72.46165534 | 81.04359469 | 90 | 93 | 3.84109589 |
| A | M | 55.68 | 68.2777298 | 19.00494137 | 281.9897321 | 30.61739483 | 72.61513284 | 73.7396642 | 86 | 96 | 4.298630137 |
| A | F | 52.98 | 80.84414414 | 22.61556826 | 214.6842911 | 13.18186057 | 72.79409265 | 99.27153695 | 69 | 56 | 4.652054795 |
| A | F | 53.53 | 88.74342406 | 26.87909391 | 371.5541092 | 153.9897134 | 73.8056769 | 73.42085786 | 68 | 77 | 4.769863014 |
| A | M | 43.39 | 70.3058864 | 28.69178341 | 282.7149935 | 1.666753973 | 73.9377834 | 91.97197852 | 91 | 86 | 4.073972603 |
| A | M | 68.97 | 86.89278047 | 23.91177657 | 355.0763788 | 3.861474507 | 75.60307366 | 81.99523491 | 77 | 81 | 5.147945205 |
| A | F | 75.01 | 72.08862249 | 26.23673078 | 209.46254 | 16.03103609 | 82.0287476 | 83.95704374 | 55 | 59 | 4.032876712 |
| A | M | 58.64 | 69.17041033 | 30.53905907 | 211.5671506 | 24.81173005 | 73.17796034 | 68.61928111 | 84 | 100 | 4.567123288 |
| A | M | 41.63 | 75.61920591 | 24.80825151 | 235.2523252 | 51.37074759 | 74.85843857 | 71.99537455 | 91 | 106 | 4.709589041 |
| A | F | 65.71 | 82.35659333 | 29.06637645 | 372.5098456 | 33.65074398 | 59.23050913 | 57.50535951 | 76 | 88 | 4.394520548 |
| A | F | 59.35 | 115.7714625 | 36.29859956 | 457.3835696 | 63.18891836 | 66.12010688 | 64.33061668 | 72 | 83 | 4.854794521 |
| A | M | 56.92 | 79.83759533 | 24.99954162 | 247.6576797 | 73.58011585 | 73.01134779 | 71.09155695 | 85 | 98 | 4.717808219 |
| A | F | 79.69 | 73.42611304 | 21.10567957 | 283.0892225 | 2.68920838 | 85.20323713 | 83.33856056 | 48 | 54 | 4.550684932 |
| A | M | 57.22 | 73.44908242 | 27.05318412 | 210.1608773 | 8.342728649 | 70.83828267 | 62.06378239 | 87 | 110 | 3.871232877 |
| B | M | 53.10 | 222.5610087 | 65.16951978 | 1024.364786 | 4876.740609 | 60.88374883 | 34.20260815 | 101 | 188 | 0.575342466 |
| B | M | 79.30 | 68.48559317 | 23.83466068 | 309.013184 | 4211.057117 | 83.13986884 | 3.698191958 | 64 | 1008 | 2.019178082 |
| B | M | 45.47 | 166.5226777 | 34.36635145 | 682.0604272 | 5953.808362 | 83.87744455 | 9.335445277 | 81 | 570 | 2.150684932 |
| B | M | 30.25 | 91.68760206 | 25.84866441 | 439.9470206 | 2360.589058 | 64.47476272 | 7.900166253 | 110 | 714 | 2.367123288 |
| B | F | 78.59 | 105.3129504 | 26.54905175 | 332.1680089 | 12557.32329 | 75.01779394 | 43.89919993 | 58 | 104 | 1.194520548 |
| B | F | 58.78 | 102.091826 | 35.44393612 | 437.9217034 | 5561.997137 | 73.74655051 | 13.72711603 | 66 | 303 | 2.449315068 |
| B | F | 80.76 | 135.2075542 | 28.86045256 | 575.0273104 | 371.6090673 | 60.95612996 | 24.96295411 | 68 | 163 | 2.043835616 |
| B | M | 75.81 | 104.3836901 | 45.13836144 | 466.9393633 | 36.56282041 | 68.8032288 | 23.08828883 | 80 | 225 | 2.887671233 |
| B | F | 53.95 | 87.40101179 | 27.95761755 | 252.0778443 | 318.031722 | 59.5613051 | 4.854252728 | 81 | 727 | 4.638356164 |
| B | F | 64.65 | 89.34133969 | 21.88491307 | 299.7353593 | 131.4949429 | 74.86249386 | 32.44620656 | 63 | 143 | 3.35890411 |
| B | M | 73.71 | 88.96409916 | 33.85770315 | 303.5859645 | 361.3882924 | 63.98036941 | 34.07171348 | 86 | 165 | 2.97260274 |
| B | M | 76.94 | 125.4535186 | 27.14704491 | 371.8481956 | 3.127971573 | 71.48534581 | 35.94505821 | 77 | 154 | 3.991780822 |
| B | F | 61.44 | 122.2684061 | 30.77465844 | 310.0702587 | 913.1274266 | 72.38539537 | 37.73290981 | 66 | 128 | 4.15890411 |
| B | F | 72.15 | 87.72542929 | 29.61664776 | 210.8507815 | 6.48326518 | 76.88598667 | 49.85860622 | 59 | 96 | 3.293150685 |
| B | F | 72.16 | 105.5684714 | 30.75317265 | 367.5122436 | 4.931440646 | 72.40422851 | 51.23350194 | 62 | 93 | 4.871232877 |
| B | F | 62.88 | 96.25035884 | 42.63140323 | 232.6694526 | 1068.286866 | 64.49877092 | 49.86771514 | 72 | 101 | 3.791780822 |
| B | F | 61.31 | 94.20978399 | 19.00449784 | 367.0924675 | 1075.824392 | 64.13548025 | 45.48262526 | 73 | 109 | 5.345205479 |
| C | F | 62.95 | 153.7493071 | 55.18200788 | 793.4000377 | 27.2980529 | 26.75881732 | 34.10897754 | 149 | 138 | 4.068493151 |
| C | F | 71.86 | 159.4567236 | 77.21296486 | 451.8763472 | #REF! | 27.82208812 | 30.06744928 | 137 | 146 | 3.419178082 |
| C | M | 59.24 | 154.1365903 | 64.68586349 | 418.6756256 | 2.816158838 | 31.29416772 | 66.39523429 | 169 | 102 | 5.24109589 |
| C | F | 46.73 | 136.0283035 | 38.87690042 | 537.6030773 | 676.5974735 | 31.78617437 | 32.30271425 | 142 | 158 | 4.747945205 |
| C | M | 63.35 | 113.7118407 | 40.74013768 | 323.5557024 | 351.193594 | 33.75437521 | 51.05284125 | 155 | 124 | 4.923287671 |
| C | M | 40.98 | 129.7618694 | 39.29212808 | 488.17285 | 716.076582 | 33.87554174 | 50.40188487 | 176 | 143 | 4.591780822 |
| C | M | 72.90 | 151.448399 | 54.80785329 | 503.8759482 | 2268.554577 | 34.79456563 | 41.74987953 | 143 | 139 | 4.35890411 |
| C | F | 71.54 | 127.5961819 | 36.55410754 | 558.3225527 | 267.7470268 | 37.59566857 | 46.48945121 | 107 | 102 | 3.42739726 |
| C | M | 69.93 | 125.3400987 | 40.85051464 | 379.6613921 | 23.74780647 | 39.13729101 | 44.0684224 | 132 | 135 | 4.657534247 |
| C | M | 81.88 | 145.4393403 | 41.39615597 | 396.0792953 | 4.139564281 | 39.58193662 | 45.8910246 | 122 | 122 | 4.364383562 |
| C | F | 61.87 | 95.65842523 | 27.46682549 | 283.6749903 | 65.50274207 | 43.14373227 | 55.68923594 | 101 | 94 | 1.443835616 |
| C | F | 68.37 | 154.1027965 | 40.03720057 | 546.2718399 | 81.44647015 | 44.38798445 | 61.42262941 | 95 | 82 | 4.506849315 |
| C | F | 43.41 | 101.469182 | 42.55593158 | 326.8664818 | 237.7900418 | 45.80821118 | 48.13098212 | 107 | 117 | 3 |
| C | F | 77.98 | 126.8375878 | 34.95639714 | 425.079464 | 23.0568587 | 46.79368774 | 58.5494953 | 86 | 81 | 3.821917808 |
| C | M | 66.70 | 112.27474 | 38.63061237 | 462.1209398 | 15.19701465 | 46.80648163 | 54.23578939 | 116 | 116 | 4.446575342 |
| C | M | 72.40 | 70.2638843 | 24.04021084 | 240.426215 | 21.97638773 | 48.48439331 | 50.84549825 | 109 | 119 | 3.542465753 |
| C | F | 60.12 | 89.91198255 | 22.72925412 | 198.6925976 | 34.412557 | 50.89741368 | 80.12036755 | 89 | 69 | 4.635616438 |
| C | F | 79.10 | 63.01252418 | 21.53485098 | 177.0889041 | 8.363588219 | 51.44635281 | 65.50358827 | 79 | 73 | 4.624657534 |
| C | M | 73.25 | 93.11127281 | 29.70688379 | 291.0874991 | 53.95969653 | 52.22100532 | 53.07674052 | 102 | 114 | 3.961643836 |
| C | F | 80.15 | 94.44395522 | 44.96736483 | 277.0442007 | 10565.66619 | 53.51566328 | 67.55604522 | 76 | 71 | 3.964383562 |
| C | M | 74.73 | 102.5973753 | 29.85144479 | 272.5490805 | 1.384921254 | 54.24321572 | 63.66164548 | 98 | 97 | 4.391780822 |
| C | M | 61.65 | 143.9286505 | 48.55991741 | 591.6922759 | 13.96576667 | 54.70542637 | 73.37929709 | 105 | 93 | 4.498630137 |
| C | M | 66.07 | 74.326534 | 22.47386929 | 265.5575751 | 5.475117919 | 55.58299121 | 68.32072116 | 101 | 96 | 4.783561644 |
| C | F | 61.55 | 98.76282147 | 26.35440117 | 417.2995843 | 513.9713158 | 55.63344251 | 63.26002277 | 82 | 83 | 5.043835616 |
| C | M | 76.40 | 90.61305624 | 29.11853701 | 440.5719902 | 5.359610366 | 55.66361384 | 68.63477094 | 95 | 90 | 4.904109589 |
| C | F | 76.52 | 118.3530053 | 37.19961582 | 303.9674893 | 41.53834286 | 57.63706447 | 72.73503385 | 73 | 68 | 4.509589041 |
| C | F | 72.14 | 136.0757288 | 38.19760696 | 438.9914339 | 23.85627785 | 54.86220488 | 49.68524009 | 78 | 97 | 2.008219178 |
| C | M | 59.18 | 113.3580696 | 31.06111139 | 268.7168385 | 25.81056881 | 53.20025042 | 50.00715564 | 109 | 129 | 5.235616438 |
| C | F | 67.07 | 85.58571462 | 20.5438559 | 353.5924419 | 7.426044497 | 59.61254128 | 57.5009328 | 75 | 88 | 3.04109589 |
| C | M | 75.68 | 96.3551802 | 34.93118823 | 257.7541995 | 6.052832802 | 47.37840716 | 43.9510805 | 109 | 131 | 4.465753425 |
| C | F | 73.85 | 181.166315 | 51.23860118 | 779.6592516 | 100.4108477 | 39.19396584 | 37.91999533 | 102 | 119 | 3.591780822 |
| C | F | 56.82 | 120.0956037 | 26.10825998 | 424.7858566 | 109.3337439 | 50.70768382 | 45.03403434 | 91 | 113 | 5.043835616 |
| C | M | 86.18 | 102.0158826 | 29.30419741 | 423.5317574 | 651.8234096 | 52.63671834 | 45.45245253 | 94 | 121 | 2.852054795 |
| C | F | 79.31 | 94.25087465 | 42.40376179 | 286.5867944 | 11.15214123 | 52.98971596 | 46.44959904 | 77 | 97 | 4.430136986 |
| C | M | 82.44 | 143.6752167 | 40.70511793 | 367.3224016 | 3.039440694 | 49.5423332 | 43.26130097 | 101 | 127 | 5.293150685 |
| D | M | 74.53 | 202.1736261 | 45.12539357 | 265.9061994 | 3319.997908 | 30.25871092 | 15.42706673 | 159 | 320 | 0.942465753 |
| D | M | 48.81 | 105.5715417 | 21.73976468 | 354.2531158 | 1023.99826 | 44.5805275 | 13.83164088 | 134 | 405 | 1.663013699 |
| D | M | 58.07 | 217.1933131 | 56.91831448 | 884.8114944 | 2290.594588 | 56.09785388 | 7.766384343 | 105 | 617 | 2.109589041 |
| D | M | 43.84 | 139.3722025 | 41.49550078 | 406.1118293 | 243.7970769 | 50.70061034 | 6.784400765 | 124 | 747 | 2.671232877 |
| D | M | 62.07 | 119.3842662 | 38.32951387 | 404.2622057 | 2996.928377 | 50.44751726 | 9.152908144 | 112 | 523 | 3.167123288 |
| D | F | 62.96 | 126.94128 | 27.98871025 | 323.8975285 | 80.49139173 | 53.50307461 | 19.19094021 | 84 | 224 | 2.567123288 |
| D | F | 78.99 | 107.8580199 | 36.77863672 | 263.2552274 | 256.5060599 | 51.48696342 | 14.55129061 | 79 | 256 | 2.953424658 |
| D | F | 60.04 | 165.6632016 | 35.1591601 | 441.4261499 | 290.8227854 | 58.81698401 | 38.09264694 | 79 | 130 | 1.534246575 |
| D | M | 72.69 | 130.9746321 | 42.66625455 | 725.7884098 | 2362.561711 | 33.70243314 | 17.69558562 | 147 | 286 | 2.583561644 |
| D | M | 76.35 | 124.2908434 | 40.15034968 | 465.1275971 | 1797.11633 | 34.84409976 | 13.61923225 | 140 | 346 | 3.424657534 |
| D | F | 63.41 | 147.5225926 | 32.05524214 | 576.691318 | 12734.23202 | 42.68042634 | 11.1808785 | 101 | 346 | 4.189041096 |
| D | M | 65.64 | 96.33168505 | 33.81615072 | 468.2812573 | 1835.43426 | 49.20007322 | 17.54608777 | 112 | 297 | 4.350684932 |
| D | M | 57.75 | 95.67318107 | 27.22325105 | 265.9478454 | 727.0168856 | 44.24950857 | 17.4248754 | 128 | 312 | 4.742465753 |
| D | F | 67.03 | 118.2798456 | 32.70014747 | 392.7015279 | 190.5788155 | 44.24316183 | 25.86005556 | 96 | 170 | 3.512328767 |
| D | F | 77.15 | 87.88012711 | 28.6637533 | 256.2467669 | 22.30266149 | 57.38470834 | 27.60896061 | 73 | 151 | 4.479452055 |
| D | M | 63.44 | 117.7434707 | 35.44514154 | 234.4706083 | 2.200044148 | 53.40462163 | 32.86032355 | 106 | 180 | 3.416438356 |
| D | M | 81.98 | 105.2366213 | 34.20070412 | 265.6897598 | 16.95935852 | 54.21281536 | 34.52021811 | 94 | 155 | 3.597260274 |
| D | F | 59.20 | 139.0930518 | 45.05570984 | 658.5158439 | 536.8053483 | 36.80059276 | 19.83069747 | 117 | 220 | 4.756164384 |
| D | M | 51.29 | 107.3139883 | 38.85723422 | 335.3708145 | 277.8381877 | 41.54944247 | 25.71679238 | 140 | 235 | 4.569863014 |
| D | M | 64.33 | 132.3222942 | 36.68463281 | 354.5184079 | 1325.269397 | 32.50759229 | 23.84533343 | 159 | 232 | 4.504109589 |
| D | F | 58.04 | 156.5979099 | 47.18653694 | 747.8688118 | 366.975354 | 30.12466013 | 23.33964237 | 139 | 193 | 5.257534247 |
| D | F | 69.05 | 130.2797985 | 35.64409233 | 452.111145 | 1831.464572 | 29.6809764 | 13.05945004 | 132 | 294 | 4.471232877 |

**F: female; M:male**

Supplementary Table 13. List of ID in online database for networking and pathway analysis.

| **Metabolites** | PubChem ID | HMDB ID | KEGG ID | SMILES code |
| --- | --- | --- | --- | --- |
| 1,5-Anhydro-D-glucitol | 64960 | HMDB0002712 | C07326 | C1[C@@H]([C@H]([C@@H]([C@H](O1)CO)O)O)O |
| 2-[3-(sulfooxy)phenyl] acetic acid | 131831698 | HMDB0125163 | - | C1=CC(=CC(=C1)OS(=O)(=O)O)CC(=O)O |
| 2-(*α*-D-Mannopyranosyl)-L-tryptophan | 10981970 | HMDB0240296 | - | N[C@@H](CC1=C(NC2=C1C=CC=C2)[C@H]1O[C@H](CO)[C@@H](O)[C@H](O)[C@@H]1O)C(O)=O |
| 2-Hydroxybutyric acid | 11266 | HMDB0000008 | C05984 | CCC(C(=O)O)O |
| 2-Hydroxyethanesulfonate | 7866 | HMDB0003903 | C05123 | OCCS(O)(=O)=O |
| 2-Octenoylcarnitine | 53481667 | HMDB0013324 | - | CCCCC/C=C/C(=O)O[C@@H](CCC(=O)[O-])[N+](C)(C)C |
| 3,5-Tetradecadiencarnitine | 53481681 | HMDB0013331 | - | CCCCCCCC/C=C/C=C/CC(=O)O[C@@H](CCC(=O)[O-])[N+](C)(C)C |
| 3-Hydroxydecanoyl carnitine | 121454166 | HMDB0061636 | - | CCCCCCCC(CC(=O)OC(CC(=O)[O-])C[N+](C)(C)C)O |
| 3-Indoleacetic acid | 802 | HMDB0000197 | C00954 | C1=CC=C2C(=C1)C(=CN2)CC(=O)O |
| 4-Acetamidobutanoic acid | 18189 | HMDB0003681 | C02946 | CC(=O)NCCCC(=O)O |
| 5-Methylthio-D-ribose | 439904 | HMDB0001087 | C03089 | CSC[C@@H]1[C@H]([C@H]([C@@H](O1)O)O)O |
| 9-Decenoylcarnitine | 53481651 | HMDB0013205 | - | C[N+](C)(C)CC(CC(=O)[O-])OC(=O)CCCCCCCC=C |
| Betaine | 247 | HMDB0000043 | C00719 | C[N+](C)(C)CC(=O)[O-] |
| Bilirubin | 5280352 | HMDB0000054 | C00486 | CC1=C(C=C)\C(NC1=O)=C\C1=C(C)C(CCC(O)=O)=C(CC2=C(CCC(O)=O)C(C)=C(N2)\C=C2/NC(=O)C(C=C)=C2C)N1 |
| Butyrylcarnitine | 213144 | HMDB0002013 | C02862 | CCCC(=O)O[C@H](CC(=O)[O-])C[N+](C)(C)C |
| Choline | 305 | HMDB0000097 | C00114 | C[N+](C)(C)CCO |
| *cis*-5-Tetradecenoylcarnitine | 22833575 | HMDB0002014 | - | CCCCCCCC/C=C\CCCC(=O)OC(CC(=O)[O-])C[N+](C)(C)C |
| Citric acid | 311 | HMDB0000094 | C00158 | OC(=O)CC(O)(CC(O)=O)C(O)=O |
| Cortisol | 5754 | HMDB0000063 | C00735 | C[C@]12CCC(=O)C=C1CC[C@@H]3[C@@H]2[C@H](C[C@]4([C@H]3CC[C@@]4(C(=O)CO)O)C)O |
| Creatinine | 588 | HMDB0000562 | C00791 | CN1CC(=O)N=C1N |
| Decanoylcarnitine | 10245190 | HMDB0000651 | - | CCCCCCCCCC(=O)OC(CC(=O)[O-])C[N+](C)(C)C |
| Dehydroepiandrosterone sulfate | 12594 | HMDB0001032 | C04555 | C[C@]12CC[C@H]3[C@H]([C@@H]1CCC2=O)CC=C4[C@@]3(CC[C@@H](C4)OS(=O)(=O)O)C |
| D-Glucose | 5793 | HMDB0000122 | C00031 | C([C@@H]1[C@H]([C@@H]([C@H](C(O1)O)O)O)O)O |
| Dodecanoylcarnitine | 168381 | HMDB0002250 | - | CCCCCCCCCCCC(=O)O[C@H](CC(=O)[O-])C[N+](C)(C)C |
| Hexanoylcarnitine | 6426853 | HMDB0000705 | - | CCCCCC(=O)OC(CC([O-])=O)C[N+](C)(C)C |
| Hippuric acid | 464 | HMDB0000714 | C01586 | C1=CC=C(C=C1)C(=O)NCC(=O)O |
| Homovanillic acid sulfate | 29981063 | HMDB0011719 | - | COC1=C(C=CC(=C1)CC(=O)O)OS(=O)(=O)O |
| Hydroxybutyrylcarnitine | 53481617 | HMDB0013127 | - | CC(CC(=O)O[C@@H](CC(=O)[O-])C[N+](C)(C)C)O |
| Indole-3-lactic acid | 92904 | HMDB0000671 | C02043 | C1=CC=C2C(=C1)C(=CN2)CC(C(=O)O)O |
| Indoxyl sulfate | 10258 | HMDB0000682 | - | C1=CC=C2C(=C1)C(=CN2)OS(=O)(=O)O |
| Inosine | 6021 | HMDB0000195 | C00294 | C1=NC(=O)C2=C(N1)N(C=N2)[C@H]3[C@@H]([C@@H]([C@H](O3)CO)O)O |
| Kynurenic acid | 3845 | HMDB0000715 | C01717 | OC(=O)C1=CC(=O)C2=CC=CC=C2N1 |
| L,L-TMAP | 134218393 | HMDB0240365 | - | C[C@@H](C(=O)N1CCC[C@H]1C([O-])=O)[N+](C)(C)C |
| L-Acetylcarnitine | 7045767 | HMDB0000201 | C02571 | CC(=O)O[C@H](CC(=O)[O-])C[N+](C)(C)C |
| L-Arginine | 6322 | HMDB0000517 | C00062 | C(C[C@@H](C(=O)O)N)CN=C(N)N |
| L-Carnitine | 10917 | HMDB0000062 | C00318 | C[N+](C)(C)C[C@@H](CC(=O)[O-])O |
| L-Citrulline | 9750 | HMDB0000904 | C00327 | C(C[C@@H](C(=O)O)N)CNC(=O)N |
| L-Glutamic acid | 33032 | HMDB0000148 | C00025 | C(CC(=O)O)[C@@H](C(=O)O)N |
| L-Glutamine | 5961 | HMDB0000641 | C00064 | C(CC(=O)N)[C@@H](C(=O)O)N |
| L-Homocysteine | 91552 | HMDB0000742 | C00155 | N[C@@H](CCS)C(O)=O |
| L-Kynurenine | 161166 | HMDB0000684 | C00328 | N[C@@H](CC(=O)C1=CC=CC=C1N)C(O)=O |
| L-Leucine | 6106 | HMDB0000687 | C00123 | CC(C)C[C@H](N)C(O)=O |
| L-Methionine | 6137 | HMDB0000696 | C00073 | CSCC[C@@H](C(=O)O)N |
| L-Octanoylcarnitine | 11953814 | HMDB0000791 | C02838 | CCCCCCCC(=O)O[C@H](CC(=O)[O-])C[N+](C)(C)C |
| L-Ornithine | 6262 | HMDB0000214 | C00077 | C(C[C@@H](C(=O)O)N)CN |
| L-Phenylalanine | 6140 | HMDB0000159 | C00079 | C1=CC=C(C=C1)C[C@@H](C(=O)O)N |
| L-Proline | 145742 | HMDB0000162 | C00148 | C1C[C@H](NC1)C(=O)O |
| L-Threonine | 6288 | HMDB0000167 | C00188 | C[C@H]([C@@H](C(=O)O)N)O |
| L-Tryptophan | 6305 | HMDB0000929 | C00078 | C1=CC=C2C(=C1)C(=CN2)C[C@@H](C(=O)O)N |
| L-Tyrosine | 6057 | HMDB0000158 | C00082 | C1=CC(=CC=C1C[C@@H](C(=O)O)N)O |
| L-Valine | 6287 | HMDB0000883 | C00183 | CC(C)[C@@H](C(=O)O)N |
| LysoPE(18:1(11Z)/0:0) | 53480949 | HMDB0011505 | - | [H][C@@](O)(COC(=O)CCCCCCCCC\C=C/CCCCCC)COP(O)(=O)OCCN |
| L-*β*-aspartyl-L-leucine | 3549397 | HMDB0011166 | - | CC(C)CC(NC(=O)CC(N)C(O)=O)C(O)=O |
| *N*-Acetylcarnosine | 10221026 | HMDB0012881 | - | CC(=O)NCCC(=O)N[C@H](CC1=CN=CN1)C(=O)O |
| *O*-Adipoylcarnitine | 71296139 | HMDB0061677 | - | C[N+](C)(C)C[C@@H](CC([O-])=O)OC(=O)CCCCC(O)=O |
| *p*-Cresol glucuronide | 154035 | HMDB0011686 | - | CC1=CC=C(C=C1)O[C@H]2[C@@H]([C@H]([C@@H]([C@H](O2)C(=O)O)O)O)O |
| *p*-Cresol sulfate | 4615423 | HMDB0011635 | - | CC1=CC=C(C=C1)OS(=O)(=O)O |
| Phenol sulfate | 74426 | HMDB0060015 | C02180 | C1=CC=C(C=C1)OS(=O)(=O)O |
| Propionylcarnitine | 107738 | HMDB0000824 | C03017 | CCC(=O)OC(CC(=O)[O-])C[N+](C)(C)C |
| Pseudouridine | 15047 | HMDB0000767 | C02067 | C1=C(C(=O)NC(=O)N1)[C@H]2[C@@H]([C@@H]([C@H](O2)CO)O)O |
| Pyrocatechol sulfate | 3083879 | HMDB0059724 | - | OC1=C(OS(O)(=O)=O)C=CC=C1 |
| Stearoylcarnitine | 3006797 | HMDB0000848 | - | CCCCCCCCCCCCCCCCCC(=O)O[C@H](CC(=O)O)C[N+](C)(C)C |
| Succinyladenosine | 20849086 | HMDB0000912 | - | C1=NC2=C(C(=N1)N[C@@H](CC(=O)O)C(=O)O)N=CN2[C@H]3[C@@H]([C@@H]([C@H](O3)CO)O)O |
| Succinylcarnitine | 71464481 | HMDB0061717 | - | C[N+](C)(C)C[C@@H](CC([O-])=O)OC(=O)CCC(O)=O |
| Sulfotyrosine | 514186 | - | - | C1=CC(=CC=C1C[C@@H](C(=O)O)N)OS(=O)(=O)O |
| Uracil | 1174 | HMDB0000300 | - | O=C1NC=CC(=O)N1 |
| Urea | 1176 | HMDB0000294 | C00086 | C(=O)(N)N |
| Uric acid | 1175 | HMDB0000289 | C00366 | C12=C(NC(=O)N1)NC(=O)NC2=O |
| Uridine | 6029 | HMDB0000296 | C00299 | C1=CN(C(=O)NC1=O)[C@H]2[C@@H]([C@@H]([C@H](O2)CO)O)O |
| Valerylcarnitine | 53481619 | HMDB0013128 | - | CCCCC(=O)O[C@@H](CC(=O)[O-])C[N+](C)(C)C |
| *α-N*-Phenylacetyl-L-glutamine | 92258 | HMDB0006344 | C04148 | C1=CC=C(C=C1)CC(=O)N[C@@H](CCC(=O)N)C(=O)O |
| *γ*-Butyrobetaine | 725 | HMDB0006831 | C01181 | C[N+](C)(C)CCCC(=O)[O-] |
